# Stability of Inverse Problems for Steady Supersonic Flows Past Lipschitz Perturbed Cones

**DOI:** 10.1007/s00205-025-02137-5

**Published:** 2025-11-20

**Authors:** Gui-Qiang G. Chen, Yun Pu, Yongqian Zhang

**Affiliations:** 1 Mathematical Institute, University of Oxford, Woodstock Road, Oxford, OX2 6GG UK; 2 School of Mathematical Sciences, Fudan University, 220 Handan Road, Shanghai, 200433 China; 3 Academy of Mathematics and Systems Science, Chinese Academy of Sciences, No. 55 Zhongguancun East Road, Beijing, 100190 China

## Abstract

We are concerned with inverse problems for supersonic potential flows past infinite axisymmetric Lipschitz cones. The supersonic flows under consideration are governed by the steady isentropic Euler equations for axisymmetric potential flows, which give rise to a singular geometric source term. We first study the inverse problem for the stability of an oblique conical shock as an initial-boundary value problem with both the generating curve of the cone surface and the leading conical shock front as free boundaries. We then establish the existence and asymptotic behavior of global entropy solutions with bounded BV norm of the inverse problem, under the condition that the Mach number of the incoming flow is sufficiently large and the total variation of the pressure distribution on the cone is sufficiently small. To this end, we first develop a modified Glimm-type scheme to construct approximate solutions by self-similar solutions as building blocks to balance the influence of the geometric source term. Then we define a Glimm-type functional, based on the local interaction estimates between weak waves, the strong leading conical shock, and self-similar solutions. Meanwhile, the approximate generating curves of the cone surface are also constructed. Next, when the Mach number of the incoming flow is sufficiently large, by asymptotic analysis of the reflection coefficients in those interaction estimates, we prove that appropriate weights can be chosen so that the corresponding Glimm-type functional decreases in the flow direction. Finally, we determine the generating curves of the cone surface and establish the existence of global entropy solutions containing a strong leading conical shock, besides weak waves. Moreover, the entropy solution is proved to approach asymptotically the self-similar solution determined by the incoming flow and the asymptotic pressure on the cone surface at infinity.

## Introduction

**Fig. 1:**
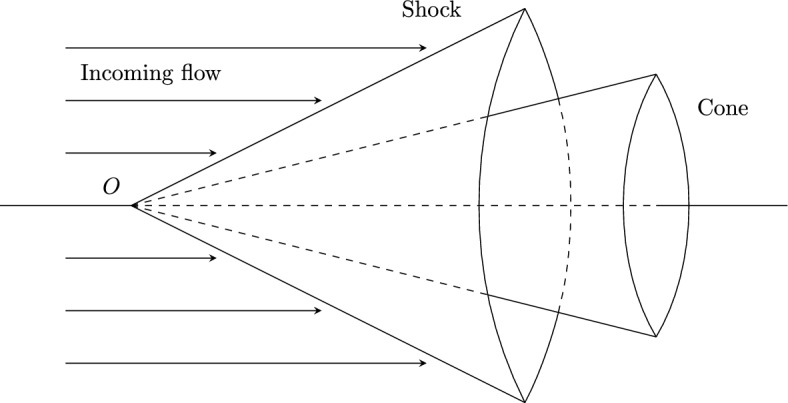
The strong straight-sided conical shock

**Fig. 2:**
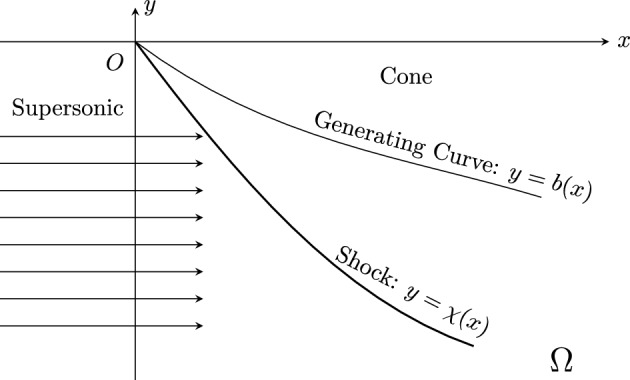
Supersonic flow past an axisymmetric cone

We are interested in the structural stability of inverse problems for the three-dimensional (3-D) steady supersonic potential flows past a Lipschitz perturbed cone with given states of the incoming flow together with Lipschitz perturbed pressure distributions on its surface. The shock stability problem of steady supersonic flows past Lipschitz cones is fundamental for the mathematical theory of the multidimensional (M-D) hyperbolic systems of conservation laws, since its solutions are time-asymptotic states and global attractors of general entropy solutions of time-dependent initial-boundary value problems (IBVP) with abundant nonlinear phenomena, besides its significance to many fields of applications including aerodynamics; see [3, 9, 20, 29] and references cited therein. Meanwhile, the corresponding inverse problems play essential roles in airfoil design; see [1, 2, 5, 26–28, 39, 40, 43, 45]. As indicated in [20], when a uniform supersonic flow of constant speed from the far-field (negative infinity) hits a straight cone, given a constant pressure distribution that is less than a critical value on the cone surface, the vertex angle of the cone can be determined such that there is a supersonic straight-sided conical shock attached to the cone vertex, and the state between the conical shock-front and the cone can be obtained by the shooting method, which is a self-similar solution; see Fig. 1. In this paper, we focus our analysis on the stability of an inverse problem, along with the background self-similar solutions, in the steady potential flows that are axisymmetric with respect to the *x*–axis, given the pressure distributions of gas on the cones, whose boundary surfaces in ${\mathbb {R}}^3$, formed by the rotation of generating curves of the form $\Gamma := \{(x,b(x))\, : \, x\geqq 0\}$ around the *x*–axis, are to be determined; see Fig. 2.

To be more precise, the governing 3-D Euler equations for steady potential conical flows are of the form

1.1 $$\begin{aligned} \left\{ \begin{aligned}&(\rho u)_x+(\rho v)_y=-\frac{\rho v}{y}, \\&v_x-u_y=0, \end{aligned} \right. \end{aligned}$$

together with the Bernoulli law

1.2 $$\begin{aligned} \frac{u^2+v^2}{2}+\frac{c^2}{\gamma -1}= \frac{u_{\infty }^2}{2}+\frac{c_{\infty }^2}{\gamma -1}, \end{aligned}$$

where $U:=(u,v)^\top$ is the velocity in the (*x*, *y*)–coordinates, $\rho$ is the flow density, and $U_{\infty }=(u_{\infty },0)^\top$ and $\rho _{\infty }$ are the velocity and the density of the incoming flow, respectively. The Bernoulli law in (1.2) is obtained via the constitutive relation between pressure *p* and density $\rho$ by scaling

$$\begin{aligned} p=\rho ^{\gamma }, \end{aligned}$$

with $\gamma >1$ for the polytropic isentropic gas and $\gamma =1$ for the isothermal flow. In particular, $c=:\sqrt{\frac{\gamma p}{\rho }}$ is called the sonic speed, and $M:=\sqrt{\frac{u^2+v^2}{c^2}}$ is called the Mach number.

The Bernoulli law (1.2) can be written as

1.3 $$\begin{aligned} \frac{u^2+v^2}{2}+\frac{(u^2+v^2)M^{-2}}{\gamma -1}= \frac{u_\infty ^2}{2}+\frac{u_\infty ^2M_{\infty }^{-2}}{\gamma -1}. \end{aligned}$$

Without loss of generality, we may choose $u_\infty =1$ by scaling; otherwise, we can simply scale: $U\rightarrow u_\infty ^{-1}U$, in system (1.1) and (1.3). With fixed $u_\infty =1$, then $M_{\infty }\rightarrow \infty$ is equivalent to $p_{\infty }\rightarrow 0$, or $c_{\infty }\rightarrow 0$.

System (1.1) can be written in the form

1.4 $$\begin{aligned} \partial _xW(U)+\partial _yH(U)=E(U,y) \end{aligned}$$

with $$U=(u,v)^\top $$, where

$$\begin{aligned} W(U)=(\rho u,v)^\top ,\quad \,\, H(U)=(\rho v,-u)^\top ,\quad \,\, E(U,y)=(-\frac{\rho v}{y},0)^\top , \end{aligned}$$

and $\rho$ is a function of *U* determined by the Bernoulli law (1.2).

When $\rho >0$ and $u>c$, *U* can also be presented by $W(U)=(\rho u,v)^\top$, i.e., $U=U(W)$, by the implicit function theorem, since the Jacobian:

$$\begin{aligned} \det \big (\nabla _UW(U)\big )=-\frac{\rho }{c^2}(u^2-c^2)<0. \end{aligned}$$

Regarding *x* as the *time* variable, (1.4) can be written as

1.5 $$\begin{aligned} \partial _xW+\partial _yH(U(W))=E(U(W),y). \end{aligned}$$

Therefore, system (1.1)–(1.2) becomes a hyperbolic system of conservation laws with source terms of form (1.5). Such nonhomogeneous hyperbolic systems of conservation laws also arise naturally in other problems from many important applications, which exhibit rich phenomena; for example, see [9, 11–13, 20, 23] and the references cited therein.

Throughout this paper, the following conditions are assumed:

- **(A1)** $$\,\,p^b(x)>0$$ for $$x>0$$,
  $$\begin{aligned} p^{b}(x)=p_{0}\qquad \text{ for } x\in [0,x_{0}], \end{aligned}$$
  where $x_{0}>0$, $p_0\in (0, p^*)$ for some $p^*>0$ to be determined by $\gamma >1$, and
  $$\begin{aligned} p^{b}\in \text {BV}([0,\infty )). \end{aligned}$$
- **(A2)**   The velocity of the incoming flow $U_{\infty }=(1,0)^\top$ is supersonic: $M_{\infty }>1$.

Given a perturbed pressure distribution $p^b(x)$ on the cone surface, the problem is axisymmetric with respect to the $x-$axis. Thus, it suffices to analyze the problem in the half-space $\{y\leqq 0\}$. Then the inverse problem is to find the generating curve $y=b(x)\leqq 0$ of the cone surface and a global solution in the domain:

1.6 $$\begin{aligned} \Omega =\big \{(x,y)\,:\,x\geqq 0,y<b(x)\big \} \end{aligned}$$

with its upper boundary:

1.7 $$\begin{aligned} \Gamma =\big \{(x,y)\,:\,x\geqq 0,y= b(x)\big \} \end{aligned}$$

such that

1.8 $$\begin{aligned} U\cdot {{\textbf {n}}}|_{\Gamma }=0, \end{aligned}$$

where ${{\textbf {n}}}={{\textbf {n}}}\big (x,b(x)\big ) =\frac{(-b'(x),1)^\top }{\sqrt{1+(b'(x))^2}}$ is the corresponding outer normal vector to $\Gamma$ at a differentiable point $(x,b(x))\in \Gamma$.

With this setup, the inverse stability problem can be formulated into the following initial-boundary value problem (IBVP) for system (1.4):

**Cauchy Condition:**

1.9 $$\begin{aligned} U|_{x=0}=U_{\infty }:= (1,0)^\top , \end{aligned}$$

**Boundary Condition:**

1.10 $$\begin{aligned} p\big (x,b(x)\big )=p^b(x). \end{aligned}$$

We first introduce the notion of entropy solutions for problem (1.5)–(1.10).

### Definition 1.1

(**Entropy Solutions**).   Consider the inverse problem (1.5)–(1.10). A function $b(x)\in \text {Lip}([0,\infty ))$ is called a generating curve $\Gamma$ of the cone surface as defined in (1.7), and a vector function $U=(u,v)^\top \in (\text {BV}_{\textrm{loc}}\cap \text {L}^{\infty })(\Omega )$ with $\Omega$ defined in (1.6) is called an entropy solution of (1.5)–(1.10) if they satisfy the following conditions:

(i) For any test function $\phi \in \text {C}_{0}^1({\mathbb {R}}^2;{\mathbb {R}})$ and $\psi \in \text {C}_{0}^1(\Omega ;{\mathbb {R}})$,
  1.11 $$\begin{aligned} \begin{aligned}&\iint _{\Omega }\big (\rho u\phi _x+\rho v\phi _y-\dfrac{\rho v}{y}\phi \big )\,{\textrm{d}}x {\textrm{d}}y +\int _{-\infty }^{0}\phi (0,y)\rho _{\infty }u_{\infty }\,{\textrm{d}}y=0,\\&\iint _{\Omega }\left( v\psi _x-u\psi _y\right) {\textrm{d}}x {\textrm{d}}y=0, \end{aligned} \end{aligned}$$
(ii) For any convex entropy pair $({\mathcal {E}},{\mathcal {Q}})$ with respect to *W* of (1.5), i.e., $\nabla ^2{\mathcal {E}}(W)\geqq 0$ and $\nabla {\mathcal {Q}}(W)=\nabla {\mathcal {E}}(W)\nabla H(U(W))$,
  1.12 $$\begin{aligned} \begin{aligned}&\iint _{\Omega }\big ({\mathcal {E}}(W(U))\varphi _x+{\mathcal {Q}}(W(U))\varphi _y +\nabla _W{\mathcal {E}}(W(U))E(U,y)\varphi \big )\,{\textrm{d}}x {\textrm{d}}y\\&\quad \quad +\int _{-\infty }^{0}{\mathcal {E}}(W(U_{\infty }))\varphi (0,y)\,{\textrm{d}}y\geqq 0 \end{aligned} \end{aligned}$$
  for any $\varphi \in \text {C}_{0}^1(\Omega ;{\mathbb {R}})$ with $\varphi \geqq 0$.

### Remark 1.1

 For the potential flow, the Bernoulli law (1.2) gives

$$\begin{aligned} \frac{M^2}{2}+\frac{1}{\gamma -1}=\frac{B_\infty }{c^2} \end{aligned}$$

for $B_\infty =\frac{u_\infty ^2}{2}+\frac{c_\infty ^2}{\gamma -1}$, $c^2=\gamma \rho ^{\gamma -1}$, and $p=\rho ^\gamma$. Then the assumptions on pressure $p^b$ can be reduced to the equivalent ones on the Mach number $M_b$ on the unknown boundary $\Gamma$.

### Main Theorem

(Existence and stability).  Let **(A1)**–**(A2)** hold, and let $1<\gamma <3$ and

$$\begin{aligned} 0<p_{0}<p^*:=\Big ((\sqrt{\gamma +7}-\sqrt{\gamma -1}) \sqrt{\frac{\gamma -1}{16\gamma }} \Big )^\frac{2\gamma }{\gamma -1}. \end{aligned}$$

Assume that $M_{\infty }$ is sufficiently large and $\varepsilon _0$ is sufficiently small such that

1.13 $$\begin{aligned} \begin{aligned} \text {T.V.}\,\{p^{b}\}=\varepsilon _p<\varepsilon _0. \end{aligned} \end{aligned}$$

Then there exists a constant $C>0$, depending only on $p_0$ and the system, such that the following statements hold:

(i) *Global existence*: IBVP (1.4)–(1.10) determines a boundary $y=b(x)=\int _{0}^{x}b'_+(t)\,{\textrm{d}}t$ with $b'_+\in$BV$({\mathbb {R}}_{+})$ satisfying
  1.14 $$\begin{aligned} \sup _{x>0}|b'_+(x)-b_0|<C\varepsilon _p \end{aligned}$$
  and admits a global entropy solution *U*(*x*, *y*) with bounded total variation:
  1.15 $$\begin{aligned} \sup _{x>0}\text {T.V.}\,\{U(x,y)\, :\, -\infty<y<b(x)\}<\infty \end{aligned}$$
  in the sense of Definition 1.1. Moreover, the solution contains a strong leading shock-front $y=\chi (x)=\int _{0}^xs(t){\textrm{d}}t$, where $s\in$BV$({\mathbb {R}}_{+})$ satisfies
  1.16 $$\begin{aligned} \sup _{x>0} |s(x)-s_0|<C\varepsilon _p, \end{aligned}$$
  and the solution between the leading shock-front and the cone surface satisfies
  1.17 $$\begin{aligned} \sup _{x>0}\text {T.V.}\{U(x,y)\, :\, \chi (x)<y<b(x)\}<C(\varepsilon _p + b_0 - s_0) . \end{aligned}$$
  Here above, $s_0$ denotes the slope of the corresponding straight-sided shock-front and $b_{0}$ is the slope of the generating curve of the straight-sided cone surface.
(ii) *Asymptotic behavior*: For the entropy solution *U*(*x*, *y*),
  1.18 $$\begin{aligned} \lim _{x\rightarrow \infty }\sup \left\{ \big |U(x,y)-{\tilde{U}} (\sigma ;s_{\infty },G(s_{\infty }))\big |\,:\,\, \chi (x)<y<b(x)\right\} =0 \end{aligned}$$
  with $${\tilde{U}}(\sigma ;s_{\infty },G(s_{\infty }))$$ satisfying $${\tilde{U}}(s_{\infty };s_{\infty },G(s_{\infty }))=G(s_{\infty })$$,
  1.19 $$\begin{aligned} \begin{aligned}&{\tilde{U}}(b_{\infty }';s_{\infty },G(s_{\infty }))\cdot (-b_{\infty }',1)=0,\\&\dfrac{1}{2}\big |{\tilde{U}}(b_{\infty }';s_{\infty },G(s_{\infty }))\big |^2 +\dfrac{\gamma (p^{b}_{\infty })^{\frac{\gamma -1}{\gamma }}}{\gamma -1} =\dfrac{1}{2}+\dfrac{\gamma p_{\infty }^{\frac{\gamma -1}{\gamma }}}{\gamma -1}, \end{aligned} \end{aligned}$$
  where
  1.20 $$\begin{aligned} \begin{aligned} p^{b}_{\infty }=\lim _{x\rightarrow \infty }p^{b}(x),\quad \,\, s_{\infty }=\lim _{x\rightarrow \infty }s(x),\quad \,\, b'_{\infty }=\lim _{x\rightarrow \infty }b'_{+}(x), \end{aligned} \end{aligned}$$
  ${\tilde{U}}(\sigma ;s,G(s))$ is the state of the self-similar solution, and *G*(*s*) denotes the state connected to state $U_{\infty }$ by the strong leading shock-front of speed *s*.

During the last forty years, the shock stability problem has been studied for the perturbed cones with small perturbations of the straight-sided cone. For polytropic potential flow near the cone vertex, the local existence of piecewise smooth solutions was established in [15, 17] for both symmetrically perturbed cone and pointed body, respectively. Lien-Liu in [38] first analyzed the global existence of weak solutions via a modified Glimm scheme for the uniform supersonic isentropic Euler flow past over a piecewise straight-side cone, provided that the cone has a small opening angle (the initial strength of the shock-front is relatively weak) and the Mach number of the incoming flow is sufficiently large. Later on, Wang-Zhang considered in [48] for supersonic potential flow for the adiabatic exponent $\gamma \in (1,3)$ over a symmetric Lipschitz cone with an arbitrary opening angle less than the critical angle and constructed global weak solutions that are small perturbations of the self-similar solution, given that the total variation of the slopes of the perturbed generating curves of the cone is sufficiently small and the Mach number of the incoming flow is sufficiently large. In addition, for the isothermal flows (i.e., $\gamma =1$), Chen-Kuang-Zhang in [10] made full use of delicate expansions up to second-order as the Mach number of the incoming flow goes to infinity and provided a complete proof of the global existence and asymptotic behavior of conical shock-front solutions in BV when the isothermal flow passes through the Lipschitz perturbed cones that are small perturbations of the straight-sided one.

When the surface of the perturbed cone is smooth, using the weighted energy methods, Chen-Xin-Yin established the global existence of piecewise smooth solutions in [19]. They considered a 3-D axisymmetric potential flow past a symmetrically perturbed cone under the assumption that the attached angle is sufficiently small and the Mach number of the incoming flow is sufficiently large. This result was also extended to the M-D potential flow case; see [32] for more details. Under a certain boundary condition on the cone surface, the global existence of the M-D conical shock solutions was obtained in [49] when the uniform supersonic incoming flow with large Mach number passes a generally curved sharp cone. Meanwhile, using a delicate expansion of the background solution, Cui-Yin established the global existence and stability of a steady conical shock wave in [21, 22] for the symmetrically perturbed supersonic flow past an infinitely long cone whose vertex angle is less than the critical angle. More recently, by constructing new background solutions that allow the speeds of the incoming flows to approach the limit speed, the global existence of steady symmetrically conical shock solutions was established in Hu-Zhang [29] when a supersonic incoming potential flow hits a symmetrically perturbed cone with an opening angle less than the critical angle. We also remark that some pivotal results have been obtained on the stability of M-D transonic shocks under symmetric perturbations of the straight-sided cones or the straight-sided wedges, as well as on Radon measure solutions for steady compressible Euler equations of hypersonic-limit conical flows; see [6–8, 42, 50] and the references cited therein.

Corresponding to these shock stability problems, two types of inverse problems have been considered. One type is for the problems of determining the shape of the wedge in the planar steady supersonic flow for the given location of the leading shock front. This kind of inverse problems and the related inverse piston problems have been considered by Li-Wang in [34–37, 46, 47], where the leading shock-front is assumed to be smooth and the characteristic method is applied to find the piecewise smooth solution with the leading shock as its only discontinuity; see also [33]. The other one is for the problems of determining the shape of a wedge or a cone with given pressure distribution on it in the planar steady supersonic flow (*cf*. [41]) or axisymmetric conical steady supersonic flow. Though various numerical methods and the linearized method have been proposed to deal with this type of problems, there seems no rigorous result on the existence of solutions to such inverse problems for steady supersonic flow past a cone.

In this paper, we develop a modified Glimm scheme to establish the global existence and the asymptotic behavior of conical shock-front solutions of the inverse problem in *BV* in the flow direction, when the isentropic flow passes through the cones with given pressure distributions on their surfaces, which are small perturbations of a constant pressure less than the critical value. Mathematically, our problem can be formulated as a free boundary problem governed by 2-D steady isentropic irrotational Euler equations with geometric structure.

There are two main difficulties in solving this problem: one of them is the singularity generated by the geometric source term, and the other is that, compared to the shock stability problem for supersonic flows past a cone, the generating curve of the cone is unknown. For supersonic flows past an axisymmetric cone with the given generating curve, a modified Glimm scheme developed by Lien-Liu in [38] is used to construct approximate solutions (see also [10, 48]). In the previous construction, in order to incorporate with the geometric source term and the boundary condition on the approximate generating curve, the center $(x_0,0)$ of the self-similar variable $\sigma =\frac{x-x_0}{y}$ is defined to be the intersection of the *x*-axis and the line on which the current approximate generating curve (a line segment of a polyline) lies, and the center is changed according to the random choice at each step when the ordinary differential equations (2.3) are solved. As a result, the approximate solution on the approximate generating curve is a piecewise constant vector-valued function that satisfies the boundary condition everywhere. However, in the inverse problem under consideration in this paper, the generating curve of the cone is to be determined, *apriori* unknown, so that the approach in Lien-Liu (*cf.* [38]) could not apply directly.

To overcome the new difficulties, we first fix the center of the self-similar variable to be the origin when solving the differential equations (2.3) and then develop a modified Glimm scheme to construct approximate solutions $U_{\Delta x,\vartheta }(x,y)$ via the self-similar solutions as building blocks in order to incorporate the geometric source term. In our construction, the grid points are fixed at the beginning, which are the intersections of lines $x=x_h$, $h\in {\mathbb {N}}$, and the rays issuing from the origin (the vertex point of the cone). Consequently, this construction allows us to find a new term $\theta _{b}(h)$ to control the increasing part of the Glimm type functional near the approximate boundary (see Lemma 4.6), while it brings us an extra error so that the boundary conditions on the approximate boundary are no longer satisfied everywhere, but are satisfied at the initial point of each approximate boundary at each step. Nevertheless, in Proposition 6.3, we are able to prove that this error goes to zero as the grid size $\Delta x$ tends to zero.

Furthermore, we make careful asymptotic expansions of the self-similar solutions with respect to $M_{\infty }^{-1}$. We then make full use of the asymptotic expansion analysis of the background solutions with respect to $M_{\infty }^{-1}$ to calculate the reflection coefficients $K_{r,1}$, $K_{w,2}$, $K_s$, and $\mu _{w,2}$ of the weak waves reflected from both the boundary and the strong leading shock, and of the self-similar solutions reflected from the strong leading shock to prove that

$$\begin{aligned} \lim _{M_{\infty }\rightarrow \infty }\big (|K_{r,1}||K_{w,2}|+|K_{r,1}||K_s||\mu _{w,2}|\big )<1. \end{aligned}$$

Based on this, we choose some appropriate weights, independent of $M_{\infty }$, in the construction of the Glimm-type functional and show that the functional is monotonically decreasing. Then the convergence of the approximate solutions is followed by the standard approach for the Glimm-type scheme as in [24, 31]; see also [4, 14, 18, 23, 44]. Finally, the existence of entropy solutions and the asymptotic behavior of the entropy solutions are also proven.

The remaining part of this paper is organized as follows: In Section 2, we give some preliminaries of the homogeneous system (1.1) and then study Riemann-type problems in several cases and self-similar solutions of the unperturbed conic flow. Also, we calculate the limit states of related quantities as $M_{\infty } \rightarrow \infty$. In Section 3, we construct a family of approximate solutions via a modified Glimm scheme. In Section 4, we establish some essential interaction estimates in a small neighborhood in the limit state. Then, in Section 5, we define the Glimm-type functional and show the monotonicity of the Glimm-type functional and, in Section 6, we prove that there exists a subsequence of approximate solutions converging to the entropy solution. Finally, in Section 7, we give the asymptotic behavior of the entropy solution which, together with the existence theory, leads to our main theorem.

## Riemann Problems and Self-Similar Solutions of the Unperturbed Conic Flow

Regarding *x* as the *time* variable, the simplified system of (1.1):

2.1 $$\begin{aligned} \left\{ \begin{aligned}&(\rho u)_x+(\rho v)_y=0, \\&v_x-u_y=0, \end{aligned} \right. \end{aligned}$$

is strictly hyperbolic with two distinctive eigenvalues:

$$\begin{aligned} \lambda _{i}=\frac{uv+(-1)^ic\sqrt{u^2+v^2-c^2}}{u^2-c^2},\qquad i=1,\ 2, \end{aligned}$$

for $u>c_*$ and $u^2+v^2<q_*^2$, where

$$\begin{aligned} c_*=\sqrt{\frac{\gamma -1}{\gamma +1}+\frac{2c_{\infty }^2}{\gamma +1}}, \quad \, q_*=\sqrt{1+\frac{2c_{\infty }^2}{\gamma +1}}. \end{aligned}$$

Denote $q:=\sqrt{u^2+v^2}$ and $\theta :=\arctan \frac{v}{u}$. Then

$$\begin{aligned} \lambda _{i}=\tan (\theta +(-1)^i\theta _{m}),\qquad i=1, 2, \end{aligned}$$

where

$$\begin{aligned} \theta _{m}:=\arctan (\frac{c}{\sqrt{q^2-c^2}}) \end{aligned}$$

is the Mach angel. A direct computation indicates $\theta _{m}\in (0,\frac{\pi }{2})$.

Next, we introduce the following lemma, whose proof can be found in [48]:

### Lemma 2.1

 For $$u>c_*$$ and $$q<q_*$$,

$$\begin{aligned} \left( -\lambda _{i},1\right) \cdot (\frac{\partial \lambda _{i}}{\partial u},\frac{\partial \lambda _{i}}{\partial v}) = \frac{\gamma +1}{2\sqrt{q^2-c^2}}\sec ^3(\theta +(-1)^i\theta _{m}),\qquad i=1, 2. \end{aligned}$$

Then, setting

$$\begin{aligned} r_{i}(U)=\frac{(-\lambda _{i}(U),1)}{(-\lambda _{i}(U),1)\cdot \nabla \lambda _{i}(U)},\qquad i=1, 2, \end{aligned}$$

we see that $r_{i}(U)\cdot \nabla \lambda _{i}(U)=1$ for $i=1, 2$.

Denote the supersonic part of the shock polar by

$$\begin{aligned} S((u_{\infty },0))=\big \{({\bar{u}},{\bar{v}})\,:\, {\bar{c}}^2< {\bar{u}}^2+{\bar{v}}^2\leqq 1\big \}, \end{aligned}$$

where $({\bar{u}},{\bar{v}})$ satisfies the Rankine–Hugoniot condition

2.2 $$\begin{aligned} \left\{ \begin{aligned}&{\bar{\rho }}({\bar{u}}s-{\bar{v}})=\rho _{\infty }s,\\&{\bar{u}}+{\bar{v}}s=1, \end{aligned} \right. \end{aligned}$$

with $$\frac{{\bar{u}}^2+{\bar{v}}^2}{2}+\frac{\gamma {\bar{\rho }}^{\gamma -1}}{\gamma -1} =\frac{1}{2}+\frac{c_{\infty }^2}{\gamma -1}$$. Let

$$\begin{aligned} S_{1}^-((u_{\infty },0))=\big \{({\bar{u}},{\bar{v}}):\, ({\bar{u}},{\bar{v}})\in S((u_{\infty },0)),\,{\bar{v}}<0\big \} \end{aligned}$$

be the part of shock polar corresponding to the $\lambda _{1}$–characteristic field. Similarly to [30, 51, 52], we can parameterize the shock polar $S_{1}^-((u_{\infty },0))$ by a $\hbox {C}^2$–function:

$$\begin{aligned} G:s\mapsto G(s;U_{\infty }) \qquad \,\,\text{ with } U_{\infty }=(1,0). \end{aligned}$$

Here $G(s;U_{\infty })$ is a supersonic state connected with $U_{\infty }$ by a shock of speed *s*. For simplicity, we write $G(s;U_{\infty })$ as *G*(*s*) and use ${\bar{u}}(s)$ and ${\bar{v}}(s)$ to denote the components of *G*(*s*), that is, $G(s)=({\bar{u}}(s),{\bar{v}}(s))^\top$. Then we have the following property for $S_{1}^-((u_{\infty },0))$ (*cf*. [16]):

### Lemma 2.2

 For $s<\lambda _{1}(U_{\infty })$, $\,{\bar{\rho }}(s)$ is a strictly monotonically decreasing function of *s*, and ${\bar{u}}(s)$ is a strictly monotonically increasing function of *s*.

As in [20], let $\sigma =\frac{y}{x}$. Then the equations in (1.1) become

2.3 $$\begin{aligned} \left\{ \begin{aligned}&\big (1-\frac{u^2}{c^2}\big )\sigma ^2 u_{\sigma } -\frac{2uv}{c^2}\sigma ^2 v_{\sigma } -\big (1-\frac{v^2}{c^2}\big )\sigma v_{\sigma }- v=0, \\&u_{\sigma }+\sigma v_{\sigma }=0. \end{aligned} \right. \end{aligned}$$

or, equivalently,

2.4 $$\begin{aligned} \left\{ \begin{aligned}&u_\sigma =\frac{c^2v}{(1+\sigma ^2)c^2-(v-\sigma u)^2},\\&v_\sigma =-\frac{c^2v}{\sigma \big ((1+\sigma ^2)c^2-(v-\sigma u)^2\big )},\\&\rho _\sigma =\frac{\rho v(v-\sigma u)}{\sigma \big ((1+\sigma ^2)c^2-(v-\sigma u)^2\big )}. \end{aligned} \right. \end{aligned}$$

Given a constant state $({\bar{u}},{\bar{v}})=G(s)$ on $S_{1}^-((u_{\infty },0))$, there exists a local solution ${\tilde{U}}(\sigma ;s,G(s))=({\tilde{u}}(\sigma ;s),{\tilde{v}}(\sigma ;s))$ of system (2.3) with initial data

$$\begin{aligned} ({\tilde{u}}(s;s),\,{\tilde{v}}(s;s))=({\bar{u}},\,{\bar{v}}). \end{aligned}$$

**Fig. 3:**
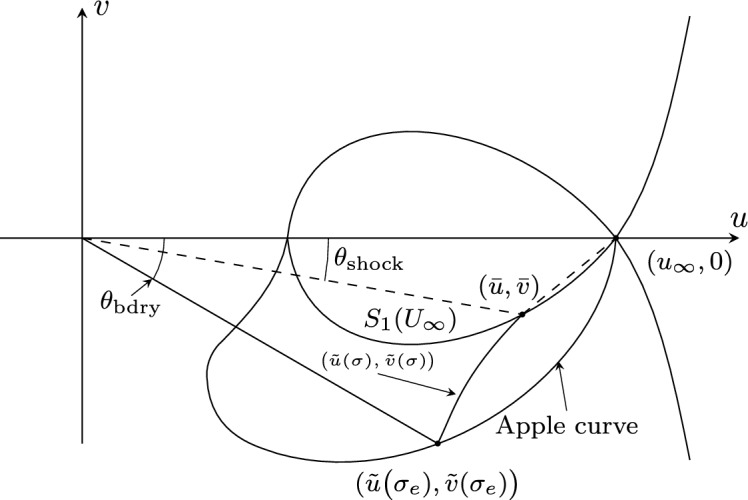
Apple curve

This solution can be extended to an end-point $({\tilde{u}}(\sigma _e;s),\,{\tilde{v}}(\sigma _e;s))$ with $\frac{{\tilde{v}}(\sigma _e;s)}{{\tilde{u}}(\sigma _e;s)}=\sigma _e$. As $({\bar{u}},{\bar{v}})$ varies on $S_{1}^-((u_{\infty },0))$, the collection of these end-states forms an *apple curve through* $U_{\infty }$ (Fig. 3); see [20]. For these solutions, we have following properties, whose proof can be found in [48]:

### Lemma 2.3

 For ${\tilde{u}}(s;s)>{\tilde{c}}(s;s)$ and $\sigma \in (s,\sigma _e)$, then ${\tilde{u}}(\sigma ;s)\sigma -{\tilde{v}}(\sigma ;s)<0$,

$$\begin{aligned}&\frac{\partial {\tilde{u}}}{\partial \sigma }<0,\qquad \frac{\partial {\tilde{v}}}{\partial \sigma }<0,\\&{\tilde{c}}(\sigma ;s)-\frac{{\tilde{v}}(\sigma ;s)-\sigma {\tilde{u}}(\sigma ;s)}{\sqrt{1+\sigma ^2}}>{\tilde{c}}(s;s)-\frac{{\tilde{v}}(s;s)-s{\tilde{u}}(s;s)}{\sqrt{1+s^2}}>0, \end{aligned}$$

with $$\frac{{\tilde{u}}^2+{\tilde{v}}^2}{2}+\frac{{\tilde{c}}^2}{\gamma -1}= \frac{1}{2}+\frac{c_{\infty }^2}{\gamma -1}$$.

Thus, we obtain the following estimate of the self-similar solution $({\tilde{u}}(\sigma ;s),\,{\tilde{v}}(\sigma ;s))$:

### Lemma 2.4

For $${\tilde{u}}(s;s)>{\tilde{c}}(s;s)$$ and $$\sigma \in (s,\sigma _e)$$,

$$\begin{aligned}&\frac{1}{1+s^2}< {\tilde{u}}(\sigma ;s)<{\tilde{u}}(s;s),\\&{\tilde{c}}(s;s)<{\tilde{c}}(\sigma ;s)<{\tilde{c}}(\sigma _e;s)<\sqrt{\frac{(\gamma -1)s^2}{2(1+s^2)}+\frac{1}{M_{\infty }^2}}. \end{aligned}$$

To obtain the asymptotic expansion of the self-similar solution, we need the following properties of the shock polar:

2.5 $$\begin{aligned} p^*=\Big (\big (\sqrt{\gamma +7}-\sqrt{\gamma -1}\big ) \sqrt{\frac{\gamma -1}{16\gamma }} \Big )^\frac{2\gamma }{\gamma -1}. \end{aligned}$$

### Lemma 2.5

Let $1<\gamma <3$ and $p_{0}\in (0,p^*)$. For $M_{\infty }$ large enough, the equations:

2.6 $$\begin{aligned} \left\{ \begin{aligned}&\rho _{0}(u^{\sharp }s^{\sharp }-v^{\sharp })=\rho _{\infty }s^{\sharp },\\&u^{\sharp }+v^{\sharp } s^{\sharp }=1,\\&\frac{(u^{\sharp })^2+(v^{\sharp })^2}{2}+\frac{c_{0}^2}{\gamma -1}=\frac{1}{2}+\frac{c_{\infty }^2}{\gamma -1} \end{aligned} \right. \end{aligned}$$

have a unique solution $(u^{\sharp },v^{\sharp },s^{\sharp })$ with $s^{\sharp }<0$, where $\rho _{\infty }=p_{\infty }^{\frac{1}{\gamma }}$, $\rho _{0}=p_{0}^{\frac{1}{\gamma }}$, and $c_{0}=\sqrt{\gamma } p_{0}^{\frac{\gamma -1}{2\gamma }}$, such that

2.7 $$\begin{aligned} \begin{aligned} \lim _{M_{\infty }\rightarrow \infty }u^{\sharp }&=\lim _{M_{\infty }\rightarrow \infty }u_{a}=1-\frac{2c_{0}^2}{\gamma -1},\\ \lim _{M_{\infty }\rightarrow \infty }v^{\sharp }&=\lim _{M_{\infty }\rightarrow \infty }v_{a} =-\sqrt{\frac{2c_{0}^2}{\gamma -1-2c_{0}^2}}\Big (1-\frac{2c_{0}^2}{\gamma -1}\Big ),\\ \lim _{M_{\infty }\rightarrow \infty } c_{a}^2&= c_{0}^2, \end{aligned} \end{aligned}$$

where

2.8 $$\begin{aligned} u_{a}:=\frac{1}{1+(s^{\sharp })^2},\quad v_{a}:=\frac{s^{\sharp }}{1+(s^{\sharp })^2}, \quad c_{a}:=\sqrt{\frac{(\gamma -1)(s^{\sharp })^2}{2(1+(s^{\sharp })^2)}+\frac{1}{M_{\infty }^2}}. \end{aligned}$$

### Proof

 From the first two equations of (2.6), we have

2.9 $$\begin{aligned} u^{\sharp }=\frac{\rho _{0}+\rho _{\infty }(s^{\sharp })^2}{\rho _{0}(1+s^2)}, \qquad v^{\sharp }=\frac{(\rho _{0}-\rho _{\infty })s^{\sharp }}{\rho _{0}\big (1+(s^{\sharp })^2\big )}. \end{aligned}$$

With the help of the third equation of (2.6), we have

$$\begin{aligned} \bigg ( \frac{\rho _{0}+\rho _{\infty }(s^{\sharp })^2}{\rho _{0}\big (1+(s^{\sharp })^2\big )}\bigg )^2 +\bigg (\frac{(\rho _{0}-\rho _{\infty })s^{\sharp }}{\rho _{0}\big (1+(s^{\sharp })^2\big )} \bigg )^2 =1+\frac{2(c_{\infty }^2-c_{0}^2)}{\gamma -1}, \end{aligned}$$

which gives that

$$\begin{aligned} (s^{\sharp })^2&=\frac{2(c_{0}^2-M_{\infty }^{-2})\rho _{0}^2}{(\gamma -1)\big (\rho _{0}^2-(\gamma M_{\infty }^2)^{-\frac{2}{\gamma -1}}\big )-2(c_{0}^2-M_{\infty }^{-2})\rho _{0}^2}\\&=\frac{2c_{0}^2}{\gamma -1-2c_{0}^2}-\frac{2(\gamma -1)}{(\gamma -1-2c_{0}^2)^2}\, M_{\infty }^{-2} +O(M_{\infty }^{-\frac{4}{\gamma -1}})\qquad \text {as } M_{\infty }\rightarrow \infty . \end{aligned}$$

Therefore, noting that $s^{\sharp }<0$, we obtain

$$\begin{aligned} s^{\sharp }&=-\sqrt{\frac{2c_{0}^2}{\gamma -1-2c_{0}^2}} \bigg (1-\frac{\gamma -1}{2c_{0}^2\left( \gamma -1-2c_{0}^2\right) }\, M_{\infty }^{-2}\bigg ) +O(M_{\infty }^{-\frac{4}{\gamma -1}}) \end{aligned}$$

as $M_{\infty }\rightarrow \infty$. Substituting the above expansion into (2.8)–(2.9) yields (2.7). $\square$

### Lemma 2.6

Let $1<\gamma <3$ and $p_{0}\in (0,p^*)$. For $M_{\infty }$ large enough, there exists $\rho _{d}$ such that

2.10 $$\begin{aligned} \rho _{0}^{\gamma -1}=\frac{\rho _{d}^{\gamma +1}-\rho _{\infty }^{\gamma +1}}{\rho _{d}^2-\rho _{\infty }^2}, \end{aligned}$$

and the equations:

2.11 $$\begin{aligned} \left\{ \begin{aligned}&\rho _{d}(u_{d}s_{d}-v_{d})=\rho _{\infty }s_{d},\\&u_{d}+v_{d}s_{d}=1,\\&\frac{u_{d}^2+v_{d}^2}{2}+\frac{c_{d}^2}{\gamma -1}=\frac{1}{2}+\frac{c_{\infty }^2}{\gamma -1}, \end{aligned} \right. \end{aligned}$$

have a unique solution $(u_{d},v_{d},s_{d})$ with $s_{d}<0$ and $c_{d}=\sqrt{\gamma \rho _{d}^{\gamma -1}}$. Moreover, for

2.12 $$\begin{aligned} u^{\flat }:=\frac{1}{1+s_{d}^2},\quad \, v^{\flat }:=\frac{s_{d}}{1+s_{d}^2}, \end{aligned}$$

we have

2.13 $$\begin{aligned} \begin{aligned} \lim _{M_{\infty }\rightarrow \infty } c_{d}^2&=c_{0}^2,\\ \lim _{M_{\infty }\rightarrow \infty }u^{\flat }&=\lim _{M_{\infty }\rightarrow \infty }u_{d} =1-\frac{2c_{0}^2}{\gamma -1},\\ \lim _{M_{\infty }\rightarrow \infty } v^{\flat }&= \lim _{M_{\infty }\rightarrow \infty } v_{d} =-\sqrt{\frac{2c_{0}^2}{\gamma -1-2c_{0}^2}}\,\Big (1-\frac{2c_{0}^2}{\gamma -1}\Big ). \end{aligned} \end{aligned}$$

### Proof

 For each $\rho _{0}$, it is direct to find $\rho _{d}$ such that (2.10) holds. Moreover, $p_{d}\in (0,p^*)$ when $M_{\infty }$ is large enough. By Lemma 2.5, we obtain the unique solution of (2.11):

2.14 $$\begin{aligned} s_{d}=\frac{2(c_{0}^2-c_{\infty }^2)}{\gamma -1-2(c_{0}^2-c_{\infty }^2)},\quad \, u_{d} =\frac{\rho _{d}+\rho _{\infty }s_{d}^2}{\rho _{d}(1+s_{d}^2)}, \quad \, v_{d}=\frac{(\rho _{d}-\rho _{\infty })s_{d}}{\rho _{d}(1+s_{d}^2)}. \end{aligned}$$

Then we have

$$\begin{aligned} s_{d}&=-\sqrt{\frac{2c_{0}^2}{\gamma -1-2c_{0}^2}} \Big (1-\frac{\gamma -1}{2c_{0}^2(\gamma -1-2c_{0}^2)}\, M_{\infty }^{-2}\Big ) +O(M_{\infty }^{-4}) \end{aligned}$$

as $$M_{\infty }\rightarrow \infty $$.

Substituting the above expansion into (2.12)–(2.14) yields (2.13). $\square$

Given that $p_{0}\in (0,p^{*})$, we now solve the problem for $s_{0}<\sigma <b_{0}$ as

2.15 $$\begin{aligned} {\left\{ \begin{array}{ll} \sigma ^2\big (1-\frac{{\tilde{u}}^2}{{\tilde{c}}^2}\big ) {\tilde{u}}_{\sigma } -\frac{2{\tilde{u}}{\tilde{v}}\sigma ^2}{c^2}{\tilde{v}}_{\sigma } - \big (1-\frac{{\tilde{v}}^2}{{\tilde{c}}^2}\big ) {\tilde{v}}_{\sigma }\sigma -{\tilde{v}}=0,\\ {\tilde{u}}_{\sigma }+\sigma {\tilde{v}}_{\sigma }=0, \end{array}\right. } \end{aligned}$$

with the boundary conditions

2.16 $$\begin{aligned} \begin{aligned}&\,\,\,{\tilde{\rho }}({\tilde{u}}s_{0}-{\tilde{v}})=\rho _{\infty }s_{0},\quad {\tilde{u}}+{\tilde{v}}s_{0}=1 \qquad \,\,  &   \text{ for } \sigma =s_{0},\\&\,\,\,{\tilde{\rho }}=\rho _{0},\quad {\tilde{v}}=b_{0}{\tilde{u}} \qquad \,\,  &   \text{ for } \sigma =b_{0}, \end{aligned} \end{aligned}$$

and define $({\tilde{u}}(\sigma ;s_{0}),{\tilde{v}}(\sigma ;s_{0}))=(1,0)$ for $\sigma <s_{0}$. Indeed, we have

### Lemma 2.7

 Let $1<\gamma <3$ and $p_{0}\in (0,p^{*})$. For $M_{\infty }>K_{1}$, problem (2.15) has a unique solution $({\tilde{u}}(\sigma ;s_{0}),\,{\tilde{v}}(\sigma ;s_{0}))$ containing a supersonic conical shock-front issuing from the vertex. In addition,

2.17 $$\begin{aligned} \begin{aligned}&\lim _{M_{\infty }\rightarrow \infty }(\sigma , {\tilde{c}}^2(\sigma ;s_{0}))=(\tan \theta _{0}, c_{0}^2),\\&\lim _{M_{\infty }\rightarrow \infty }({\tilde{u}}(\sigma ;s_{0}),{\tilde{v}}(\sigma ;s_{0})) =(\cos ^2\theta _{0},\,\sin \theta _{0}\cos \theta _{0}), \end{aligned} \end{aligned}$$

and

2.18 $$\begin{aligned} \lim _{M_{\infty }\rightarrow \infty }\frac{{\tilde{u}}(\sigma ;s_{0})}{{\tilde{c}}(\sigma ;s_{0})} =\frac{\gamma -1-2c_{0}^2}{(\gamma -1)c_{0}}>1,\qquad \cos (\theta _{0}\pm \theta _{m}^{0})>0, \end{aligned}$$

where $\theta _{0}=-\arctan (\sqrt{\frac{2c_{0}^2}{\gamma -1-2c_{0}^2}})$ and $\theta _{m}^{0}=\lim _{M_{\infty }\rightarrow \infty }\theta _{m}$ for $\sigma \in [s_{0},b_{0})$.

### Proof

 Given $p_{0}\in (0,p^{*})$, by the shooting method as in [20], problem (2.15) has a unique solution $({\tilde{u}}(\sigma ;s_{0}),{\tilde{v}}(\sigma ;s_{0}))$ with ${\tilde{u}}(s_{0};s_{0})>{\tilde{c}}(s_{0};s_{0})$, ${\tilde{\rho }}(b_{0};s_{0})=p_{0}^{\frac{1}{\gamma }}$, and ${\tilde{v}}(b_{0};s_{0})=b_{0}{\tilde{u}}(b_{0};s_{0})$.

We then focus on the asymptotic expansions (2.17). Lemma 2.4 indicates that

$$\begin{aligned}&\frac{1}{1+s_{0}^2}<{\tilde{u}}(\sigma ;s_{0})\leqq {\tilde{u}}(s_{0};s_{0}),\\  &\qquad {\tilde{c}}(s_{0};s_{0})\leqq {\tilde{c}}(\sigma ;s_{0})<c_{0} <\sqrt{\frac{(\gamma -1)s_{0}^2}{2(1+s_{0}^2)}+\frac{1}{M_{\infty }^2}} \end{aligned}$$

for $\sigma \in [s_{0},b_{0})$. Meanwhile, it follows from (2.10) that ${\tilde{c}}(s_{0};s_{0})>c_{d}$. Then, due to Lemma 2.2, we see that $u^{\sharp }<{\tilde{u}}(s_{0};s_{0})<u_{d}$ and $s^{\sharp }<s_{0}<0$. Therefore, we have

$$\begin{aligned} c_{d}&<{\tilde{c}}(s_{0};s_{0}) \leqq {\tilde{c}}(\sigma ;s_{0})<c,\\ u_{a}&=\frac{1}{1+(s^{\sharp })^2}<\frac{1}{1+s_{0}^2}< {\tilde{u}}(\sigma ;s_{0})\leqq {\tilde{u}}(s_{0};s_{0})<u_{d}. \end{aligned}$$

From Lemma 2.5–2.6, we have

$$\begin{aligned} \lim _{M_{\infty }\rightarrow \infty }{\tilde{c}}(\sigma ;s_{0})=c_{0}^2, \qquad \lim _{M_{\infty }\rightarrow \infty }{\tilde{u}}(\sigma ;s_{0})=1-\frac{2c_{0}^2}{\gamma -1}. \end{aligned}$$

Since ${\tilde{v}}(\sigma ;s_{0})<0$, from the Bernoulli laws,

$$\begin{aligned} \frac{{\tilde{u}}^2+{\tilde{v}}^2}{2}+\frac{{\tilde{c}}^2}{\gamma -1}= \frac{1}{2}+\frac{c_{\infty }^2}{\gamma -1},\quad \, \frac{(u^{\sharp })^2+(v^{\sharp })^2}{2}+\frac{c_{0}^2}{\gamma -1}=\frac{1}{2}+\frac{c_{\infty }^2}{\gamma -1}, \end{aligned}$$

we conclude

$$\begin{aligned} \lim _{M_{\infty }\rightarrow \infty }{\tilde{v}}(\sigma ;s_{0})=&-\sqrt{\frac{2c_{0}^2}{\gamma -1-2c_{0}^2}}\, \Big (1-\frac{2c_{0}^2}{\gamma -1}\Big ). \end{aligned}$$

Again, by Lemma 2.2, we know that

$$\begin{aligned} \frac{{\tilde{v}}(s_{0};s_{0})}{{\tilde{u}}(s_{0};s_{0})}>b_{0}>\sigma \geqq s_{0}>s^{\sharp }. \end{aligned}$$

Combining all the expansions obtained above, we obtain (2.17).

Furthermore, since

$$\begin{aligned} \lim _{M_{\infty }\rightarrow \infty }\big ( {\tilde{u}}\sqrt{{\tilde{u}}^2+{\tilde{v}}^2-{\tilde{c}}^2}\big )^2 -( {\tilde{v}}{\tilde{c}})^2 =\lim _{M_{\infty }\rightarrow \infty }({\tilde{u}}^2-{\tilde{c}}^2)({\tilde{u}}^2+{\tilde{v}}^2)>0, \end{aligned}$$

we conclude that

$$\begin{aligned} \cos (\theta _{0}\pm \theta ^0_{ma}) =\lim _{M_{\infty }\rightarrow \infty }\cos (\theta \pm \theta _{m}) =\lim _{M_{\infty }\rightarrow \infty }\frac{ {\tilde{u}}\sqrt{{\tilde{u}}^2+{\tilde{v}}^2-{\tilde{c}}^2}\mp {\tilde{v}}{\tilde{c}}}{{\tilde{u}}^2+{\tilde{v}}^2}>0. \end{aligned}$$

This completes the proof. $\square$

Next, for *G*(*s*), we have the following expansions, whose proof can be found in [48]:

### Lemma 2.8

 For $$G(s)=({\bar{u}}(s),{\bar{v}}(s))^\top $$,

$$\begin{aligned}&\lim _{M_{\infty }\rightarrow \infty }({\bar{u}}(s_{0}),\,{\bar{v}}(s_{0})) =(\cos ^2\theta _{0},\,\cos \theta _{0}\sin \theta _{0}), \\&\lim _{M_{\infty }\rightarrow \infty }({\bar{u}}_s(s_{0}),\,{\bar{u}}_s(s_{0})) =(-\sin (2\theta _{0})\cos ^2\theta _{0},\,\cos (2\theta _{0})\cos ^2\theta _{0}), \end{aligned}$$

where $${\bar{u}}_s(s)=\frac{{\textrm{d}}{\bar{u}}(s)}{{\textrm{d}} s}$$ and $${\bar{v}}_s(s)=\frac{{\textrm{d}}{\bar{v}}(s)}{{\textrm{d}} s}$$.

Now, we introduce the elementary wave curves of system (2.1). We denote by $\mathbb {W}(p_{0},p_{\infty })$ the curve formed by ${\tilde{U}}(\sigma ;s_{0})=({\tilde{u}}(\sigma ;s_{0}),{\tilde{v}}(\sigma ;s_{0}))^\top$ for $s_{0}<\sigma <b_{0}$, where $p_{0}$ is the corresponding pressure of the state at the endpoint. As in [48] (also *cf*. [51, 52]), we parameterize the elementary *i*-wave curves for system (2.1) in a neighborhood of $\mathbb {W}(p_{0},p_{\infty })$,

$$\begin{aligned} O_{r}\big (\mathbb {W}(p_{0},p_{\infty })\big )=\bigcup \limits _{s_{0}<\sigma<b_{0}}\big \{U:\ |U-{\tilde{U}}(\sigma ;s_{0})|<r\big \} \qquad \,\text{ for } \text{ some } r>0, \end{aligned}$$

by

$$\begin{aligned} \alpha _{i}\mapsto \Phi _{i}(\alpha _{i};U) \end{aligned}$$

with $$\Phi _{i}\in \text {C}^2$$ and

$$\begin{aligned} \left. \frac{\partial \Phi _{i}}{\partial \alpha _{i}}\right| _{\alpha _{i}=0} =r_{i}(U) \qquad \text{ for } U\in O_{r}(\mathbb {W}(p_{0},p_{\infty })), \,\, i=1,2. \end{aligned}$$

In the sequel, define

$$\begin{aligned} \Phi (\alpha _{1},\alpha _{2};U)=\Phi _{2}(\alpha _{2};\Phi _{1}(\alpha _{1};U)). \end{aligned}$$

Denote ${\tilde{U}}(\sigma ;\sigma _{0},U_{l})$ the solution to the ODE system (2.3) with initial data

$$\begin{aligned} {\tilde{U}}|_{\sigma =\sigma _{0}}=U_{l} \end{aligned}$$

for $U_{l}\in O_{r}\big (\mathbb {W}(p_{0},p_{\infty })\big )$. Then, as in [48], we have

### Lemma 2.9

 For $$p_{0}\in (0,p^{*})$$,

$$\begin{aligned} \lim _{M_{\infty }\rightarrow \infty } \left. \frac{{\textrm{d}}{\tilde{U}}(\sigma ;\sigma _{0})}{{\textrm{d}}\sigma }\right| _{\{\sigma =\sigma _{0},\,U_{l}\in \mathbb {W}(p_{0},p_{\infty })\}} =\big (\sin \theta _{0}\cos ^3\theta _{0},-\cos ^4\theta _{0}\big )^\top . \end{aligned}$$

With all the limits given above, we obtain the following lemma, which is essential in wave-interaction estimates:

### Lemma 2.10

 For $$U_{l}\in \mathbb {W}(p_{0},p_{\infty })$$,

$$\begin{aligned}&\lim _{M_{\infty }\rightarrow \infty }\det \big (r_{1}(U_{l}),r_{2}(U_{l})\big )=\dfrac{4\cos ^2(\theta _{0}+\theta _{m}^{0})\cos ^2(\theta _{0}-\theta _{m}^{0}) \cos ^2\theta _{0}\cos ^2\theta ^{0}_{m}\sin (2\theta _{m}^{0})}{(\gamma +1)^2},\\&\lim _{M_{\infty }\rightarrow \infty }\det \big (G_s(s_{0}),r_{1}\big (G(s_{0})\big )\big ) =-\dfrac{2 \cos ^2(\theta _{0}-\theta _{m}^{0})\cos \theta ^{0}_{m}\cos ^3\theta _{0}\sin (\theta _{0}+\theta _{m}^{0})}{\gamma +1},\\&\lim _{M_{\infty }\rightarrow \infty }\det \big (r_{2}\big (G(s_{0})\big ),G_s(s_{0})\big ) =\dfrac{2 \cos ^2(\theta _{0}+\theta _{m}^{0})\cos \theta ^{0}_{m}\cos ^3\theta _{0}\sin (\theta _{0}-\theta _{m}^{0})}{\gamma +1},\\&\lim _{M_{\infty }\rightarrow \infty }\det \Big (\frac{{\textrm{d}}{\tilde{U}}(\sigma ;\sigma _{0},G(s_{0}))}{{\textrm{d}}\sigma },G_s(s_{0})\Big ) =- \cos ^5\theta _{0}\sin \theta _{0},\\&\lim _{M_{\infty }\rightarrow \infty }\det \Big (r_{2}(U_{l}),\frac{{\textrm{d}}{\tilde{U}}(\sigma ;\sigma _{0},U_{l})}{{\textrm{d}}\sigma }\Big ) =\frac{2}{\gamma +1} \cos ^4\theta _{0}\cos ^2(\theta _{0}+\theta _m^{0})\cos \theta _m^{0}\sin \theta _m^{0},\\&\lim _{M_{\infty }\rightarrow \infty }\det \Big (r_{1}(U_{l}),\frac{{\textrm{d}}{\tilde{U}}(\sigma ;\sigma _{0},U_{l})}{{\textrm{d}}\sigma }\Big ) =-\frac{2}{\gamma +1} \cos ^4\theta _{0}\cos ^2(\theta _{0}-\theta _m^{0})\cos \theta _m^{0}\sin \theta _m^{0}. \end{aligned}$$

Furthermore, we have the following propositions, which will be used in the construction of building blocks of our approximate solutions:

### Proposition 2.1

For $M_{\infty }$ sufficiently large, there exists $\varepsilon _{1}>0$ such that, for any $U_{r}$ and $U_{l}$ lie in $O_{\varepsilon _{1}}(\mathbb {W}(p_{0},p_{\infty }))$, the Riemann problem (2.1) with the initial data

2.19 $$\begin{aligned} U|_{x={\bar{x}}}=\left\{ \begin{aligned}&U_{r}&\,\,\, \text {for }y>{\bar{y}},\\&U_{l}&\,\,\, \text {for }y<{\bar{y}}, \end{aligned} \right. \end{aligned}$$

admits a unique admissible solution consisting of at most two elementary waves $\alpha _{1}$ for the 1-characteristic field and $\alpha _{2}$ for the 2-characteristic field. Moreover, states $U_{l}$ and $U_{r}$ are connected by

$$\begin{aligned} U_{r}=\Phi (\alpha _{1},\alpha _{2};U_{l}). \end{aligned}$$

### Proposition 2.2

 For $M_{\infty }$ sufficiently large, there exists $\varepsilon _{2}>0$ such that, for any $U_{l}\in O_{\varepsilon _{2}}( \mathbb {W}(p_{0},p_{\infty }))$ and $p_{1}$, $p_{2}\in O_{\varepsilon _{2}}(p_{0})$, there is $\delta _{1}$ solving the equation

2.20 $$\begin{aligned} \dfrac{1}{2}|\Phi (\delta _{1},0;U_{l})|^2+\dfrac{\gamma }{\gamma -1}p_{2}^{\frac{\gamma -1}{\gamma }} =\dfrac{1}{2}|U_{l}|^2+\dfrac{\gamma }{\gamma -1}p_{1}^{\frac{\gamma -1}{\gamma }}. \end{aligned}$$

### Proof

 From (2.20), we have

$$\begin{aligned} \lim _{M_{\infty }\rightarrow \infty }\frac{1}{2}\, \left. \frac{\partial |\Phi (\delta _{1},0;U_{l})|^2}{\partial \delta _{1}}\right| _{\delta _{1}=0} =U_{l}\cdot r_{1}(U_{l})\ne 0. \end{aligned}$$

By the implicit function theorem, there exists $\delta _{1}$ such that (2.20) holds, provided $\varepsilon _{2}$ sufficiently small. $\square$

### Proposition 2.3

For $M_{\infty }$ sufficiently large, there exists $\varepsilon _{3}>0$ such that, for any $U_{l}=U_{\infty }$ and $U_{r}\in O_{\varepsilon _{3}}( \mathbb {W}(p_{0},p_{\infty }))\cap O_{\varepsilon _{3}}(G(s_{0}))$, the Riemann problem (2.1) with initial data (2.19) admits a unique admissible solution that contains a strong 1-shock $s_{1}$ and a 2-weak wave $\beta _{2}$ of the 2-characteristic field. Moreover, states $U_{l}$ and $U_{r}$ are connected by

2.21 $$\begin{aligned} U_{r}=\Phi _{2}(\beta _{2};G(s_{1};U_{l})). \end{aligned}$$

### Proof

 It follows from (2.21) and Lemma 2.10 that

$$\begin{aligned} \begin{aligned}&\lim _{M_{\infty }\rightarrow \infty } \det \Big ( \frac{\partial \Phi _{2}(\beta _{2};G(s_{1};U_{l}))}{\partial (s_{1},\beta _{2})}\Bigg | _{\{s_{1}=s_{0}, \beta _{2}=0\}}\Big ) \\  &\quad =-\lim _{M_{\infty }\rightarrow \infty }\det \big (r_{2}(G(s_{0}),G_s(s_{0}))\big )\ne 0. \end{aligned} \end{aligned}$$

The existence of the solution of this Riemann problem is ensured by the implicit function theorem for $\varepsilon _{3}$ sufficiently small. $\square$

To end this section, we introduce the following interaction estimate given by Glimm [24] for weak waves (see also [44, 48, 52]):

### Lemma 2.11

Let $U_{l}\in \mathbb {W}(p_{0},p_{\infty })$, $\alpha$, $\beta$, and $\delta$ satisfy

$$\begin{aligned} \Phi (\delta _{1},\delta _{2};U_{l})=\Phi (\beta _{1},\beta _{2};\Phi (\alpha _{1},\alpha _{2};U_{l})). \end{aligned}$$

Then

$$\begin{aligned} \delta =\alpha +\beta +O(1)Q^0(\alpha ,\beta ), \end{aligned}$$

where $Q^0(\alpha ,\beta )=\sum \{|\alpha _{i}||\beta _{i}|:\alpha _{i}\text { and }\beta _{j}\text { approach}\}$, and *O*(1) depends continuously on $M_{\infty }<\infty$.

## Approximate Solutions

In this section, we construct approximate solutions for system (1.4) with (1.8)–(1.9) by a modified Glimm scheme. Compared to the modified Glimm scheme developed in [10, 38, 48], in our construction, the grid points are fixed at the very beginning, which are independent of the approximate solution and the random choice.

Given $\varepsilon >0$ and $\Delta x>0$, there exist piecewise constant functions $p^{b}_{\Delta x}$ such that

$$\begin{aligned} \text {T.V.}\,\{p^{b}_{\Delta x}(\cdot )\}\leqq \text {T.V.}\,\{p^{b}(\cdot )\}, \qquad \Vert p^{b}_{\Delta x}-p^{b}\Vert _{{{\textbf {L}}}^\infty }\leqq \varepsilon , \end{aligned}$$

where

$$\begin{aligned} p^{b}_{\Delta x}(x)={\left\{ \begin{array}{ll} p^{b}_{\Delta x,0}=p_{0} \,\,\,& \text { for }x\in [0,x_{0}),\\ p^{b}_{\Delta x,h+1} \,\,\,& \text { for }x\in [x_{h},x_{h+1})\text { and }h\in {\mathbb {N}}, \end{array}\right. } \end{aligned}$$

with $p^{b}_{\Delta x,h+1}$ being constants on the corresponding intervals and $x_{h}=x_{0}+h\Delta x$ for $h\in {\mathbb {N}}$. Then, from Lemma 2.7, for $p^{b}_{\Delta x,0}=p_{0}$, there exists $\big ({\tilde{u}}(\sigma ;s_{0}),{\tilde{v}}(\sigma ;s_{0})\big )$ such that ${\tilde{p}}(b_{0};s_{0})=p_{0}$ and ${\tilde{v}}(b_{0};s_{0})=b_{0}{\tilde{u}}(b_{0};s_{0})$.

We now define the difference scheme. Choose $\vartheta =(\vartheta _{0},\vartheta _{1},\vartheta _{2},\dots ,\vartheta _{h},\dots )$ randomly in [0, 1). For $0< x< x_{0}$, let

$$\begin{aligned} b_{\Delta x,\vartheta }(x)=b_{0}x,\qquad \,\, \chi _{\Delta x,\vartheta }(x)=s_{0}x. \end{aligned}$$

We denote $\Gamma _{\Delta x,\vartheta ,0}=\big \{(x,b_{\Delta x,\vartheta }(x)):\, 0\leqq x< x_{0}\big \}$, $S_{\Delta x,\vartheta ,0}=\big \{(x,\chi _{\Delta x,\vartheta }(x)):\, 0\leqq x< x_{0}\big \}$, and $\Omega _{\Delta x,\vartheta ,0}=\big \{(x,y):\, y<b_{\Delta x,\vartheta }(x), \, 0\leqq x< x_{0}\big \}$. In region $\Omega _{\Delta x,\vartheta ,0}$, we then define that

$$\begin{aligned}&U_{\Delta x,\vartheta }(x,y)\\&\quad = {\left\{ \begin{array}{ll} (u_{\Delta x,\vartheta }(x,y),v_{\Delta x,\vartheta }(x,y))^\top \triangleq ({\tilde{u}}(\sigma ;s_{0}),{\tilde{v}}(\sigma ;s_{0}))^\top \,\,\, & \text {for }\frac{y}{x}=\sigma \in (s_{0},b_{0}),\\ U_{\infty } \quad & \text {for }\frac{y}{x}=\sigma <s_{0}, \end{array}\right. } \end{aligned}$$

and, on boundary $\Gamma _{\Delta x,\vartheta ,0}$, we set that

$$\begin{aligned} U_{\Delta x,\vartheta }(x, b_{\Delta x,\vartheta }(x))&=U_{\Delta x,\vartheta }^b(x)=(u_{\Delta x,\vartheta }^b(x),v_{\Delta x,\vartheta }^b(x))^\top \\&\triangleq ({\tilde{u}}(b_{0};s_{0}),{\tilde{v}}(b_{0};s_{0}))^\top . \end{aligned}$$

On $x=x_{h}$ for $h\in {\mathbb {N}}$, the grid points are defined to be the intersections of line $x=x_{h}$ with the self-similar rays

$$\begin{aligned} y=\left( b_{0}+n\Delta \sigma \right) x\qquad \text{ for } n\in {\mathbb {Z}}. \end{aligned}$$

Here $\Delta \sigma >0$ is chosen so that $\Delta \sigma >\dfrac{4\Delta x}{x_{0}}\max _{i=1,2}\{|\lambda _i(G(s_{0}))|\}$, and hence the numerical grids satisfy the usual Courant-Friedrichs-Lewy condition. Then we define the approximate solution $U_{\Delta x,\vartheta }$ to be a piecewise smooth solution to the self-similar system (2.4), the approximate solution $U_{\Delta x,\vartheta }^b$ on the boundary, the approximate boundary $\Gamma _{\Delta x,\vartheta }=\big \{(x,y):\,y=b_{\Delta x,\vartheta }(x)\big \}$, and the numerical grids inductively in *h*, $h=0,1,2,\cdots$.

Suppose that the approximate solution has been defined on $x<x_{h}$. The grid points on $x=x_{h}$ are denoted by $y_n(h)$ for $n\in {\mathbb {Z}}$. Set that

$$\begin{aligned} r_{h,n}=y_{n}(h)+\vartheta _{h}\big (y_{n+1}(h)-y_{n}(h)\big )\qquad \,\, \text{ for } n\in {\mathbb {Z}}. \end{aligned}$$

Then the approximate solution $U_{\Delta x,\vartheta }(x_{h},y)$ for $y\in (y_{n}(h), y_{n+1}(h))$ is defined to be the solution $U_{self,\Delta x,\vartheta }(\sigma (x,y))$ of (2.3) with the self-similar variable $\sigma (x,y)=\frac{y}{x_{h}}$ and with the initial data

$$\begin{aligned} \sigma =\frac{r_{h,n}}{x_{h}}:\quad U_{self,\Delta x,\vartheta } =U_{\Delta x,\vartheta }(x_{h},r_{h,n})\triangleq U_{\Delta x,\vartheta }(x_{h}-,r_{h,n}+) \qquad \text{ for } n\in {\mathbb {Z}}. \end{aligned}$$

For the discontinuities at the grid points $(x_{h},y_{n}(h))$ for $n\in {\mathbb {Z}}$, we solve the Riemann problems for (2.1) with the Riemann data

3.1 $$\begin{aligned} U|_{x=x_{h}}=\left\{ \begin{aligned}&U_{\Delta x,\vartheta }(x_{h},y_{n}(h)-)\qquad \text{ for } y<y_{n}(h), \\&U_{\Delta x,\vartheta }(x_{h},y_{n}(h)+)\qquad \text{ for } y>y_{n}(h), \end{aligned} \right. \end{aligned}$$

and the solution consisting of rarefaction waves and shock waves has form $U_{Rie}(\eta )$ with $\eta =\dfrac{y-y_n(h)}{x-x_{h}}$. Setting $\sigma _{h,n+\frac{1}{2}}\triangleq \dfrac{1}{2x_{h}}\big (y_{n+1}(h+1)+y_{n}(h+1)\big )$ for $n\in {\mathbb {Z}}$, then, in the region

$$\begin{aligned} \Omega _{h+1,n}=\big \{(x,y)\, :\, x_{h}< x<x_{h+1}, \sigma _{h,n+\frac{1}{2}}>\sigma >\sigma _{h,n-\frac{1}{2}}\big \}, \end{aligned}$$

along the ray

$$\begin{aligned} \big \{(x,y)\,:\, \frac{y-y_n(h)}{x-x_{h}}=\eta ,\,x_{h}<x<x_{h+1}\big \}, \end{aligned}$$

the approximate solution $U_{\Delta x,\vartheta }(x,y)$ is defined to be the solution: $U_{self,\Delta x,\vartheta }(\sigma (x,y))$ of (2.3) with the self-similar variable $\sigma (x,y)=\frac{y}{x}$ and with the initial data

$$\begin{aligned} \sigma =\frac{y_{n}}{x_{h}}:\quad U_{self,\Delta x,\vartheta }=U_{Rie}(\eta ). \end{aligned}$$

The approximate boundary $\Gamma _{\Delta x,\vartheta }=\big \{(x,y):\,y=b_{\Delta x,\vartheta }(x)\big \}$ is traced continuously; see [10, 38, 48]. For $x\in (0,x_{0})$, let $b_{\Delta x,\vartheta }(x)=b_{0}x$. Suppose that the approximate solution is constructed for $x<x_{h}$ and that $y_{n_{b,h}}<b_{\Delta x,\vartheta }(x_{h}-)<y_{n_{b,h}+1}$. We call interval $y_{n_{b,h}-1}<y<y_{n_{b,h}+1}$ the boundary region at $x=x_{h}$. In this boundary region, we first solve the self-similar problem (2.3) with the initial data

$$\begin{aligned} \sigma =\frac{r_{h,n_{b}-1}}{x_{h}}:\quad U_{self}=U_{\Delta x,\vartheta }(x_{h}-,r_{h,n_{b}-1}+), \end{aligned}$$

and with the self-similar variable $\sigma (x_{h},y)=\frac{y}{x_{h}}$. We denote the solution by $U_{self}(\sigma (x_{h},y))$. Given $p^{b}_{\Delta x,h+1}$, by Proposition 2.2, there is $\beta _{1}$ such that

$$\begin{aligned} \frac{1}{2}\big |\Phi (\beta _{1},0;U_{self}(\sigma (x_{h},b_{\Delta x,\vartheta }(x_{h}))))\big |^2 +\frac{\gamma }{\gamma -1}(p^{b}_{\Delta x,h+1})^{\frac{\gamma -1}{\gamma }} =\frac{1}{2}+\frac{\gamma }{\gamma -1}p_{\infty }^{\frac{\gamma -1}{\gamma }}. \end{aligned}$$

Then we define

$$\begin{aligned} U_{\Delta x,\vartheta }^b(x_{h})\triangleq \Phi (\beta _{1},0;U_{self}(\sigma (x_{h},b_{\Delta x,\vartheta }(x_{h})))), \end{aligned}$$

and

3.2 $$\begin{aligned} b_{\Delta x,\vartheta }(x) =b_{\Delta x,\vartheta }(x_{h}-)+\frac{v^b_{\Delta x,\vartheta }(x_{h})}{u^b_{\Delta x,\vartheta }(x_{h})}\,(x-x_{h}) \qquad \, \text{ for } x\in [x_{h},x_{h+1}). \end{aligned}$$

Next, solve again the self-similar problem (2.3) with initial data

$$\begin{aligned}U_{-}(\sigma (x_{h},b_{\Delta x,\vartheta }(x_{h})))=U_{\Delta x,\vartheta }^b(x_{h})\end{aligned}$$

and with the self-similar variable $\sigma (x_{h},y)=\frac{y}{x_{h}}$. Denote the solution by $U_{-}(\sigma (x_{h},y))$. We define the approximate solution in the boundary region as

$$\begin{aligned} U_{\Delta x,\vartheta }(x_{h},y)=U_{-}(\sigma (x_{h},y))\qquad \, \text{ for } x_{h}\leqq x<x_{h+1}. \end{aligned}$$

The discontinuities at $(x_{h},y_{n_{b,h}-1})$ are resolved by the same methods as before.

The leading strong conical shock $S_{\Delta x,\vartheta }=\big \{(x,y):\,y=\chi _{\Delta x,\vartheta }(x)\big \}$ next to the uniform upstream flow is also traced continuously; see [10, 38, 48]. For $x\in (0,x_{0})$, let $\chi _{\Delta x,\vartheta }(x)=s_{0}x$. Suppose that the approximate solution is constructed for $x<x_{h}$ and that $y_{n_{\chi ,h}-1}<\chi _{\Delta x,\vartheta }(x_{h}-)<y_{n_{\chi ,h}}$. We call interval $y_{n_{\chi ,h}-1}<y<y_{n_{\chi ,h}+1}$ the front region at $x=x_{h}$. In this front region, we first solve the self-similar problem (2.3) with the initial data

$$\begin{aligned} \sigma =\frac{r_{h,n_{\chi }}}{x_{h}}:\quad U_{self}=U_{\Delta x,\vartheta }(x_{h}-,r_{h,n_{\chi }}+), \end{aligned}$$

and with the self-similar variable $\sigma (x_{h},y)=\frac{y}{x_{h}}$. Denote the solution by $U_{self}(\sigma (x_{h},y))$. Then we solve the Riemann problem (2.1) with the initial data

3.3 $$\begin{aligned} U(x_{h},y)=\left\{ \begin{aligned}&U_{\infty },\,\,\,  &   y<\chi _{\Delta x,\vartheta }(x_{h}-), \\&U_{self}(\sigma (x_{h},\chi _{\Delta x,\vartheta }(x_{h}))), \,\,\,  &   \chi _{\Delta x,\vartheta }(x_{h}-)<y<y_{n_{\chi ,h}+1}. \end{aligned} \right. \end{aligned}$$

The solution *U*(*x*, *y*) contains a weak 2-wave $\beta _{2}$ and a relatively strong 1-shock wave $s_{\Delta x,\vartheta }(h+1)$ such that

$$\begin{aligned} U_{self}(\sigma (x_{h},\chi _{\Delta x,\vartheta }(x_{h})))=\Phi (0,\beta _{2};G(s_{\Delta x,\vartheta }(h+1);U_{\infty })). \end{aligned}$$

Then we define that

3.4 $$\begin{aligned} \chi _{\Delta x,\vartheta }(x)=\chi _{\Delta x,\vartheta }(x_{h}-)+s_{\Delta x,\vartheta }(h+1)(x-x_{h}) \qquad \text{ for } x\in [x_{h},x_{h+1}). \end{aligned}$$

Next, solve again the self-similar problem (2.3) with initial data $U_{+}(\sigma (x_{h},\chi _{\Delta x,\vartheta }(x_{h})))=G(s_{\Delta x,\vartheta }(h+1);U_{\infty })$ and with the self-similar variable $\sigma (x_{h},y)=\frac{y}{x_{h}}$. Denote the solution by $U_{+}(\sigma (x_{h},y))$. We define the approximate solution in the front region as

$$\begin{aligned} U_{\Delta x,\vartheta }(x_h,y)=\left\{ \begin{aligned}&U_{\infty },\,\,\,  &   y<\chi _{\Delta x,\vartheta }(x_{h}), \\&U_{+}(\sigma (x_{h},y)),\,\,\,  &   \chi _{\Delta x,\vartheta }(x_{h})<y<y_{n_{\chi ,h}+1}. \end{aligned} \right. \end{aligned}$$

The discontinuities at $(x_{h},y_{n_{\chi ,h}})$ are resolved by the same methods as before.

## Riemann-Type Problems and Interaction Estimates

Let $\Omega _{\Delta x,\vartheta ,h}=\{(x,y)\,:\,y<b_{\Delta x,\vartheta }, x_{h-1}\leqq x< x_{h}\}$ and $h\in {\mathbb {N}}_{+}$. In order to define the approximate solutions in $\Omega _{\Delta x,\vartheta }\triangleq \bigcup _{k=0}^\infty \Omega _{\Delta x,\vartheta ,k}$, the approximate boundary $\Gamma _{\Delta x,\vartheta }\triangleq \bigcup _{k=0}^\infty \Gamma _{\Delta x,\vartheta ,k}$, and the approximate leading shock $S_{\Delta x,\vartheta }\triangleq \bigcup _{k=0}^\infty S_{\Delta x,\vartheta ,k}$, we need a uniform bound of them to ensure that all the Riemann problems and the differential equations (2.3) are solvable. To achieve this, the following formulas are used:

(i) If $$f\in \text {C}^1({\mathbb {R}})$$, then
  4.1 $$\begin{aligned} f(t)-f(0)=t\int _{0}^1f_t(\mu t){\textrm{d}}\mu \qquad \,\, \text{ for } t\in {\mathbb {R}}. \end{aligned}$$
(ii) If $f\in \text {C}^2({\mathbb {R}})$, then
  4.2 $$\begin{aligned} \begin{aligned}&f(s,t)-f(s,0)-f(0,t)+f(0,0)\\&\quad = st\int _{0}^1\int _{0}^1f_{st}(\mu s,\lambda t){\textrm{d}}\mu {\textrm{d}}\lambda \qquad \,\, \text{ for } (s,t)\in {\mathbb {R}}^2. \end{aligned} \end{aligned}$$

From now on, we use Greek letters $\alpha$, $\beta$, $\nu$, and $\delta$ to denote the elementary waves in the approximate solution, and $\alpha _{i}$, $\beta _{i}$, $\nu _{i}$, and $\delta _{i}$ stand for the corresponding *i*-th components for $i=1,2$. As in [14, 44, 48, 52], a curve *I* is called a mesh curve provided that *I* is a space-like curve and consists of the line segments joining the random points one by one in turn. *I* divides region $\Omega _{\Delta x, \vartheta }$ into two parts: $I^-$ and $I^+$, where $I^-$ denotes the part containing line $x=x_{0}$. For any two mesh curves *I* and *J*, we use $J>I$ to represent that every mesh point of curve *J* is either on *I* or contained in $I^+$. We say *J* is an immediate successor to *I* if $J>I$ and every mesh point of *J* except one is on *I* in general but three when these points are near the approximate boundary or the approximate shock.

Assume now that $U_{\Delta x,\vartheta }$ has been defined in $\bigcup _{k=0}^h\Omega _{\Delta x,\vartheta ,k}$ and the following conditions are satisfied:

- $\mathbf {{\text {H}}_{1}\text{(h) }}$: $\{S_{\Delta x,\vartheta ,k}\}_{k=0}^{h}$ forms an approximate strong shock $S_{\Delta x,\vartheta }|_{0\leqq x<x_{h}}$, and $\{\Gamma _{\Delta x,\vartheta ,k}\}_{k=0}^{h}$ forms an approximate boundary $\Gamma _{\Delta x,\vartheta }|_{0\leqq x<x_{h}}$, both of which emanate from the origin;
- $\mathbf {{\text {H}}_{2}\text{(h) }}$: In each $\Omega _{\Delta x,\vartheta ,k}$ for $0\leqq k\leqq h$, the strong 1-shock $S_{\Delta x, \vartheta , k}$ divides $\Omega _{\Delta x,\vartheta ,k}$ into two parts: $\Omega _{\Delta x,\vartheta ,k}^-$ and $\Omega _{\Delta x,\vartheta ,k}^+$, where $\Omega _{\Delta x,\vartheta ,k}^+$ is the part between $S_{\Delta x, \vartheta , k}$ and $\Gamma _{\Delta x, \vartheta , k}$;
- $$\mathbf {{\text {H}}_{3}\text{(h) }}$$: $$U_{\Delta x,\vartheta }|_{\Omega _{\Delta x,\vartheta ,k}^-}=U_{\infty }$$, $$\,\,U_{\Delta x,\vartheta }|_{\Omega _{\Delta x,\vartheta ,k}^+} \in O_{\varepsilon _{0}}(G(s_{0}))\cap O_{\varepsilon _{0}}(\mathbb {W}(p_{0},p_{\infty }))$$, and
  $$\begin{aligned} U_{\Delta x,\vartheta }(x,b_{\Delta x, \vartheta }(x)-)=U_{\Delta x,\vartheta , k}^{b} \in O_{\varepsilon _{0}}(G(s_{0}))\cap O_{\varepsilon _{0}}( \mathbb {W}(p_{0},p_{\infty })) \end{aligned}$$
  for $x_{k}\leqq x<x_{k+1}$, $0\leqq k\leqq h$, and $0<\varepsilon _{0}<\min \{\varepsilon _{j},j=1,2,3\}$, where $\varepsilon _{j}$ are introduced in Propositions 2.1–2.3 for $j=1,2,3$.

Then we prove that $U_{\Delta x,\vartheta }$ can be defined in $\Omega _{\Delta x,\vartheta ,h+1}$ satisfying conditions ${\textrm{H}}_{1}(\hbox {h}+1)$–${\textrm{H}}_3(\hbox {h}+1)$. As in [24] (see also [10, 14, 44]), we consider a pair of the mesh curves (*I*, *J*) lying in $\{x_{h-1}<x<x_{h+1}\}\cap \Omega _{\Delta x, \vartheta }$ with *J* being an immediate successor of *I*.

Now, let $\Lambda$ be the region between *I* and *J*, and let

$$\begin{aligned} U_{\Delta x,\vartheta }\in O_{\varepsilon _{0}}(G(s_{0}))\cap O_{\varepsilon _{0}}(\mathbb {W}(p_{0},p_{\infty })). \end{aligned}$$

### Case 1

$\Lambda$ *is between* $\Gamma _{\Delta x,\vartheta }$ *and* $S_{\Delta x, \vartheta }$.  In this case, we consider the interactions between weak waves. From the construction of the approximate solutions, the waves entering $\Lambda$ issuing from $(x_{h-1},y_{n-1}(h-1))$ and from $(x_{h-1},y_{n}(h-1))$ are denoted by $\alpha =(\alpha _{1},\alpha _{2})$ and $\beta =(\beta _{1},\beta _{2})$, respectively. We denote that

$$\begin{aligned} \sigma _{0}&=\frac{r_{h-1,n}}{x_{h-1}},\&{\bar{\sigma }}_{0}&=\frac{r_{h-1,n-1}}{x_{h-1}},\&{\hat{\sigma }}_{0}&=\frac{r_{h-1,n-2}}{x_{h-1}},\\ \sigma _{1}&=\frac{y_{n}(h-1)}{x_{h-1}}=\frac{y_{n}(h)}{x_{h}},\&\sigma _{2}&=\frac{y_{n-1}(h-1)}{x_{h-1}}=\frac{y_{n-1}(h)}{x_{h}}, \end{aligned}$$

and

$$\begin{aligned} \begin{aligned} U_{1}&=U_{\Delta x,\vartheta }(x_{h-1}-,r_{h-1,n}+),&U_{2}&=U_{\Delta x,\vartheta }(x_{h-1}-,r_{h-1,n-1}+),\\ U_3&=U_{\Delta x,\vartheta }(x_{h-1}-,r_{h-1,n-2}+).&\end{aligned} \end{aligned}$$

**Fig. 4:**
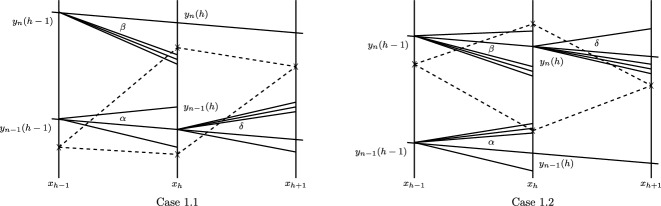
Interaction between weak waves

### Case 1.1

Let $\delta =(\delta _{1},\delta _{2})$ be the waves issuing from $(x_{h},y_{n-1}(h))$; see Fig. 4. Then we need to solve the following equations of $\delta =(\delta _{1},\delta _{2})$:

4.3 $$\begin{aligned} {\tilde{U}}(\sigma _{1};\sigma _{2},\Phi (\delta _{1},\delta _{2};U_{l})) =\Phi (\beta _{1},0;{\tilde{U}}(\sigma _{1};\sigma _{2},\Phi (\alpha _{1},\alpha _{2};U_{l}))), \end{aligned}$$

where $$U_{l}={\tilde{U}}(\sigma _{2};{\hat{\sigma }}_{0},U_3)$$.

### Lemma 4.1

Equation (4.3) has a unique solution $\delta =(\delta _{1},\delta _{2})$ such that

$$\begin{aligned} \delta _{1}=\alpha _{1}+\beta _{1}+O(1)Q(\Lambda ),\qquad \delta _{2}=\alpha _{2}+O(1)Q(\Lambda ), \end{aligned}$$

where

$$\begin{aligned} Q(\Lambda )=Q^0(\Lambda )+Q^1(\Lambda ) \end{aligned}$$

with

$$\begin{aligned} Q^0(\Lambda )=\sum \big \{|\alpha _{j}||\beta _k|\,:\, \alpha _{j}\text { and }\beta _k\text { approach}\big \},\qquad Q^1(\Lambda )=|\beta _{1}||\Delta \sigma |, \end{aligned}$$

and $\Delta \sigma =\sigma _{1}-\sigma _{2}$, and *O*(1) depends continuously on $M_{\infty }$ but independent of $(\alpha , \beta , \Delta \sigma )$.

### Proof

Lemma 2.10 yields

$$\begin{aligned}&\lim _{M_{\infty }\rightarrow \infty } \det \bigg (\left. \frac{\partial \Phi (\delta _{1},\delta _{2};U_{l})}{\partial (\delta _{1},\delta _{2})}\right| _{\{\delta _{1}=\delta _{2}=0,\, U_{l}\in \mathbb {W}(p_{0,p_{\infty }})\}}\bigg )\\&\quad =\dfrac{4\cos ^2(\theta _{0}+\theta _{m}^0)\cos ^2(\theta _{0}-\theta _{m}^0)\cos ^2\theta _{0}\cos ^2\theta ^0_{m}\sin (2\theta _{m}^0)}{(\gamma +1)^2}. \end{aligned}$$

Then, by the implicit function theorem, system (4.3) has a unique $\hbox {C}^2$–solution:

$$\begin{aligned} \delta =\delta (\alpha ,\beta ,\Delta \sigma ;U_{l}) \end{aligned}$$

in a neighborhood of $(\alpha ,\beta ,\Delta \sigma ,U_{l})=(0,0,0,G(s_0))$. Due to (4.2), we have

$$\begin{aligned} \delta _{i}(\alpha ,\beta ,\Delta \sigma ;U_{l})&=\delta _{i}(\alpha ,0,\Delta \sigma ;U_{l})\\  &\quad +\delta _{i}(\alpha ,\beta ,0;U_{l}) -\delta _{i}(\alpha ,0,0;U_{l})+O(1)|\beta ||\Delta \sigma |\\&=\alpha _{i}+\beta _{i}+O(1)Q^0(\Lambda )+O(1)|\beta ||\Delta \sigma | \qquad \text{ for } i=1,2, \end{aligned}$$

where $\beta _{2}=0$. Then the proof is complete. $\square$

### Case 1.2

Let $\delta =(\delta _{1},\delta _{2})$ be the waves issuing from $(x_{h},y_{n}(h))$; see Fig. 4. Then we need to solve the following equations of $\delta =(\delta _{1},\delta _{2})$:

4.4 $$\begin{aligned} \Phi (\delta _{1},\delta _{2};{\tilde{U}}(\sigma _{1};\sigma _{2},U_{l})) =\Phi (\beta _{1},\beta _{2};{\tilde{U}}(\sigma _{1},\sigma _{2};\Phi (0,\alpha _{2};U_{l}))), \end{aligned}$$

where $U_{l}$ satisfies ${\tilde{U}}({\bar{\sigma }}_{0};\sigma _{1},\Phi (0,\alpha _{2};U_{l}))=U_{2}$. Similarly, we have

### Lemma 4.2

Equation (4.4) has a unique solution $\delta =(\delta _{1},\delta _{2})$ such that

$$\begin{aligned} \delta _{1}=\beta _{1}+O(1)Q(\Lambda ),\qquad \delta _{2}=\alpha _{2}+\beta _{2}+O(1)Q(\Lambda ), \end{aligned}$$

where

$$\begin{aligned} Q(\Lambda )=Q^0(\Lambda )+Q^1(\Lambda ) \end{aligned}$$

with

$$\begin{aligned} Q^0(\Lambda )=\sum \{|\alpha _{j}||\beta _k|\,:\,\alpha _{j}\text { and }\beta _k\text { approach}\},\qquad Q^1(\Lambda )=|\alpha ||\Delta \sigma |, \end{aligned}$$

and $\Delta \sigma =\sigma _{1}-\sigma _{2}$, and *O*(1) depends continuously on $M_{\infty }$ but independent of $(\alpha , \beta , \Delta \sigma )$.

**Fig. 5:**
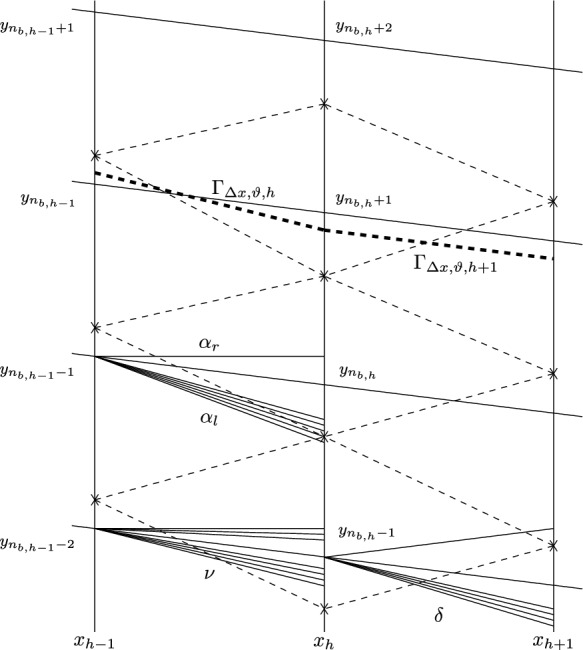
Reflection at the boundary

### Case 2

$\Lambda _b$ covers the part of $\Gamma _{\Delta x,\vartheta }$ but none of $S_{\Delta x, \vartheta }$. We take three diamonds at the same time, as shown in Fig. 5. Let $\Delta _{h,n_{b,h}-1}$, $\Delta _{h,n_{b,h}}$, and $\Delta _{h,n_{b,h}+1}$ denote the diamonds centering in $(x_{h},y_{n_{b,h}-1})$, $(x_{h},y_{n_{b,h}})$, and $(x_{h},y_{n_{b,h}+1})$, respectively, and denote $\Lambda _b=\Delta _{h,n_{b,h}-1}\cup \Delta _{h,n_{b,h}}\cup \Delta _{h,n_{b,h}+1}$. Let $\alpha$ and $\nu$ be the weak waves issuing from $(x_{h-1},y_{n_{b,h-1}-1})$ and $(x_{h-1},y_{n_{b,h-1}-2})$ respectively, and entering $\Lambda _b$. We divide $\alpha =(\alpha _{1},\alpha _{2})$ into parts $\alpha _{l}=(\alpha _{l,1},0)$ and $\alpha _{r}=(\alpha _{r,1},\alpha _{r,2})$, where $\alpha _{l}$ and $\alpha _{r}$ entering $\Delta _{h,n_{b,h}-1}$ and $\Delta _{h,n_{b,h}}$, respectively. Moreover, let $\nu =(\nu _{1},\nu _{2})$, and let $\delta$ be the outgoing wave issuing from $(x_{h},y_{n_{b,h}-1})$.

For simplicity of notation, we denote that

$$\begin{aligned}&\sigma _{\alpha }=\sigma (x_{h-1},y_{n_{b,h-1}-1}),  &   \sigma _{b}(h-1)=\sigma (x_{h-1},b_{\Delta x,\vartheta }(x_{h-1})),\\&\sigma _{b}(h)=\sigma (x_{h},b_{\Delta x,\vartheta }(x_{h})),  &   \sigma _{\nu }=\sigma (x_{h-1},y_{n_{b,h-1}-2}),\\&\sigma _{0}=\sigma (x_{h-1},r_{h-1,n_b-2}),  &   \Delta \sigma _{\alpha }=\sigma _{b}(h-1)-\sigma _{\alpha },\\&\Delta {\bar{\sigma }}_{\alpha }=\sigma _{b}(h)-\sigma _{\alpha },  &   \Delta \sigma _{b_{h}}=\sigma _{b}(h)-\sigma _{b}(h-1),\\&\Delta \sigma _{\nu }= \sigma _{\alpha }-\sigma _{\nu },  &   \end{aligned}$$

and $$U_{1}=U_{\Delta x,\vartheta }(x_{h-1}-,r_{h-1,n_b-2}+)$$. Let $$U_{l}=\Phi (\alpha _{l,1},0;{\tilde{U}}(\sigma _{\alpha };\sigma _{0},U_{1}))$$.

To gain the estimates of $\delta$, we need to deal with the equation

4.5 $$\begin{aligned} \begin{aligned}&\frac{1}{2}|\Phi (\beta _{1},0;{\tilde{U}}(\sigma _{b}(h);\sigma _{\alpha },U_{l}))|^2 +\frac{\gamma }{\gamma -1}(p^{b}_{\Delta x,h+1})^{\frac{\gamma -1}{\gamma }}\\&\quad = \frac{1}{2}|{\tilde{U}}(\sigma _{b}(h-1);\sigma _{\alpha },\Phi (\alpha _{r,1},\alpha _{r,2};U_{l}))|^2 +\frac{\gamma }{\gamma -1}(p^{b}_{\Delta x,h})^{\frac{\gamma -1}{\gamma }}, \end{aligned} \end{aligned}$$

and then we obtain the following lemma:

### Lemma 4.3

Equation (4.5) has a unique solution $\beta _{1}=\beta _{1}(\alpha _{r,1},\alpha _{r,2},\Delta \sigma _\alpha ,\Delta {\bar{\sigma }}_\alpha ,\omega _{h+1};U_{l})\in \text {C}^2$ in a neighborhood of $(\alpha _{r,1},\alpha _{r,2},\Delta \sigma _\alpha ,\Delta {\bar{\sigma }}_\alpha ,\omega _{h+1},U_{l})=(0,0,0,0,0,G(s_{0}))$ with $\omega _{h+1}=p^{b}_{\Delta x,h+1}-p^{b}_{\Delta x,h}$ such that

4.6 $$\begin{aligned} \begin{aligned} \delta _{1}&=\alpha _{r,1}+\alpha _{l,1}+\nu _{1} +K_{r,1}\alpha _{r,2} +K_{\sigma ,1}\Delta \sigma _{b_{h}}+K_{b,1}\omega _{h+1}+O(1)Q(\Lambda _b),\\ \delta _{2}&=\nu _{2}+K_{r,2}\alpha _{r,2} +K_{\sigma ,2}\Delta \sigma _{b_{h}}+K_{b,2}\omega _{h+1}+O(1)Q(\Lambda _b), \end{aligned} \end{aligned}$$

with

4.7 $$\begin{aligned} Q(\Lambda _b)=Q^0((\alpha _{1},0),\nu )+|\alpha _{1}||\Delta \sigma _\nu |+|\alpha _{r,1}||\Delta \sigma _\alpha |, \end{aligned}$$

where *O*(1) depends continuously on $M_{\infty }$. Moreover, when $\alpha _{r,1}=\alpha _{r,2}=\Delta \sigma _\alpha =\Delta {\bar{\sigma }}_\alpha =\omega _{h+1}=0$, $p^{b}_{\Delta x,h+1}=p_{0}$, and $U_{l}=G(s_{0})$,

4.8 $$\begin{aligned}&\lim _{M_{\infty }\rightarrow \infty }K_{r,1}=-\dfrac{\cos ^2(\theta _{0}+\theta _{m}^0)}{\cos ^2(\theta _{0}-\theta _{m}^0)},\ \lim _{M_{\infty }\rightarrow \infty }|K_{b,i}|<\infty ,\nonumber \\&\quad \lim _{M_{\infty }\rightarrow \infty }K_{r,2}=0,\qquad \lim _{M_{\infty }\rightarrow \infty }K_{\sigma ,i}=0, \end{aligned}$$

for $$i=1,2$$.

### Proof

A direct computation leads to

$$\begin{aligned} \left. \dfrac{1}{2} \frac{\partial (|\Phi (\beta _{1},0;{\tilde{U}}(\sigma _{b}(h);\sigma _{\alpha },U_{l}))|^2)}{\partial \beta _{1}}\right| _{\{\delta _{1}=\Delta {\bar{\sigma }}_\alpha =0, U_{l}=G(s_{0})\}}=r_{1}(G(s_{0}))\cdot G(s_{0}). \end{aligned}$$

Lemma 2.7, together with the implicit function theorem, implies that there is a unique $\text {C}^2-$solution

4.9 $$\begin{aligned} \beta _{1}=\beta _{1}(\alpha _{r,1},\alpha _{r,2},\Delta \sigma _\alpha ,\Delta {\bar{\sigma }}_\alpha ,\omega _{h+1};U_{l}) \end{aligned}$$

in a neighborhood of $(\alpha _{r,1},\alpha _{r,2},\Delta \sigma _\alpha ,\Delta {\bar{\sigma }}_\alpha ,\omega _{h+1},U_{l})=(0,0,0,0,0,G(s_{0}))$.

Using (4.1)–(4.2), we have

$$\begin{aligned} \beta _{1}&=\beta _{1}(\alpha _{r,1},\alpha _{r,2},\Delta \sigma _\alpha ,\Delta \sigma _\alpha ,\omega _{h+1};U_{l}) +{\bar{K}}_{\sigma ,1}(\Delta {\bar{\sigma }}_\alpha -\Delta \sigma _\alpha )\\&=\beta _{1}(\alpha _{r,1},0,\Delta \sigma _\alpha ,\Delta \sigma _\alpha ,0;U_{l}) +{\bar{K}}_{\sigma ,1}\Delta \sigma _{b_{h}}+{\bar{K}}_{r,1}\alpha _{r,2}+{\bar{K}}_{b,1}\omega _{h+1}\\&=\alpha _{r,1} +{\bar{K}}_{\sigma ,1}\Delta \sigma _{b_{h}}+{\bar{K}}_{r,1}\alpha _{r,2}+{\bar{K}}_{b,1}\omega _{h+1}+O(1)|\alpha _{r,1}||\Delta \sigma _\alpha |. \end{aligned}$$

Taking the derivative with respect to $\Delta \sigma _{b_{h}}$ in (4.5) at $(\alpha _{r,1},\alpha _{r,2},\Delta \sigma _\alpha ,\Delta {\bar{\sigma }}_\alpha ,\omega _{h+1},U_{l})=(0,0,0,0,0,G(s_{0}))$, we obtain

$$\begin{aligned} G(s_{0})\cdot r_{1}(G(s_{0}))\frac{\partial \beta _{1}}{\partial \Delta \sigma _{b_{h}}} +G(s_{0})\cdot \frac{\partial {\tilde{U}}(\sigma _\alpha +\Delta {\bar{\sigma }}_\alpha +\Delta \sigma _{b_{h}};\sigma _\alpha ,G(s_{0}))}{\partial \Delta \sigma _{b_{h}}}=0, \end{aligned}$$

which yields

$$\begin{aligned} \lim _{M_{\infty }\rightarrow \infty } \left. \frac{\partial \beta _{1}}{\partial \Delta \sigma _{b_{h}}}\right| _{\{\alpha _{r,1}=\alpha _{r,2}=\Delta \sigma _\alpha =\Delta {\bar{\sigma }}_\alpha =\omega _{h+1}=0,\,p^{b}_{\Delta x,h+1}=p_{0},\,U_{l}=G(s_{0})\}}=0. \end{aligned}$$

Similarly, we have

$$\begin{aligned} \begin{aligned}&\lim _{M_{\infty }\rightarrow \infty }\left. \frac{\partial \beta _{1}}{\partial \omega _{h+1}}\right| _{\{\alpha _{r,1}=\alpha _{r,2}=\Delta \sigma _\alpha =\Delta {\bar{\sigma }}_\alpha =\omega _{h+1}=0,\,p^{b}_{\Delta x,h+1}=p_{0},\,U_{l}=G(s_{0})\}}\\  &\qquad =\lim _{M_{\infty }\rightarrow \infty }\dfrac{-p_{0}^{-\frac{1}{\gamma }}}{r_{1}(G(s_{0}))\cdot G(s_{0})}>-\infty ,\\&\lim _{M_{\infty }\rightarrow \infty }\left. \frac{\partial \beta _{1}}{\partial \alpha _{r,2}}\right| _{\{\alpha _{r,1}=\alpha _{r,2}=\Delta \sigma _\alpha =\Delta {\bar{\sigma }}_\alpha =\omega _{h+1}=0,\,p^{b}_{\Delta x,h+1}=p_{0},\,U_{l}=G(s_{0})\}}\\  &\qquad =-\dfrac{\cos ^2(\theta _{0}+\theta _{m}^0)}{\cos ^2(\theta _{0}-\theta _{m}^0)}. \end{aligned} \end{aligned}$$

By the construction of the approximate solution, we have

$$\begin{aligned}&{\tilde{U}}(\sigma _{b}(h);\sigma _\nu ,\Phi (\delta _{1},\delta _{2};U_m))\\&\quad =\Phi (\beta _{1},0;{\tilde{U}}(\sigma _{b}(h);\sigma _{\alpha },\Phi (\alpha _{l,1},0;{\tilde{U}}(\sigma _\alpha ;\sigma _\nu ,\Phi (\nu _{1},\nu _{2};U_m))))) \end{aligned}$$

with $U_m=U_{\Delta x,\vartheta }(x_{h},y_{n_{b,h-1}-2}-)$. Then, a similar argument as to that in Case 1 gives (4.6)–(4.8). This completes the proof. $\square$

### Lemma 4.4

In Case 2, for $b'_{h}:=b_{\Delta x,\vartheta }'(x_{h}-)$ for $h\in {\mathbb {N}}_{+}$,

$$\begin{aligned} b'_{h+1}-b'_{h}=K_{c,2}\alpha _{r,2}+K_{c,\sigma }\Delta \sigma _{b_{h}}+O(1)\omega _{h+1}+O(1)|\alpha _{r,1}||\Delta \sigma _{\alpha }| \end{aligned}$$

with *O*(1) depending continuously on $p_{0}$ such that

$$\begin{aligned}&\lim _{M_{\infty }\rightarrow \infty }K_{c,\sigma }|_{\{\alpha _{r,2}=\alpha _{r,1}=\Delta \sigma _\alpha =\Delta {\bar{\sigma }}_\alpha =\omega _{h+1}=0, p^{b}_{\Delta x,h+1}=p_{0},U_{l}=G(s_{0})\}}=-1,\\&\lim _{M_{\infty }\rightarrow \infty }K_{c,2}|_{\{\alpha _{r,2}=\alpha _{r,1}=\Delta \sigma _\alpha =\Delta {\bar{\sigma }}_\alpha =\omega _{h+1}=0, p^{b}_{\Delta x,h+1}=p_{0},U_{l}=G(s_{0})\}}\\&\quad \,=-\frac{4}{\gamma +1}\,\frac{\cos ^2\theta ^0_m\cos ^2(\theta ^0_m+\theta _{0})}{\cos ^2\theta _{0}}. \end{aligned}$$

### Proof

 From (3.2), we have

$$\begin{aligned} b'_{h+1}-b'_{h}&=\frac{\Phi ^{(2)}(\beta _{1}(\alpha _{r,1},\alpha _{r,2},\Delta \sigma _\alpha ,\Delta {\bar{\sigma }}_\alpha ,\omega _{h+1};U_{l}),0; {\tilde{U}}(\sigma _{b_{h}};\sigma _{\alpha },U_{l}))}{\Phi ^{(1)}(\beta _{1}(\alpha _{r,1},\alpha _{r,2},\Delta \sigma _\alpha ,\Delta {\bar{\sigma }}_\alpha ,\omega _{h+1};U_{l}),0;{\tilde{U}}(\sigma _{b_{h}};\sigma _{\alpha },U_{l}))}\\&\quad -\frac{{\tilde{U}}^{(2)}(\sigma _{b_{h-1}};\sigma _{\alpha },\Phi (\alpha _{r,1},\alpha _{r,2};U_{l}))}{{\tilde{U}}^{(1)}(\sigma _{b_{h-1}};\sigma _{\alpha },\Phi (\alpha _{r,1},\alpha _{r,2};U_{l}))}. \end{aligned}$$

By (4.1)–(4.2), we obtain

$$\begin{aligned} \begin{aligned}&b'_{h+1}-b'_{h}\\  &\quad =\frac{\Phi ^{(2)}(\beta _{1}(\alpha _{r,1},0,\Delta \sigma _\alpha ,\Delta {\bar{\sigma }}_\alpha ,0;U_{l}),0;{\tilde{U}}(\sigma _{b_{h}};\sigma _{\alpha },U_{l}))}{\Phi ^{(1)}(\beta _{1}(\alpha _{r,1},0,\Delta \sigma _\alpha ,\Delta {\bar{\sigma }}_\alpha ,0;U_{l}),0;{\tilde{U}}(\sigma _{b_{h}};\sigma _{\alpha },U_{l}))}\\  &\qquad \, -\frac{{\tilde{U}}^{(2)}(\sigma _{b_{h-1}};\sigma _{\alpha },\Phi (\alpha _{r,1},0;U_{l}))}{{\tilde{U}}^{(1)}(\sigma _{b_{h-1}};\sigma _{\alpha },\Phi (\alpha _{r,1},0;U_{l}))}\\  &\quad \quad \, +K_{c,2}\alpha _{r,2}+O(1)\omega _{h+1}\\  &\quad =\frac{\Phi ^{(2)}(\beta _{1}(\alpha _{r,1},0,\Delta \sigma _\alpha ,\Delta \sigma _\alpha ,0;U_{l}),0;{\tilde{U}}(\sigma _{b_{h-1}};\sigma _{\alpha },U_{l}))}{\Phi ^{(1)}(\beta _{1}(\alpha _{r,1},0,\Delta \sigma _\alpha ,\Delta \sigma _\alpha ,0;U_{l}),0;{\tilde{U}}(\sigma _{b_{h-1}};\sigma _{\alpha },U_{l}))}\\  &\qquad \, -\frac{{\tilde{U}}^{(2)}(\sigma _{b_{h-1}};\sigma _{\alpha },\Phi (\alpha _{r,1},0;U_{l}))}{{\tilde{U}}^{(1)}(\sigma _{b_{h-1}};\sigma _{\alpha },\Phi (\alpha _{r,1},0;U_{l}))}\\  &\quad \quad \, +K_{c,\sigma }\Delta \sigma _{b_{h}}+K_{c,2}\alpha _{r,2}+O(1)\omega _{h+1}\\  &\quad =K_{c,\sigma }\Delta \sigma _{b_{h}}+K_{c,2}\alpha _{r,2}+O(1)\omega _{h+1}+O(1)|\alpha _{r,1}||\Delta \sigma _{\alpha }|. \end{aligned} \end{aligned}$$

By similar calculation in Lemma 4.3, we have

$$\begin{aligned}&\lim _{M_{\infty }\rightarrow \infty }K_{c,\sigma }|_{\{\alpha _{r,2}=\alpha _{r,1}=\Delta \sigma _\alpha =\Delta {\bar{\sigma }}_\alpha =\omega _{h+1}=0, \,p^{b}_{\Delta x,h+1}=p_{0},\,U_{l}=G(s_{0})\}}=-1,\\&\lim _{M_{\infty }\rightarrow \infty }K_{c,2}|_{\{\alpha _{r,2}=\alpha _{r,1}=\Delta \sigma _\alpha =\Delta {\bar{\sigma }}_\alpha =\omega _{h+1}=0, \,p^{b}_{\Delta x,h+1}=p_{0},\,U_{l}=G(s_{0})\}}\\&\quad =-\frac{4}{\gamma +1}\,\frac{\cos ^2\theta ^0_m\cos ^2(\theta ^0_m+\theta _{0})}{\cos ^2\theta _{0}}. \end{aligned}$$

Then the proof is complete. $\square$

### Lemma 4.5

 For $\Delta x$ sufficiently small,

$$\begin{aligned} |b'_{h}-\sigma _{b}(h-1)| \geqq 6|\Delta \sigma _{b_h}|, \end{aligned}$$

where $$\sigma _{b}(h)=\frac{b_{\Delta x,\vartheta }(x_{h})}{x_{h}}$$.

### Proof

Using the notation as in Case 2, we have

$$\begin{aligned} b'_{h}=\dfrac{b_{\Delta x,\vartheta }(x_{h})-b_{\Delta x,\vartheta }(x_{h-1})}{\Delta x}. \end{aligned}$$

Then a direct computation leads to

$$\begin{aligned} |b'_h-\sigma _{b}(h-1)|&=\left| \dfrac{b_{\Delta x,\vartheta }(x_{h})-b_{\Delta x,\vartheta }(x_{h-1})}{\Delta x}-\sigma _{b}(h-1)\right| \\&=\left| \dfrac{\sigma _{b}(h)x_{h}-\sigma _{b}(h-1)x_{h-1}}{\Delta x}-\sigma _{b}(h-1)\right| \\&=\left| \dfrac{x_{h}}{\Delta x}\right| |\sigma _{b}(h)-\sigma _{b}(h-1)|\\&\geqq 6\left| \sigma _{b}(h)-\sigma _{b}(h-1)\right| \end{aligned}$$

for $\Delta x$ small enough. $\square$

Denote $\theta _{b}(h)=|\sigma _{b}(h-1)-b'_{h}|$ that measures the angle between boundary $\Gamma _{\Delta x,\vartheta ,h}$ and the ray issuing from the origin and passing through $(x_{h-1},b_{\Delta x,\vartheta }(x_{h-1}))$. Then we have the following estimate for $\theta _{b}(h)$:

### Lemma 4.6

 For $M_{\infty }$ sufficiently large and $\Delta x$ sufficiently small,

$$\begin{aligned} \theta _{b}(h)-\theta _{b}(h+1)\geqq |\Delta \sigma |-|K_{c,2}||\alpha _{r,2}|-C|\omega _{h+1}|-C|\alpha _{r,1}||\Delta \sigma _{\alpha }|, \end{aligned}$$

where $h\in {\mathbb {N}}_{+}$, and constant $C>0$ is independent of $M_{\infty }$ and $\Delta x$.

### Proof

We consider the following two different cases:

1. $$\sigma _{b}(h-1)<b'_{h}$$ so that $$\sigma _{b}(h)>\sigma _{b}(h-1)$$.

 

- If $b'_{h+1}>\sigma _{b}(h)$, then it follows from Lemma 4.4 that
  $$\begin{aligned}&\theta _{b}(h)-\theta _{b}(h+1)\\&\quad = b'_{h}-\sigma _{b}(h-1)-\big (b'_{h+1}-\sigma _{b}(h)\big )\\&\quad = (1-K_{c,\sigma })\Delta \sigma _{b_{h}}-K_{c,2}\alpha _{r,2}+O(1)\omega _{h+1}+O(1)|\alpha _{r,1}||\Delta \sigma _{\alpha }|\\&\quad \geqq |\Delta \sigma _{b_{h}}|-|K_{c,2}||\alpha _{r,2}|-C|\omega _{h+1}|-C|\alpha _{r,1}||\Delta \sigma _{\alpha }|. \end{aligned}$$
- If $b'_{h+1}<\sigma _{b}(h)$, then, from Lemma 4.4–4.5, we have
  $$\begin{aligned}&\theta _{b}(h)-\theta _{b}(h+1)\\&\quad = b'_{h}-\sigma _{b}(h-1)-\big (\sigma _{b}(h)-b'_{h+1}\big )\\&\quad = 2\big (b'_{h}-\sigma _{b}(h-1)\big )+b'_{h+1}\\&\qquad -\sigma _{b}(h)-\big (b'_{h}-\sigma _{b}(h-1)\big )\\&\quad \geqq (11+K_{c,\sigma })|\Delta \sigma _{b_{h}}|+K_{c,2}\alpha _{r,2}+O(1)\omega _{h+1}+O(1)|\alpha _{r,1}||\Delta \sigma _{\alpha }|\\&\quad \geqq |\Delta \sigma _{b_{h}}|-|K_{c,2}||\alpha _{r,2}|-C|\omega _{h+1}|-C|\alpha _{r,1}||\Delta \sigma _{\alpha }|. \end{aligned}$$

2. $$\sigma _{b}(h-1)>b'_{h}$$ so that $$\sigma _{b}(h)<\sigma _{b}(h-1)$$.

 

- If $b'_{h+1}>\sigma _{b}(h)$, then it follows from Lemma 4.4–4.5 that
  $$\begin{aligned}&\theta _{b}(h)-\theta _{b}(h+1)\\&\quad =\sigma _{b}(h-1)-b'_{h}-\big (b'_{h+1}-\sigma _{b}(h)\big )\\&\quad = 2(\sigma _{b}(h-1)-b'_{h})+\sigma _{b}(h)-b'_{h+1}-(\sigma _{b}(h-1)-b'_{h})\\&\quad \geqq (11+K_{c,\sigma })|\Delta \sigma _{b_{h}}|-K_{c,2}\alpha _{r,2}-O(1)\omega _{h+1}-O(1)|\alpha _{r,1}||\Delta \sigma _{\alpha }|\\&\quad \geqq |\Delta \sigma _{b_{h}}|-|K_{c,2}||\alpha _{r,2}|-C|\omega _{h+1}|-C|\alpha _{r,1}||\Delta \sigma _{\alpha }|. \end{aligned}$$
- If $b'_{h+1}<\sigma _{b}(h)$, then, from Lemma 4.4, we have
  $$\begin{aligned}&\theta _{b}(h)-\theta _{b}(h+1)\\&\quad =\sigma _{b}(h-1)-b'_{h}-\big (\sigma _{b}(h)-b'_{h+1}\big )\\&\quad =(-1+K_{c,\sigma })\Delta \sigma _{b_{h}}+K_{c,2}\alpha _{r,2}+O(1)\omega _{h+1}+O(1)|\alpha _{r,1}||\Delta \sigma _{\alpha }|\\&\quad \geqq |\Delta \sigma _{b_{h}}|-|K_{c,2}||\alpha _{r,2}|-C|\omega _{h+1}| -C|\alpha _{r,1}||\Delta \sigma _{\alpha }|. \end{aligned}$$

Note that we have used the fact that

$$\begin{aligned} \lim _{M_{\infty }\rightarrow \infty }K_{c,\sigma }|_{\{\alpha _{r,2}=\beta _{1}=\Delta \sigma _\alpha =\Delta {\bar{\sigma }}_\alpha =\omega _{h+1}=0, p^{b}_{\Delta x,h+1}=p_{0},U_{l}=G(s_{0})\}}=-1 \end{aligned}$$

in the above estimates. This completes the proof. $\square$

**Fig. 6:**
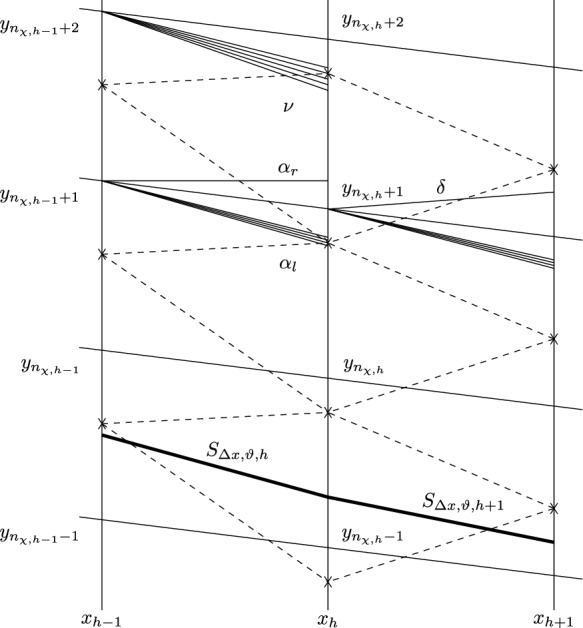
Near the strong shock wave

### Case 3

$\Lambda _{s}$ covers the part of $S_{\Delta x,\vartheta }$ but none of $\Gamma _{\Delta x, \vartheta }$. We take three diamonds at the same time, as shown in Fig. 6. Let $\Delta _{h,n_{\chi ,h}-1}$, $\Delta _{h,n_{\chi ,h}}$, and $\Delta _{h,n_{\chi ,h}+1}$ be the diamonds centering in $(x_{h},y_{n_{\chi ,h}-1})$, $(x_{h},y_{n_{\chi ,h}})$, and $(x_{h},y_{n_{\chi ,h}+1})$, respectively. Denote $\Lambda _{s}=\Delta _{h,n_{\chi ,h}-1}\cup \Delta _{h,n_{\chi ,h}}\cup \Delta _{h,n_{\chi ,h}+1}$. Let $\alpha$ and $\nu$ be the weak waves issuing from $(x_{h-1},y_{n_{\chi ,h-1}+1})$ and $(x_{h-1},y_{n_{\chi ,h-1}+2})$ respectively and entering $\Lambda _{s}$. We divide $\alpha$ into parts $\alpha _{l}=(\alpha _{l,1},0)$ and $\alpha _{r}=(\alpha _{r,1},\alpha _{r,2})$ where $\alpha _{l}$ and $\alpha _{r}$ enter $\Delta _{h,n_{\chi ,h}}$ and $\Delta _{h,n_{\chi ,h}+1}$, respectively. Moreover, let $\nu =(\nu _{1},0)$, and let $\delta$ be the outgoing wave issuing from $(x_{h},y_{n_{\chi ,h}+1})$.

Then, for simplicity of notation, we denote that

$$\begin{aligned}&\sigma _{\alpha }=\sigma (x_{h-1},y_{n_{\chi ,h-1}+1}),  &   \sigma _{\chi }(h-1)=\sigma (x_{h-1},\chi _{\Delta x,\vartheta }(x_{h-1})),\\&\sigma _{\chi }(h)=\sigma (x_{h},\chi _{\Delta x,\vartheta }(x_{h})),  &   \sigma _{\nu }=\sigma (x_{h-1},y_{n_{\chi ,h-1}+2}),\\&\Delta \sigma _{\alpha }=\sigma _{\alpha }-\sigma _{\chi }(h-1),  &   \Delta {\bar{\sigma }}_{\alpha }=\sigma _{\alpha }-\sigma _{\chi }(h),\\&\Delta \sigma _{\chi _{h}}=\sigma _{\chi }(h)-\sigma _{\chi }(h-1),  &   \Delta \sigma _{\nu }= \sigma _{\nu }-\sigma _{\alpha }.&\end{aligned}$$

To gain the estimates of $(s_{h+1},\delta )$, we need to deal with the equation:

4.10 $$\begin{aligned} {\tilde{U}}(\sigma _\alpha ;\sigma _{\chi }(h),\Phi (0,\beta _{2};G(s_{h+1};U_{\infty }))) =\Phi (\alpha _{l,1},0;{\tilde{U}}(\sigma _\alpha ;\sigma _{\chi }(h-1),G(s_{h};U_{\infty }))), \end{aligned}$$

to obtain the following lemma:

### Lemma 4.7

 Equation (4.10) has a unique solution $(s_{h+1},\beta _{2})$ in a neighborhood of

$$\begin{aligned} (\alpha _{l,1},\alpha _{r},\nu ,\Delta \sigma _{\alpha },\Delta \sigma _{\chi _{h}},s_{h})=(0,0,0,0,0,s_{0}) \end{aligned}$$

such that

4.11 $$\begin{aligned} \begin{aligned}&\delta _{1}=\alpha _{r,1}+\nu _{1}+\mu _{w,1}\Delta \sigma _{\chi _{h}}+K_{w,1}\alpha _{l,1}+O(1)Q(\Lambda _{s}),\\&\delta _{2}=\alpha _{r,2}+\mu _{w,2}\Delta \sigma _{\chi _{h}}+K_{w,2}\alpha _{l,1}+O(1)Q(\Lambda _{s}),\\&s_{h+1}= s_{h}+K_{s}\alpha _{l,1}+\mu _{s}\Delta \sigma _{\chi _{h}}, \end{aligned} \end{aligned}$$

with

4.12 $$\begin{aligned} Q(\Lambda _{s})=|\nu _{1}||\Delta \sigma _{\nu }|+Q^0(\alpha _{r},\nu ), \end{aligned}$$

where *O*(1) depends continuously on $M_{\infty }$. In addition, for $\alpha _{l}=0$, $\Delta \sigma _{\alpha }=\Delta \sigma _{\chi _{h}}=0$, and $s_{h}=s_{0}$, denoting the derivative of *G* by $G_{s}$, then

4.13 $$\begin{aligned} \begin{aligned}&\lim _{M_{\infty }\rightarrow \infty }K_{w,1}=0,  &   K_{w,2}=\dfrac{\det (r_1(G(s_0)),\,G_{s}(s_0))}{\det (r_2(G(s_0)),\,G_{s}(s_0))},\\&K_s=\dfrac{\det (r_2(G(s_0)),r_1(G(s_0)))}{\det (r_2(G(s_0)),G_{s}(s_0))},  &   \qquad \qquad \qquad \lim _{M_{\infty }\rightarrow \infty }\mu _s\in (-1,0),\\&\lim _{M_{\infty }\rightarrow \infty }\mu _{w,1}=0,  &   \mu _{w,2}=\dfrac{\det (\partial {\tilde{U}}/\partial (\Delta \sigma _{\chi _h}),\,G_{s}(s_0))}{\det (r_2(G(s_0)),\,G_{s}(s_0))}. \end{aligned} \end{aligned}$$

### Proof

From Lemma 2.10 and the implicit function theorem, (4.10) has a unique $\text {C}^2$–solution $(s_{h+1},\beta _{2})$ such that

$$\begin{aligned}&s_{h+1}=s_{h+1}(\alpha _{l,1},\Delta \sigma _{\alpha },\Delta {\bar{\sigma }}_{\alpha },\Delta \sigma _{\chi _{h}},s_{h}),\\&\beta _{2}=\beta _{2}(\alpha _{l,1},\Delta \sigma _{\alpha },\Delta {\bar{\sigma }}_{\alpha },\Delta \sigma _{\chi _{h}},s_{h}). \end{aligned}$$

A direct computation leads to

$$\begin{aligned} \beta _{2}&=\beta _{2}(\alpha _{l,1},\Delta \sigma _{\alpha },\Delta {\bar{\sigma }}_{\alpha },\Delta \sigma _{\chi _{h}},s_{h})\\&=\mu _{w,2}\Delta \sigma _{\chi _{h}}+K_{w,2}\alpha _{l,1} +\beta _{2}(0,\Delta \sigma _{\alpha },\Delta \sigma _{\alpha },0,s_{h})\\&=\mu _{w,2}\Delta \sigma _{\chi _{h}}+K_{w,2}\alpha _{l,1}. \end{aligned}$$

Similarly, we have

$$\begin{aligned} s_{h+1}=s_{h+1}(\alpha _{l,1},\Delta \sigma _{\alpha },\Delta {\bar{\sigma }}_{\alpha },\Delta \sigma _{\chi _{h}},s_{h}) =\mu _s\Delta \sigma _{\chi _{h}}+K_{s}\alpha _{l,1}+s_{h}. \end{aligned}$$

Next, we compute the coefficients: $K_s$, $K_{w,2}$, $\mu _{w,2}$, and $\mu _s$. Differentiating equation (4.10) with respect to $\alpha _{l,1}$ and $\Delta \sigma _{\chi _{h}}$, and then letting $\alpha _{l,1}=\Delta \sigma _{\alpha }=\Delta \sigma _{\chi _{h}}=0$ and $s_{h}=s_{0}$, we can obtain

$$\begin{aligned}&r_{2}(G(s_{0}))K_{w,2}+G_s(s_{0})K_s=r_{1}(G(s_{0})),\\&r_{2}(G(s_{0}))\mu _{w,2}+G_s(s_{0})\mu _s =\dfrac{\partial {\tilde{U}}}{\partial (\Delta \sigma _{\chi _{h}})}(\sigma _{\chi }(h);\sigma _{\chi }(h),G(s_{0})). \end{aligned}$$

Then Cramer’s rule gives the result. Moreover, since $\theta _{0}<0<\theta ^0_{ma}$ and $\theta _{0}\pm \theta ^0_{ma}\in (-\frac{\pi }{2},\frac{\pi }{2})$,

$$\begin{aligned} \lim _{M_{\infty }\rightarrow \infty }\mu _s=\frac{\cos \theta _{0}\sin \theta _{m}^0}{\sin (\theta _{0}-\theta _{m}^0)}\in (-1,0). \end{aligned}$$

By the construction of the approximate solution, we have

$$\begin{aligned}&{\tilde{U}}(\sigma _{\nu };\sigma _{\alpha },\Phi (\delta _{1},\delta _{2};{\tilde{U}}(\sigma _\alpha ;\sigma _{\chi }(h),U_m)))\\&\quad =\Phi (\nu _{1},0; {\tilde{U}}(\sigma _{\nu };\sigma _{\alpha }, \Phi (\alpha _{r,1},\alpha _{r,2}; {\tilde{U}}(\sigma _\alpha ;\sigma _{\chi }(h), \Phi (0,\beta _{2}(\alpha _{l,1},\Delta \sigma _{\alpha },\Delta {\bar{\sigma }}_{\alpha },\Delta \sigma _{\chi _{h}},s_{h});U_m))))), \end{aligned}$$

with

$$\begin{aligned} U_m=G(s_{h+1};U_{\infty }). \end{aligned}$$

Then, by similar arguments as to those in Case 1, we obtain

$$\begin{aligned} \delta _{1}&=\alpha _{r,1}+\nu _{1}+\mu _{w,1}\Delta \sigma _{\chi _{h}}+K_{w,1}\alpha _{l,1}+O(1)|\nu _{1}||\Delta \sigma _{\nu }|+O(1)Q^0(\alpha _{r},\nu ),\\ \delta _{2}&=\alpha _{r,2}+\mu _{w,2}\Delta \sigma _{\chi _{h}}+K_{w,2}\alpha _{l,1}+O(1)|\nu _{1}||\Delta \sigma _{\nu }|+O(1)Q^0(\alpha _{r},\nu ). \end{aligned}$$

This completes the proof. $\square$

### Lemma 4.8

 For $\Delta x$ sufficiently small,

$$\begin{aligned} |s_h-\sigma _{\chi }(h-1)|\geqq 6|\Delta \sigma _{\chi _{h}}|. \end{aligned}$$

### Proof

Using the notation as in Case 3, we have

$$\begin{aligned} \sigma _{\chi }(h)=\frac{\chi _{\Delta x,\vartheta }(x_{h})}{x_{h}},\qquad s_{h}=\dfrac{\chi _{\Delta x,\vartheta }(x_{h})-\chi _{\Delta x,\vartheta }(x_{h-1})}{\Delta x}. \end{aligned}$$

Then a direct computation leads to

$$\begin{aligned} |s_h-\sigma _{\chi }(h-1)|&=\left| \dfrac{\chi _{\Delta x,\vartheta }(x_{h})-\chi _{\Delta x,\vartheta }(x_{h-1})}{\Delta x}-\sigma _{\chi }(h-1)\right| \\&=\left| \dfrac{\sigma _{\chi }(h)x_{h}-\sigma _{\chi }(h-1)x_{h-1}}{\Delta x}-\sigma _{\chi }(h-1)\right| \\&=\left| \dfrac{x_{h}}{\Delta x}\right| |\sigma _{\chi }(h)-\sigma _{\chi }(h-1)|\\&\geqq 6\left| \sigma _{\chi }(h)-\sigma _{\chi }(h-1)\right| , \end{aligned}$$

for $\Delta x$ small enough. $\square$

Denote $\theta _{\chi }(h)=|\sigma _{\chi }(h-1)-s_{h}|$ that measures the angle between the leading shock $S_{\Delta x,\vartheta ,h}$ and the ray issuing from the origin and passing through $(x_{h-1},\chi _{\Delta x,\vartheta }(x_{h-1}))$. Then we have the following estimate for $\theta _{\chi }(h)$:

### Lemma 4.9

 For $M_{\infty }$ sufficiently large and $\Delta x$ sufficiently small,

$$\begin{aligned} \theta _{\chi }(h)-\theta _{\chi }(h+1)\geqq |\Delta \sigma _{\chi _{h}}|-|K_s||\alpha _{l,1}|, \end{aligned}$$

with $$h\geqq 0$$.

### Proof

We consider the following two different cases:

1. $$\sigma _{\chi }(h-1)<s_{h}$$ so that $$\sigma _{\chi }(h)>\sigma _{\chi }(h-1)$$.

 

- If $s_{h+1}>\sigma _{\chi }(h)$, then it follows from Lemma 4.7 that
  $$\begin{aligned} \theta _{\chi }(h)-\theta _{\chi }(h+1)&= s_{h}-\sigma _{\chi }(h-1)-\big (s_{h+1}-\sigma _{\chi }(h)\big )\\&= (1-\mu _s)\Delta \sigma _{\chi _{h}}-K_s\alpha _{l,1}\\&\geqq |\Delta \sigma _{\chi _{h}}|-|K_s||\alpha _{l,1}|. \end{aligned}$$
- If $s_{h+1}<\sigma _{\chi }(h)$, then, from Lemmas 4.7–4.8, we have
  $$\begin{aligned} \theta _{\chi }(h)-\theta _{\chi }(h+1)&=s_{h}-\sigma _{\chi }(h-1)-\big (\sigma _{\chi }(h)-s_{h+1}\big )\\&= 2(s_{h}-\sigma _{\chi }(h-1))+s_{h+1}-\sigma _{\chi }(h)-\big (s_{h}-\sigma _{\chi }(h-1)\big )\\&\geqq (11+\mu _s)|\Delta \sigma _{\chi _{h}}|+K_s\alpha _{l,1}\\&\geqq |\Delta \sigma _{\chi _{h}}|-|K_s||\alpha _{l,1}|. \end{aligned}$$

2. $$\sigma _{\chi }(h-1)>s_{h}$$ so that $$\sigma _{\chi }(h)<\sigma _{\chi }(h-1)$$.

 

- If $s_{h+1}>\sigma _{\chi }(h)$, then it follows from Lemmas 4.7–4.8 that
  $$\begin{aligned}&\theta _{\chi }(h)-\theta _{\chi }(h+1)\\&\quad =\sigma _{\chi }(h-1)-s_{h}-\big (s_{h+1}-\sigma _{\chi }(h)\big )\\&\quad =2(\sigma _{\chi }(h-1)-s_{h})+\sigma _{\chi }(h)-s_{h+1}-(\sigma _{\chi }(h-1)-s_{h})\\&\quad \geqq (11+\mu _s)|\Delta \sigma _{\chi _{h}}|-K_s\alpha _{l,1}\\&\quad \geqq |\Delta \sigma _{\chi _{h}}|-|K_s||\alpha _{l,1}|. \end{aligned}$$
- If $s_{h+1}<\sigma _{\chi }(h)$, then, from Lemma 4.7, we have
  $$\begin{aligned} \theta _{\chi }(h)-\theta _{\chi }(h+1)&=\sigma _{\chi }(h-1)-s_{h}-\big (\sigma _{\chi }(h)-s_{h+1}\big )\\&=(-1+\mu _s)\Delta \sigma _{\chi _{h}}+K_{s}\alpha _{l,1}\\&\geqq |\Delta \sigma _{\chi _{h}}|-|K_s||\alpha _{l,1}|. \end{aligned}$$

Note that we have used the fact that $\mu _s\in (-1,0)$ as $M_{\infty }\rightarrow \infty$ in above estimates. This completes the proof. $\square$

## Glimm-Type Functional and Compactness of the Approximate Solutions

For each $I\subset \cup _{k=1}^{h+1}\Omega _{\Delta x,\vartheta ,k}$, there exists $k_{I}$ with $1\leqq k_{I}\leqq h+1$ such that $I\cap \Gamma _{\Delta x,\vartheta , k_{I}}\ne \emptyset$. Next, as in [38, 51], we assign each mesh curve $I\subset \bigcup _{k=1}^{h+1}\Omega _{\Delta x,\vartheta ,k}$ with a Glimm-type functional $F_s(I)$; see also [10, 48].

### Definition 5.1

(**Weighted total variation**).  Define

$$\begin{aligned}&L_{0}^{(i)}(I)=\sum \{|\alpha _{i}|:\alpha _{i} \text { is the weak }i\text {-wave crossing }I\} \qquad \text{ for } i=1,\,2,\\&L_{1}(I)=\sum \{|\omega _{k}|: k>k_{I}\},\\&L_s(I)=\theta _\chi (I) \quad \text { for } \theta _{\chi }(I)=\theta _\chi (h)\text { in Lemma }4.9 \text { when }S_{\Delta x,\vartheta }\text { crossing }I,\\&L_b(I)=\theta _b(I) \quad \,\text { for }\theta _{b}(I)=\theta _b(h)\text { in Lemma }4.6\text { when }\Gamma _{\Delta x,\vartheta } \text { crossing }I. \end{aligned}$$

Then the weighted total variation is defined as

$$\begin{aligned} L(J)=L_{0}^{(1)}(I)+K_{2}L_{0}^{(2)}(I)+K_{1}L_{1}(I)+K_3L_s(I)+K_4L_b(I), \end{aligned}$$

where $K_{l}$ are positive constants for $l=1,2,3,4$.

Let

5.1 $$\begin{aligned} \sigma ^*=b_{0}+C_{1}\sum _{h=1}^{\infty }|\omega _{h}|,\qquad \sigma _*=s_{0}-\varpi , \end{aligned}$$

where $s_{0}$ is the velocity of the leading shock of the background solution, $\varpi$ and $C_{1}$ are constants to be determined; see also [10, 18, 48]. Note that $\varpi$ and $\sum _{h\geqq 1}|\omega _{h}|$ are chosen so small that the largeness of $M_{\infty }$ implies the smallness of $b_{0}-s_{0}$, which leads to the smallness of $\sigma ^*-\sigma _*$. We now define the total interaction potential.

### Definition 5.2

(**Total interaction potential**).  Define

$$\begin{aligned}&Q_{0}(I) =\sum \{|\alpha ||\beta |\,:\,\alpha \text { and }\beta \text { are weak waves crossing }I\text { and approach}\},\\&Q_{1}(I) =\sum \{|\alpha ||\sigma _\alpha -\sigma _*|\,:\,\alpha \text { is a weak }1-\text {wave crossing }I\},\\&Q_{2}(I)=\sum \{|\alpha ||\sigma ^*-\sigma _\alpha |\,:\,\alpha \text { is a weak }2-\text {wave crossing }I\}, \end{aligned}$$

where $\sigma _\alpha$ is the $\sigma$-coordinate of the grid point where $\alpha$ issues. Then the total interaction potential is defined as

$$\begin{aligned} Q(I)=Q_{0}(I)+2Q_{1}(I)+2Q_{2}(I). \end{aligned}$$

Now, we are able to define the Glimm-type functional.

### Definition 5.3

(**Glimm-type functional**).  Let

$$\begin{aligned} F(I)=L(I)+KQ(I), \end{aligned}$$

where *K* is a large real number to be chosen later.

Let

5.2 $$\begin{aligned} E_{\Delta x,\vartheta }(\Lambda )=\left\{ \begin{aligned}&Q(\Lambda )  &   (\text {defined in Case }1),&\\&\xi \big (|\alpha _{r,2}|+|\omega _{h+1}|+|\Delta \sigma _{b_{h}}|+Q(\Lambda _b)\big )  &   (\text {defined in Case }2),&\\&\xi \big (|\alpha _{l,1}|+|\Delta \sigma _{\chi _{h}}|+Q(\Lambda _s)\big )  &   (\text {defined in Case }3),&\end{aligned} \right. \end{aligned}$$

with $\xi >0$ sufficiently small and to be chosen later.

In order to make the Glimm-type functional monotonically decreasing, we have to choose the weights carefully in the functional, based on the underlying features of the wave interactions governed by the system. Indeed, we have the following lemma (*cf.* [48]):

### Lemma 5.1

Let $K_{r,1}$, $K_{w,2}$, $K_s$, and $\mu _{w,2}$ be given by Lemmas 4.3 and 4.7. Then

$$\begin{aligned} \lim _{M_{\infty }\rightarrow \infty } \big (|K_{r,1}||K_{w,2}|+|K_{r,1}||K_s||\mu _{w,2}|\big )<1. \end{aligned}$$

### Proof

Lemmas 2.8–2.9 give

$$\begin{aligned}&\lim _{M_{\infty }\rightarrow \infty }|K_{r,1}||K_{w,2}|=\left| \dfrac{\sin (\theta _{0}+\theta _{m}^0)}{\sin (\theta _{0}-\theta _{m}^0)} \right| ,\\&\lim _{M_{\infty }\rightarrow \infty }|K_{r,1}||K_s||\mu _{w,2}|\\&\quad =\dfrac{\cos ^2(\theta _{0}+\theta _{m}^0)}{\cos ^2(\theta _{0}-\theta _{m}^0)} \\&\qquad \times \lim _{M_{\infty }\rightarrow \infty } \left| \dfrac{\det \big (r_{1}(U),r_{2}(U)\big )}{\det \big (r_{2}(G(s_{0}),G_s(s_{0}))\big )} \right| \left| \dfrac{\det ((\partial {\tilde{U}})/(\partial \Delta \sigma _{\chi _{h}}),G_s(s_{0};U_{\infty }))}{\det (r_{2}(G(s_{0};U_{\infty })),G_s(s_{0};U_{\infty }))}\right| \\&\quad =\dfrac{\sin 2\theta _{m}^0\cos \theta _{0}|\sin \theta _{0}|}{\sin ^2(\theta _{0}-\theta _{m}^0)}. \end{aligned}$$

Note that $\theta _{0}\in (-\frac{\pi }{2},0)$, $\theta _{0}\pm \theta _{m}^0\in (-\frac{\pi }{2},\frac{\pi }{2})$, and $\theta _{m}^0\in (0,\frac{\pi }{2})$. Then, when $\theta _{0}+\theta _{m}^0<0$,

$$\begin{aligned} \lim _{M_{\infty }\rightarrow \infty } \big (|K_{r,1}||K_{w,2}|+|K_{r,1}||K_s||\mu _{w,2}|\big ) <\dfrac{2\sin \theta _{m}^0\cos \theta _{0}-\sin (\theta _{0}+\theta _{m}^0)}{\sin (\theta _{m}^0-\theta _{0})}=1; \end{aligned}$$

when $$\theta _{0}+\theta _{m}^0>0$$,

$$\begin{aligned} \lim _{M_{\infty }\rightarrow \infty }\big (|K_{r,1}||K_{w,2}|+|K_{r,1}||K_s||\mu _{w,2}|\big ) <\dfrac{2\cos \theta _{m}^0|\sin \theta _{0}|+\sin (\theta _{0}+\theta _{m}^0)}{\sin (\theta _{m}^0-\theta _{0})}=1. \end{aligned}$$

This implies the expected result. $\square$

At this stage, we are able to choose the coefficients in the Glimm-type functional (*cf.* [48]).

### Lemma 5.2

There exist positive constants $K_{2}$ and $K_3$ such that

$$\begin{aligned}&\lim _{M_{\infty }\rightarrow \infty }\big (K_{2}|K_{w,2}|+K_3|K_s|\big )<1,  &   \lim _{M_{\infty }\rightarrow \infty }\big (K_{2}|\mu _{w,2}|-K_3\big )<0,\\&\lim _{M_{\infty }\rightarrow \infty }\big (K_{2}-|K_{r,1}|\big )>0.  &   \end{aligned}$$

### Proof

Let $K_{r,1}^*=\lim _{M_{\infty }\rightarrow \infty }|K_{r,1}|$, $K_{w,2}^*=\lim _{M_{\infty }\rightarrow \infty }|K_{w,2}|$, $K_s^*=\lim _{M_{\infty }\rightarrow \infty }|K_s|$, and $\mu _{w,2}^*=\lim _{M_{\infty }\rightarrow \infty }|\mu _{w,2}|$. Then, by Lemma 5.1,

$$\begin{aligned} K_{r,1}^*\big (K_{w,2}^*+K_s^*\mu _{w,2}^*\big )<1. \end{aligned}$$

Hence, we choose $K_{2}$ such that

$$\begin{aligned} K_{2}>K_{r,1}^*,\qquad K_{2}\big (K_{w,2}^*+K_s^*\mu _{w,2}^*\big )<1, \end{aligned}$$

which implies

$$\begin{aligned} K_{2}K_s^*\mu _{w,2}^*<1-K_{2}K_{w,2}^*. \end{aligned}$$

Then we take $K_3$ such that

$$\begin{aligned} K_3>K_{2}\mu _{w,2}^*,\qquad K_3K_s^*<1-K_{2}K_{w,2}^*, \end{aligned}$$

and the proof is complete. $\square$

With the coefficients chosen properly, we can derive a decay property for the Glimm-type functional.

### Proposition 5.1

Let $M_{\infty }$ be sufficiently large, and let $\sigma ^{*}-\sigma _{*}$ and $\sum _{h\geqq 1}|\omega _{h}|$ be sufficiently small. Let *I* and *J* be a pair of space-like mesh curves with *J* being an immediate successor of *I*. The region bounded by the difference between *I* and *J* is denoted as $\Lambda$. Then there exist positive constants $\varepsilon _\infty$, *K*, and $K_{l}$ for $l=1,2,3,4$, such that, if $F(I)<\varepsilon _\infty$, then

$$\begin{aligned} F(I)\leqq F(J)-\frac{1}{4}E_{\Delta x, \vartheta }(\Lambda ), \end{aligned}$$

where $E_{\Delta x, \vartheta }(\Lambda )$ is given by (5.2).

### Proof

 When $M_{\infty }$ is large enough, according to Lemma 5.2, there are constants $K_{2}$ and $K_3$ so that

$$\begin{aligned}&K_{2}|K_{w,2}|+K_3|K_s|<1-\xi _{0},\quad K_{2}|\mu _{w,2}|-K_3<-\xi _{0},\quad \\&K_{2}-|K_{r}|-K_4|K_{c,2}|>\xi _{0} \end{aligned}$$

for some $$\xi _{0}>0$$.

Now, as in [38], we prove the result inductively; see also [10, 48]. We consider three special cases as in §4, depending on the location of $\Lambda$. From now on, we use *C* to denote a universal constant depending only on the system, which may be different at each occurrence.

**Case** 1. $\Lambda$ lies between $\Gamma _{\Delta x,\vartheta }$ and $S_{\Delta x, \vartheta }$. We consider the case as in Lemma 4.1. Notice that

$$\begin{aligned}&(L_{0}^{(1)}+K_{2}L_{0}^{(2)})(J)-(L_{0}^{(1)}+K_{2}L_{0}^{(2)})(I)\leqq CQ(\Lambda ),\\&L_b(J)-L_b(I)=0,\\&(K_{1}L_{1}+K_3L_s)(J)-(K_{1}L_{1}+K_3L_s)(I)=0. \end{aligned}$$

Then we obtain

$$\begin{aligned} L(J)-L(I)\leqq CQ(\Lambda ). \end{aligned}$$

For the terms contained in *Q*, we have

$$\begin{aligned} Q_{0}(J)-Q_{0}(I)\leqq CL(I)Q(\Lambda )-Q^0(\Lambda ). \end{aligned}$$

For **Case** 1.1:

$$\begin{aligned} (Q_{1}+Q_{2})(J)-(Q_{1}+Q_{2})(I)&{=} |\delta _{1}|(\sigma _{2}{-}\sigma _*){-}|\alpha _{1}|(\sigma _{2}-\sigma _*){-}|\beta _{1}|(\sigma _{1}-\sigma _*)\\&\quad +|\delta _{2}|(\sigma ^*-\sigma _{2})-|\alpha _{2}|(\sigma ^*-\sigma _{2})\\&\leqq C(\sigma ^*-\sigma _*)Q(\Lambda )-|\Delta \sigma ||\beta _{1}|. \end{aligned}$$

For **Case** 1.2:

$$\begin{aligned} (Q_{1}+Q_{2})(J)-(Q_{1}+Q_{2})(I)&\leqq |\delta _{1}|(\sigma _{1}-\sigma _*)-|\beta _{1}|(\sigma _{1}-\sigma _*)\\&\quad +|\delta _{2}|(\sigma ^*-\sigma _{1})-|\alpha _{2}|(\sigma ^*-\sigma _{2})\\&\quad -|\beta _{2}|(\sigma ^*-\sigma _{1})\\&\leqq C(\sigma ^*-\sigma _*)Q(\Lambda )-|\Delta \sigma ||\alpha _{2}|, \end{aligned}$$

which gives

$$\begin{aligned} Q(J)-Q(I)\leqq&-\big (1-C(L(I)+\sigma ^*-\sigma _*)\big )Q(\Lambda ). \end{aligned}$$

When *L*(*I*) and $\sigma ^*-\sigma _*$ are small enough, and *K* is sufficiently large, it follows that

$$\begin{aligned} F(J)-F(I)\leqq -\left\{ K\big (1-C(L(I)+\sigma ^*-\sigma _*)\big )-C\right\} Q(\Lambda )\leqq -\frac{1}{4}Q(\Lambda ). \end{aligned}$$

**Case** 2. $\Lambda _b=\Delta _{h,n_{b,h}-1}\cup \Delta _{h,n_{b,h}}\cup \Delta _{h,n_{b,h}+1}$ covers a part of $\Gamma _{\Delta x,\vartheta }$ but none of $S_{\Delta x, \vartheta }$. Direct computation shows that

$$\begin{aligned}&L_{0}^{(1)}(J)-L_{0}^{(1)}(I)\leqq |K_{r,1}||\alpha _{r,2}| +|K_{\sigma ,1}||\Delta \sigma _{b_{h}}|+|K_{b,1}||\omega _{h+1}|+CQ(\Lambda _b),\\&L_{0}^{(2)}(J)-L_{0}^{(2)}(I)\leqq -|\alpha _{r,2}|+|K_{r,2}||\alpha _{r,2}| +|K_{\sigma ,2}||\Delta \sigma _{b_{h}}|\\&\qquad \qquad \qquad \qquad +|K_{b,2}||\omega _{h+1}|+CQ(\Lambda _b),\\&L_{1}(J)-L_{1}(I)=-|\omega _{h+1}|,\\&L_s(J)-L_s(I)=0,\\&L_b(J)-L_b(I)=-|\Delta \sigma _{b_{h}}|+|K_{c,2}||\alpha _{r,2}|+C|\omega _{h+1}|+C|\alpha _{r,1}||\Delta \sigma _{\alpha }|. \end{aligned}$$

Combining the above estimates together, we obtain

$$\begin{aligned} L(J)-L(I)&\leqq -\big (K_{2}-|K_{r,1}|-K_4|K_{c,2}|-K_{2}|K_{r,2}|\big )|\alpha _{r,2}|\\&\quad -\big (K_{1}-|K_{b,1}|-K_{2}|K_{b,2}|-CK_4\big )|\omega _{h+1}|\\&\quad -\big (K_4-|K_{\sigma ,1}|-K_{2}|K_{\sigma ,2}|\big )|\Delta \sigma _{b_{h}}|+CQ(\Lambda _b)+C|\alpha _{r,1}||\Delta \sigma _{\alpha }|. \end{aligned}$$

For the terms contained in *Q*, noting that $|\Delta \sigma _{\alpha }|\leqq |\Delta \sigma _{\nu }|$, we have

$$\begin{aligned} Q_{0}(J)-Q_{0}(I)&\leqq -Q^0((\alpha _{1},0),\nu )+CL(I)\big (|\alpha _{r,2}| +|\Delta \sigma _{b_{h}}|\\&\quad +|\omega _{h+1}|+CQ(\Lambda _b)\big ),\\ Q_{1}(J)-Q_{1}(I)&=|\delta _{1}|(\sigma _{\nu }-\sigma _*)-(|\alpha _{l,1}|+|\alpha _{r,1}|)(\sigma _{\alpha }-\sigma _*)-|\nu _{1}|(\sigma _{\nu }-\sigma _*)\\&\leqq -(|\alpha _{l,1}|+|\alpha _{r,1}|)|\Delta \sigma _{\nu }|+C(\sigma ^*-\sigma _*)\big (|\alpha _{r,2}|\\&\quad +|\Delta \sigma _{b_{h}}|+|\omega _{h+1}|+CQ(\Lambda _b)\big ),\\ Q_{2}(J)-Q_{2}(I)&=|\delta _{2}|(\sigma ^*-\sigma _{\nu })-|\alpha _{r,2}|(\sigma ^*-\sigma _{\alpha })-|\nu _{2}|(\sigma ^*-\sigma _{\nu })\\&\leqq C(\sigma ^*-\sigma _*)\big (|\alpha _{r,2}| +|\Delta \sigma _{b_{h}}|+|\omega _{h+1}|+CQ(\Lambda _b)\big ). \end{aligned}$$

Then we conclude

$$\begin{aligned}&Q(J)-Q(I)\\&\quad \leqq -Q^0((\alpha _{1},0),\nu )+CL(I)\big (|\alpha _{r,2}| +|\Delta \sigma _{b_{h}}|+|\omega _{h+1}|+CQ(\Lambda _b)\big )-|\alpha _{1}||\Delta \sigma _{\nu }|\\&\quad \quad -|\alpha _{r,1}||\Delta \sigma _{\alpha }|+2C(\sigma ^*-\sigma _*)\big (|\alpha _{r,2}| +|\Delta \sigma _{b_{h}}|+|\omega _{h+1}|+CQ(\Lambda _b)\big )\\&\quad \leqq -\big (1-C(L(I)+\sigma ^*-\sigma _*)\big )Q(\Lambda _b)\\&\quad \quad +C\big (L(I)+\sigma ^*-\sigma _*\big )\big (|\alpha _{r,2}| +|\Delta \sigma _{b_{h}}|+|\omega _{h+1}|\big ). \end{aligned}$$

Finally, combining all the estimates above together, we obtain

$$\begin{aligned}&F(J)-F(I)\\&\quad \leqq -\left\{ K\big (1-C(L(I)+\sigma ^*-\sigma _*)\big )- C\right\} Q(\Lambda _b)\\&\quad \quad -\left\{ K_{2}-|K_{r,1}|-K_4|K_{c,2}|-K_{2}|K_{r,2}|-KC\big (L(I)+\sigma ^*-\sigma _*\big )\right\} |\alpha _{r,2}|\\&\quad \quad -\left\{ K_{1}-|K_{b,1}|-K_{2}|K_{b,2}|-CK_4-KC\big (L(I)+\sigma ^*-\sigma _*\big )\right\} |\omega _{h+1}|\\&\quad \quad -\left\{ K_4-|K_{\sigma ,1}|-K_{2}|K_{\sigma ,2}|-KC\big (L(I)+\sigma ^*-\sigma _*\big )\right\} |\Delta \sigma _{b_{h}}|. \end{aligned}$$

Taking suitably large $K_{1}$, then, when *K* is sufficiently large, and *L*(*I*) and $\sigma ^*-\sigma _*$ are sufficiently small, we conclude

$$\begin{aligned} F(J)-F(I)\leqq -\frac{\xi }{4}\big (|\alpha _{r,2}|+|\omega _{h+1}|+|\Delta \sigma _{b_{h}}|+Q(\Lambda _b)\big ) \end{aligned}$$

for some $\xi >0$ small enough.

**Case** 3. $\Lambda _{s}=\Delta _{h,n_{\chi ,h}-1}\cup \Delta _{h,n_{\chi ,h}}\cup \Delta _{h,n_{\chi ,h}+1}$ covers a part of $S_{\Delta x,\vartheta }$ but none of $\Gamma _{\Delta x, \vartheta }$. A direct computation shows that

$$\begin{aligned}&L_{0}^{(1)}(J)-L_{0}^{(1)}(I)\leqq -|\alpha _{l,1}|+|K_{w,1}||\alpha _{l,1}|+|\mu _{w,1}||\Delta \sigma _{\chi _{h}}|+CQ(\Lambda _{s}),\\&L_{0}^{(2)}(J)-L_{0}^{(2)}(I) \leqq |K_{w,2}||\alpha _{l,1}|+|\mu _{w,2}||\Delta \sigma _{\chi _{h}}|+CQ(\Lambda _{s}),\\&L_{1}(J)-L_{1}(I)=0,\\&L_s(J)-L_s(I)\leqq -|\Delta \sigma _{\chi _{h}}|+|K_s||\alpha _{l,1}|,\\&L_b(J)-L_b(I)=0. \end{aligned}$$

Combine the above estimates together, we obtain

$$\begin{aligned}&L(J)-L(I)\\&\quad \leqq -(1-|K_{w,1}|-K_{2}|K_{w,2}|-K_3|K_s|)|\alpha _{l,1}|\\&\quad \quad -\big (K_3-|\mu _{w,1}|-K_{2}|\mu _{w,2}|\big )|\Delta \sigma _{\chi _{h}}|+CQ(\Lambda _{s})\\&\quad \leqq -\big (1-K_{2}|K_{w,2}|-K_3|K_s|-|K_{w,1}|\big )|\alpha _{l,1}|\\&\quad \quad -\big (K_3-K_{2}|\mu _{w,2}|-|\mu _{w,1}|\big )|\Delta \sigma _{\chi _{h}}|+CQ(\Lambda _{s}). \end{aligned}$$

For the terms contained in *Q*, we have

$$\begin{aligned} Q_{0}(J)-Q_{0}(I)&\leqq -Q^0(\alpha _{r},\nu ) +CL(I)\big (|\alpha _{l,1}|+|\Delta \sigma _{\chi _{h}}|+Q(\Lambda _{s})\big ),\\ Q_{1}(J)-Q_{1}(I)&= |\delta _{1}|(\sigma _{\alpha }-\sigma _*)-(|\alpha _{l,1}|+|\alpha _{r,1}|)(\sigma _{\alpha }-\sigma _*)-|\nu _{1}|(\sigma _{\nu }-\sigma _*)\\&\leqq -|\nu _{1}||\Delta \sigma _{\nu }|+C(\sigma ^*-\sigma _*)\big (|\alpha _{l,1}|+|\Delta \sigma _{\chi _{h}}|+Q(\Lambda _{s})\big ),\\ Q_{2}(J)-Q_{2}(I)&= |\delta _{2}|(\sigma ^*-\sigma _{\nu })-|\alpha _{r,2}|(\sigma ^*-\sigma _{\alpha })\\&\leqq C(\sigma ^*-\sigma _*)\big (|\alpha _{l,1}|+|\Delta \sigma _{\chi _{h}}|+Q(\Lambda _{s})\big ). \end{aligned}$$

Then we deduce that

$$\begin{aligned} Q(J)-Q(I)\leqq&-\big (1-C(L(I)+\sigma ^*-\sigma _*)\big )Q(\Lambda _{s})\\&+C\big (L(I)+\sigma ^*-\sigma _*\big )\big (|\alpha _{l,1}|+|\Delta \sigma _{\chi _{h}}|\big ). \end{aligned}$$

Finally, combining all the estimates above together, we obtain

$$\begin{aligned}&F(J)-F(I)\\&\quad \leqq -\left\{ K\big (1-C(L(I)+\sigma ^*-\sigma _*)\big ) -C\right\} Q(\Lambda _{s})\\&\quad \quad -\Big \{1-K_{2}|K_{w,2}|-K_3|K_s|-|K_{w,1}|-CK\big (L(I)+\sigma ^*-\sigma _*\big )\Big \}|\alpha _{l,1}|\\&\quad \quad -\Big \{K_3-K_{2}|\mu _{w,2}|-|\mu _{w,1}|-CK\big (L(I)+\sigma ^*-\sigma _*\big )\Big \}|\Delta \sigma _{\chi _{h}}|. \end{aligned}$$

When *K* is sufficiently large, and *L*(*I*) and $\sigma ^*-\sigma _*$ are sufficiently small, we conclude that

$$\begin{aligned} F(J)-F(I)\leqq&-\frac{\xi }{4}(Q(\Lambda _{s})+|\alpha _{l,1}|+|\Delta \sigma _{\chi _{h}}|) \end{aligned}$$

for some $\xi >0$ small enough. Combining the above three cases, we conclude our result. $\square$

Now, let $I_{h}$ be the mesh curve in the stripe: $\{(x,y):\, x_{h-1}\leqq x\leqq x_{h}\}$ for $h\in {\mathbb {N}}_{+}$; that is, $I_{h}$ connects all the mesh points in the strip. Let *I* and *J* be any pair of mesh curves with $I_{h}<I<J<I_{h+1}$, and let *J* be an immediate successor of *I*. That is, the mesh points on *J* differ from those on *I* by only one point generally (except three points near the approximate boundary or near the approximate shock), and the region bounded by the difference between *I* and *J* is denoted by $\Lambda$. Proposition 5.1 suggests that the total variation of the approximate solutions is uniformly bounded.

Moreover, we have the following estimates for the approximate boundary and the approximate leading shock:

### Proposition 5.2

There exists a constant ${\bar{C}}>0$, independent of $\Delta x$, $\vartheta$, and $U_{\Delta x, \vartheta }$, such that

$$\begin{aligned}&\text {T.V.}\{s_{\Delta x,\vartheta }:[0,\infty )\}=\sum _{h=0}^{\infty }|s_{h+1}-s_{h}|\leqq {\bar{C}}\sum _{h\geqq 1}|\omega _{h}|,\\&\text {T.V.}\{b'_{\Delta x,\vartheta }:[0,\infty )\}=\sum _{h=0}^{\infty }|b'_{h+1}-b'_{h}|\leqq {\bar{C}}\sum _{h\geqq 1}|\omega _{h}|. \end{aligned}$$

### Proof

Notice that

$$\begin{aligned} \text {T.V.}\{s_{\Delta x,\vartheta }:[0,\infty )\}&=\sum _{h=0}^{\infty }|s_{h+1}-s_{h}|\leqq O(1)\sum _{\Lambda _s}E_{\Delta x,\vartheta }(\Lambda _s)\\&\leqq O(1)\sum _{\Lambda }F(I)-F(J)\leqq O(1)F(I_{1}). \end{aligned}$$

Similarly, we have

$$\begin{aligned} \text {T.V.}\{b'_{\Delta x,\vartheta }:[0,\infty )\}\leqq O(1)F(I_{1}). \end{aligned}$$

Therefore, ${\bar{C}}$ in the statement can be determined. $\square$

We choose $C_{1}=2{\bar{C}}$ and $\varpi =2{\bar{C}}\sum _{h\geqq 1}|\omega _{h}|$ in (5.1). The largeness of $M_{\infty }$ and the smallness of $\sum _{h\geqq 1}|\omega _{h}|$ imply the smallness of $\sigma ^{*}-\sigma _{*}$. Then, following [14, 52], we conclude

### Theorem 5.1

Under assumptions **(A1)**–**(A2)**, if $M_{\infty }$ is sufficiently large and $\sum _{h\geqq 1}|\omega _{h}|$ is sufficiently small, then, for any $\vartheta \in \Pi _{h=0}^{\infty }[0,1)$ and $\Delta x>0$, the modified Glimm scheme introduced above defines a sequence of global approximate solutions $U_{\Delta x,\vartheta }(x,y)$ such that

$$\begin{aligned}&\sup _{x>0}\text {T.V.}\{U_{\Delta x,\vartheta }(x,y):(-\infty ,b_{\Delta x,\vartheta }(x))\}<\infty ,\\&\int _{-\infty }^0|U_{\Delta x,\vartheta }(x_{1},y+b_{\Delta x,\vartheta }(x_{1}))-U_{\Delta x,\vartheta }(x_{2},y+b_{\Delta x,\vartheta }(x_{2}))|\,{\textrm{d}}y< L_{1}|x_{1}-x_{2}| \end{aligned}$$

for some $L_{1}>0$ independent of $U_{\Delta x,\vartheta }$, $\Delta x$, and $\vartheta$.

## Convergence of the Approximate Solutions

In Section 5, the uniform bound of the total variation of the approximate solutions $U_{\Delta x,\vartheta }$ has been obtained. Then, by Propositions 5.1–5.2, the existence of convergent subsequences of the approximate solutions $\{U_{\Delta x,\vartheta }\}$ follows. Now we are going to prove that there is a convergent subsequence of the approximate solutions $\{U_{\Delta x,\vartheta }\}$ whose limit is an entropy solution to our problem.

Take $\Delta x=2^{-m}$, $m=0,1,2,\cdots$. For any randomly chosen sequence $\vartheta =(\vartheta _{0},\vartheta _{1},\vartheta _{2},\cdots ,\vartheta _{h},\cdots )$, we obtain a set of approximate solutions, which are denoted by $\{(u_m,v_m)\}$. It suffices to prove that there is a subsequence (still denoted by) $\{(u_m,v_m)\}$ such that, as $m\rightarrow \infty$,

6.1 $$\begin{aligned}&\iint _{\Omega _{\Delta x,\vartheta }}\Big (\phi _x\rho _m u_m+\phi _y\rho _m v_m -\frac{\rho _m v_m\phi }{y}\Big )\,{\textrm{d}}x {\textrm{d}}y \nonumber \\&\qquad +\int _{-\infty }^{y_{0}(0)}\phi (x_{0},y)\rho (x_{0},y)u(x_{0},y)\,{\textrm{d}}y\rightarrow 0 \end{aligned}$$

for any $\phi (x,y)\in \text {C}_{0}^1({\mathbb {R}}^2;{\mathbb {R}})$, and

6.2 $$\begin{aligned} \iint _{\Omega _{\Delta x,\vartheta }}\left( \phi _x v_m-\phi _y u_m\right) {\textrm{d}}x {\textrm{d}}y\rightarrow 0 \end{aligned}$$

for any $\phi (x,y)\in \text {C}_{0}^1(\Omega ;{\mathbb {R}})$. We now prove (6.1) only, since (6.2) can be deduced analogously.

For simplicity, we drop the subscript of $(u_m,v_m)$, and rewrite (6.1) as

$$\begin{aligned}&\iint _{\Omega _{\Delta x,\vartheta }} \Big (\phi _x\rho u+\phi _y\rho v-\frac{\rho v\phi }{y}\Big )\,{\textrm{d}}x {\textrm{d}}y +\int _{-\infty }^{y_{0}(0)}\phi (x_{0},y)\rho (x_{0},y)u(x_{0},y)\,{\textrm{d}}y\\&\quad =\sum _{h=1}^{\infty }\iint _{\Omega _{\Delta x,\vartheta ,h}} \Big (\phi _x\rho u+\phi _y\rho v-\frac{\rho v\phi }{y}\Big )\,{\textrm{d}}x {\textrm{d}}y \\&\qquad +\int _{-\infty }^{y_{0}(0)}\phi (x_{0},y)\rho (x_{0},y)u(x_{0},y)\, {\textrm{d}}y. \end{aligned}$$

By the shock waves and the upper/lower edges of rarefaction waves, each $\Omega _{\Delta x,\vartheta ,h}$ can be divided into smaller polygons: $\Omega _{\Delta x,\vartheta ,h,j}$, $j=0,-1,-2,\cdots$, alternatively, where $\Omega _{\Delta x,\vartheta ,h,0}$ is the uppermost area below the approximate boundary $\Gamma _{\Delta x,\vartheta ,h}$. Then we have

$$\begin{aligned}&\iint _{\Omega _{\Delta x,\vartheta }} \Big (\phi _x\rho u+\phi _y\rho v-\frac{\rho v\phi }{y}\Big )\,{\textrm{d}}x{\textrm{d}}y +\int _{-\infty }^{y_{0}(0)}\phi (x_{0},y)\rho (x_{0},y)u(x_{0},y)\,{\textrm{d}}y\\&\quad =\sum _{h=1}^{\infty }\sum _{j=0}^{-\infty }\iint _{\Omega _{\Delta x,\vartheta ,h,j}} \Big (\phi _x\rho u+\phi _y\rho v-\frac{\rho v\phi }{y}\Big )\,{\textrm{d}}x {\textrm{d}}y\\&\quad \quad +\int _{-\infty }^{y_{0}(0)}\phi (x_{0},y)\rho (x_{0},y)u(x_{0},y)\,{\textrm{d}}y\\&\quad =-\sum _{h,j}\iint _{\Omega _{\Delta x,\vartheta ,h,j}} \phi \Big ((\rho u)_x+(\rho v)_y+\frac{\rho v}{y}\Big )\,{\textrm{d}}x {\textrm{d}}y\\&\quad \quad +\sum _{h,j}\iint _{\Omega _{\Delta x,\vartheta ,h,j}} \big ( (\phi \rho u)_x+(\phi \rho v)_y\big )\,{\textrm{d}}x {\textrm{d}}y\\&\quad \quad +\int _{-\infty }^{y_{0}(0)}\phi (x_{0},y)\rho (x_{0},y)u(x_{0},y)\,{\textrm{d}}y\\&\quad =:\text {I}+\text {II} +\text {III}. \end{aligned}$$

We first have

### Proposition 6.1

   I $$\rightarrow 0\,\,$$ as $$\Delta x\rightarrow 0$$.

### Proof

 To deal with the first term I, we use the transform:

6.3 $$\begin{aligned} \sigma =\frac{y}{x},\qquad \eta =\frac{y-y_n(h)}{x-x_{h}}, \end{aligned}$$

where $(x_{h},y_n(h))$ is the center of the Riemann problem, and *n* depends on *j*. Then we obtain

6.4 $$\begin{aligned} \begin{aligned} \text {I} =&\sum _{h,j}\iint \frac{(x-x_h)\phi \rho }{\sigma (\eta -\sigma )} \Big (\sigma ^2\big (1-\frac{u^2}{c^2}\big ) u_{\sigma }-\frac{2uv\sigma ^2}{c^2}v_{\sigma }-\big (1-\frac{v^2}{c^2}\big ) v_{\sigma }\sigma -v\Big )\,{\textrm{d}}\eta {\textrm{d}}\sigma \\&-\sum _{h,j}\iint \frac{x\phi }{\eta -\sigma } \big (-\eta (\rho u)_{\eta }+(\rho v)_{\eta }\big )\,{\textrm{d}}\eta {\textrm{d}}\sigma . \end{aligned} \end{aligned}$$

From the construction of the approximate solutions, the first term of (6.4) vanishes. For the second term, we have

$$\begin{aligned} -\eta (\rho u)_{\eta }+(\rho v)_{\eta }=O(1)\Delta \sigma , \end{aligned}$$

where $\Delta \sigma$ is the change of the $\sigma -$coordinate in domain $\Omega _{\Delta x,\vartheta ,h,j}$. Denote the rarefaction waves in $\Omega _{\Delta x,\vartheta ,h}$ alternatively by $\alpha _{R,h,i}$. Then we have

$$\begin{aligned} \text {I}=O(1)\sum _{h,j}\Delta \eta (\Delta \sigma )^2, \end{aligned}$$

with $\Delta \eta =O(1)\alpha _{R,h,i}$. According to Proposition 5.1, the total strength $\sum _{i}|\alpha _{R,h,i}|$ of rarefaction waves in $\Omega _{\Delta x,\vartheta ,h}$ is bounded, so that

6.5 $$\begin{aligned} \text {I}=O(1)\,\text {diam}(\text {supp}\,\phi )\,\Delta x, \end{aligned}$$

which gives desired result. $\square$

Next, applying Green’s formula in each $\Omega _{\Delta x,\vartheta ,h,j}$, we obtain

6.6 $$\begin{aligned} \begin{aligned} \text {II}+\text {III}&=\sum _{h=1}^{\infty }\int _{-\infty }^{b_{\Delta x,\vartheta }(x_{h})}\phi (x_{h},y)\big (\rho (x_{h}-,y)u(x_{h}-,y)-\rho (x_{h}+,y)u(x_{h}+,y)\big )\,{\textrm{d}}y\\&\quad \, +\sum _{h=0}^{\infty }\int _{x_{h}}^{x_{h+1}}\phi (x,b(x)) \rho (x,b(x)) \big (v(x,b(x))-u(x,b(x))b'(x)\big ){\textrm{d}}x\\&\quad \, +\sum _{h,i}\int _{W_{h,i}} \big (s_{h,i}(\rho ^+u^+-\rho ^-u^-)-(\rho ^+v^+-\rho ^-v^-)\big )\phi \,{\textrm{d}}x\\&=: \text {IV}+\text {V}+\text {VI}, \end{aligned} \end{aligned}$$

where $W_{h,i}=\{(x,y):\,y=w_{h,i}(x)=s_{h,i}(x-x_{h})+y_n(h)\,\text { for some }n\}$ are shock waves or upper/lower edges of rarefaction waves lying in $\Omega _{\Delta x,\vartheta ,h}$, and $\rho ^\pm =\rho (x,w_{i,h}(x)\pm )$, $u^\pm =u(x,w_{i,h}(x)\pm )$, and $v^\pm =v(x,w_{i,h}(x)\pm )$.

We now show

### Proposition 6.2

There exists a subsequence of $\{(u_m,v_m)\}$ such that IV $\rightarrow 0\,\,$ as $m\rightarrow \infty$.

### Proof

The first term on the right-hand side of (6.6) can be rewritten as

$$\begin{aligned} \text {IV}=\sum _{h\geqq 1}V_{h} \end{aligned}$$

with

$$\begin{aligned} V_{h}&=\sum _{n=n_{\chi ,h}+1}^{n_{b,h}-1}\int _{y_{n-1}(h)}^{y_{n}(h)}\phi (x_{h},y)\big (\rho (x_{h}-,y)u(x_{h}-,y)-\rho (x_{h}+,y)u(x_{h}+,y)\big )\,{\textrm{d}}y\\&\quad \,+\int _{\chi _{\Delta x,\vartheta }(x_{h})}^{y_{n_{\chi ,h}}(h)+1}\phi (x_{h},y)\big (\rho (x_{h}-,y)u(x_{h}-,y)-\rho (x_{h}+,y)u(x_{h}+,y)\big )\,{\textrm{d}}y\\&\quad \,+\int _{y_{n_{b,h}}(h)-1}^{b_{\Delta x,\vartheta }(x_{h})}\phi (x_{h},y)\big (\rho (x_{h}-,y)u(x_{h}-,y)-\rho (x_{h}+,y)u(x_{h}+,y)\big )\,{\textrm{d}}y. \end{aligned}$$

To show $\text {IV}\rightarrow 0$ for some subsequence $\{(u_m,v_m)\}$, we now introduce

$$\begin{aligned} {\widetilde{V}}=\sum _{h\geqq 1}{\widetilde{V}}_{h} \end{aligned}$$

with

$$\begin{aligned} {\widetilde{V}}_{h}&=\sum _{n=n_{\chi ,h}+1}^{n_{b,h}-1}\int _{y_{n-1}(h)}^{y_{n}(h)}\phi (x_{h},y_n(h))\big (\rho (x_{h}-,y)u(x_{h}-,y)-\rho (x_{h}+,y)u(x_{h}+,y)\big )\,{\textrm{d}}y\\&\quad \,+\int _{y_{n_{b,h}}(h)}^{b_{\Delta x,\vartheta }(x_{h})}\phi (x_{h},y_{n_{b,h}+1}(h))\big (\rho (x_{h}-,y)u(x_{h}-,y)-\rho (x_{h}+,y)u(x_{h}+,y)\big )\,{\textrm{d}}y\\&\quad \,+\int _{y_{n_{b,h}-1}(h)}^{y_{n_{b,h}}(h)}\phi (x_{h},y_{n_{b,h}}(h))\big (\rho (x_{h}-,y)u(x_{h}-,y)-\rho (x_{h}+,y)u(x_{h}+,y)\big ){\textrm{d}}y\\&\quad \,+\int _{y_{n_{\chi ,h}}(h)}^{y_{n_{\chi ,h}+1}(h)}\phi (x_{h},y_{n_{\chi ,h}+1}(h))\big (\rho (x_{h}-,y)u(x_{h}-,y)-\rho (x_{h}+,y)u(x_{h}+,y)\big )\,{\textrm{d}}y\\&\quad \,+\int _{\chi _{\Delta x,\vartheta }(x_{h})}^{y_{n_{\chi ,h}}(h)}\phi (x_{h},y_{n_{\chi ,h}}(h))\big (\rho (x_{h}-,y)u(x_{h}-,y)-\rho (x_{h}+,y)u(x_{h}+,y)\big )\,{\textrm{d}}y\\&=:\sum _{n=n_{\chi ,h}+1}^{n_{b,h}-1}\int _{y_{n-1}(h)}^{y_{n}(h)}\phi (x_{h},y_n(h))\big (\rho (x_{h}-,y)u(x_{h}-,y)-\rho (x_{h}+,y)u(x_{h}+,y)\big )\,{\textrm{d}}y\\&\quad \,+{\check{V}}_{h}^{(0)}+\check{V}_{h}^{(1)}+{\hat{V}}_{h}^{(1)}+{\hat{V}}_{h}^{(0)}. \end{aligned}$$

From the construction of approximate solutions, we have

$$\begin{aligned} \check{V}_{h}^{(0)}&=O(1)\Delta x\big (|\alpha _{r,2}|+|\omega _{h+1}|+|\Delta \sigma _{b_{h}}|+Q(\Lambda _b)\big )  &   (\text {see Case }2),&\\ {\hat{V}}_{h}^{(0)}&=O(1)\Delta x\big (|\alpha _{l,1}|+|\Delta \sigma _{\chi _{h}}|+Q(\Lambda _{s})\big )  &   (\text {see Case }3).&\end{aligned}$$

From Proposition 5.1, we obtain

6.7 $$\begin{aligned} \sum _{h=1}^{\infty }\big (\check{V}_{h}^{(0)}+{\hat{V}}_{h}^{(0)}\big )=O(1)\Delta xF(I_{1}). \end{aligned}$$

Then we write $\check{V}_{h}^{(1)}$ and ${\hat{V}}_{h}^{(1)}$ as

$$\begin{aligned} \check{V}_{h}^{(1)}&=\int _{y_{n_{b,h}-1}(h)}^{y_{n_{b,h}}(h)} \phi (x_{h},y_{n_{b,h}}(h))\\  &\quad \times \big (\rho (x_{h}-,y)u(x_{h}-,y) -{\check{\rho }}(x_{h}+,y)\check{u}(x_{h}+,y)\big ) \,{\textrm{d}}y\\&\quad +\int _{y_{n_{b,h}-1}(h)}^{y_{n_{b,h}}(h)} \phi (x_{h},y_{n_{b,h}}(h))\\&\quad \times \big ({\check{\rho }}(x_{h}+,y)\check{u}(x_{h}+,y)-\rho (x_{h}+,y)u(x_{h}+,y)\big )\,{\textrm{d}}y \end{aligned}$$

and

$$\begin{aligned} {\hat{V}}_{h}^{(1)}&=\int _{y_{n_{\chi ,h}}(h)}^{y_{n_{\chi ,h}+1}(h)} \phi (x_{h},y_{n_{\chi ,h}}(h))\\  &\quad \times \big (\rho (x_{h}-,y)u(x_{h}-,y) -{\hat{\rho }}(x_{h}+,y){\hat{u}}(x_{h}+,y)\big )\,{\textrm{d}}y\\&\quad +\int _{y_{n_{\chi ,h}}(h)}^{y_{n_{\chi ,h}+1}(h)} \phi (x_{h},y_{n_{\chi ,h}}(h))\\  &\quad \times \big ({\hat{\rho }}(x_{h}+,y){\hat{u}}(x_{h}+,y) -\rho (x_{h}+,y)u(x_{h}+,y)\big )\,{\textrm{d}}y, \end{aligned}$$

where

$$\begin{aligned}&\check{U}(x_{h}+,y)= {\tilde{U}}(\dfrac{y}{x_{h}};\dfrac{r_{h,n_{b,h}-1}}{x_{h}},U(x_{h}+,r_{h,n_{b,h}-1})),\\&{\hat{U}}(x_{h}+,y)={\tilde{U}}(\dfrac{y}{x_{h}};\dfrac{r_{h,n_{\chi ,h}-1}}{x_{h}},U(x_{h}+,r_{h,n_{\chi ,h}-1})), \end{aligned}$$

and ${\check{\rho }}$ and ${\hat{\rho }}$ are determined via Bernoulli’s equation. By the construction of the approximate solutions near the boundary and near the leading shock, we have

$$\begin{aligned}&\int _{y_{n_{b,h}-1}(h)}^{y_{n_{b,h}}(h)}\phi \big (x_{h},y_{n_{b,h}}(h)\big )\big ({\check{\rho }}(x_{h}+,y)\check{u}(x_{h}+,y)-\rho (x_{h}+,y)u(x_{h}+,y)\big ){\textrm{d}}y\\&\,\,\,\,\, =O(1)\Delta x\big (|\alpha _{r,2}|+|\omega _{h+1}|+|\Delta \sigma _{b_{h}}|+Q(\Lambda _b)\big )\quad (\text {see Case }2),&\\&\int _{y_{n_{\chi ,h}}(h)}^{y_{n_{\chi ,h}+1}(h)}\phi \big (x_{h},y_{n_{\chi ,h}}(h)\big )\big ({\hat{\rho }}(x_{h}+,y){\hat{u}}(x_{h}+,y)-\rho (x_{h}+,y)u(x_{h}+,y)\big ){\textrm{d}}y\\&\,\,\,\,\, =O(1)\Delta x\big (|\alpha _{l,1}|+|\Delta \sigma _{\chi _{h}}|+Q(\Lambda _{s})\big ) \quad (\text {see Case }3).&\end{aligned}$$

Similarly, by Proposition 5.1, we conclude that

6.8 $$\begin{aligned} \begin{aligned}&\sum _{h=1}^{\infty }\int _{y_{n_{b,h}-1}(h)}^{y_{n_{b,h}}(h)}\phi (x_{h},y_{n_{b,h}}(h))\big ({\check{\rho }}(x_{h}+,y)\check{u}(x_{h}+,y) -\rho (x_{h}+,y)u(x_{h}+,y)\big )\,{\textrm{d}}y\\&\,\,\,=O(1)\Delta xF(I_{1}),\\&\sum _{h=1}^{\infty }\int _{y_{n_{\chi ,h}}(h)}^{y_{n_{\chi ,h}+1}(h)} \phi (x_{h},y_{n_{\chi ,h}}(h))\big ({\hat{\rho }}(x_{h}+,y){\hat{u}}(x_{h}+,y)-\rho (x_{h}+,y)u(x_{h}+,y)\big )\,{\textrm{d}}y\\&\,\,\,=O(1)\Delta xF(I_{1}). \end{aligned} \end{aligned}$$

Set

$$\begin{aligned} {\bar{V}}_{h}&= \sum _{n=n_{\chi ,h}+1}^{n_{b,h}-1}\int _{y_{n-1}(h)}^{y_{n}(h)} \phi (x_{h},y_n(h))\big (\rho (x_{h}-,y)u(x_{h}-,y) -\rho (x_{h}+,y)u(x_{h}+,y)\big )\,{\textrm{d}}y\\&\quad +\int _{y_{n_{b,h}-1}(h)}^{y_{n_{b,h}}(h)} \phi (x_{h},y_{n_{b,h}}(h)) \big (\rho (x_{h}-,y)u(x_{h}-,y) -{\check{\rho }}(x_{h}+,y)\check{u}(x_{h}+,y)\big )\,{\textrm{d}}y\\&\quad +\int _{y_{n_{\chi ,h}}(h)}^{y_{n_{\chi ,h}+1}(h)} \phi (x_{h},y_{n_{\chi ,h}}(h))\big (\rho (x_{h}-,y)u(x_{h}-,y) -{\hat{\rho }}(x_{h}+,y){\hat{u}}(x_{h}+,y)\big )\,{\textrm{d}}y. \end{aligned}$$

As in [24] (see also [18]), let

$$\begin{aligned} H=\prod _{h=0}^{\infty }[0,1) =\{\vartheta =(\vartheta _{0},\vartheta _{1},\vartheta _{2},\cdots , \vartheta _{h},\cdots ):\,\vartheta _{h}\in [0,1),\ h=0,1,2,\cdots \}. \end{aligned}$$

Denoting ${\bar{y}}=y_{n-1}(h)+\vartheta _{h}\big (y_{n}(h)-y_{n-1}(h)\big )$, we obtain from (4.2) that

$$\begin{aligned}&\rho (x_{h}-,y)u(x_{h}-,y)-\rho (x_{h}+,y)u(x_{h}+,y)\\&\quad =\rho (x_{h}-,y)u(x_{h}-,y) -\rho (x_{h}-,{\bar{y}})u(x_{h}-,{\bar{y}})+\rho (x_{h}+,{\bar{y}})u(x_{h}+,{\bar{y}})\\&\qquad -\rho (x_{h}+,y)u(x_{h}+,y)\\&\quad =O(1)|\alpha |+O(1)|\alpha ||\Delta \sigma _\alpha |+O(1)|\Delta \sigma _{\alpha }|\\&\quad =O(1)(|\alpha |+|\Delta \sigma _\alpha |), \end{aligned}$$

where $\alpha$ is an elementary wave in $\Omega _{\Delta x,\vartheta ,h,j}$, and $\Delta \sigma _{\alpha }$ is the change of the $\sigma -$coordinate in the elementary wave $\alpha$. Denote the elementary waves in $\Omega _{\Delta x,\vartheta ,h}$ by $\alpha _{h,i}$. Then

6.9 $$\begin{aligned} {\bar{V}}_{h}=O(1)\Big (\sum _{i\leqq 0}|\alpha _{h,i}|+\sigma ^*-\sigma _*\Big )\Delta x, \end{aligned}$$

which implies

$$\begin{aligned} \sum _{h\geqq 1}\int _{H}{\bar{V}}_{h}^2{\textrm{d}}\vartheta =O(1)\text {diam}(\text {supp}\,\phi )\Big (\sum _{i\leqq 0}|\alpha _{h,i}|+\sigma ^*-\sigma _*\Big )^2\Delta x. \end{aligned}$$

$$\square $$

Next, we need the following lemma:

### Lemma 6.1

The approximate solutions $\{U_{\Delta x,\vartheta }(x,y)\}$ satisfy

6.10 $$\begin{aligned} \begin{aligned}&\int _{0}^1\int _{y_{n-1}(h)}^{y_n(h)} \big (U_{\Delta x,\vartheta }(x_{h}-,y)-U_{\Delta x,\vartheta }(x_{h}+,y)\big )\, {\textrm{d}}y {\textrm{d}}\vartheta _{h}\\&\quad =O(1)(\Delta x)^3+O(1)(|\alpha |+|\beta |)(\Delta x)^2. \end{aligned} \end{aligned}$$

### Proof

We now give a proof when $\alpha$ and $\beta$ are both shock waves, since the remaining cases can be obtained similarly.

Suppose that $\alpha$ and $\beta$ issue from $(x_{h-1},y_{n-1}(h-1))$ and $(x_{h-1},y_{n}(h-1))$, and end at $(x_{h},r_{1})$ and $(x_{h},r_{2})$, respectively. Set $a_{1}=\frac{r_{1}-y_{n-1}(h)}{y_{n}(h)-y_{n-1}(h)}$ and $a_{2}=\frac{r_{2}-y_{n-1}(h)}{y_{n}(h)-y_{n-1}(h)}$. From the construction of approximate solutions, we have

$$\begin{aligned}&\int _{0}^1\int _{y_{n-1}(h)}^{y_n(h)} \big (U_{\Delta x,\vartheta }(x_{h}-,y)-U_{\Delta x,\vartheta }(x_{h}+,y)\big )\,{\textrm{d}}y {\textrm{d}}\vartheta _{h}\\&\quad =\int _{0}^1\int _{y_{n-1}(h)}^{y_n(h)}U_{\Delta x,\vartheta }(x_{h}-,y)\,{\textrm{d}}y {\textrm{d}}\vartheta _{h}- \int _{0}^1\int _{y_{n-1}(h)}^{y_n(h)}U_{\Delta x,\vartheta }(x_{h}+,y)\,{\textrm{d}}y {\textrm{d}}\vartheta _{h}\\&\quad =\int _{y_{n-1}(h)}^{r_{1}}{\tilde{U}}(\frac{y}{x_{h}};\sigma _{2},U_{l})\,{\textrm{d}}y +\int _{r_{1}}^{r_{2}}{\tilde{U}}(\frac{y}{x_{h}};\sigma _{2},\Phi (0,\alpha _{2};U_{l}))\,{\textrm{d}}y\\&\quad \quad +\int _{r_{2}}^{y_n(h)}{\tilde{U}}(\frac{y}{x_{h}};\sigma _{1},\Phi (\beta _{1},0;{\tilde{U}}(\sigma _{1};\sigma _{2},\Phi (0,\alpha _{2};U_{l}))))\,{\textrm{d}}y\\&\quad \quad -a_{1}\int _{y_{n-1}(h)}^{y_n(h)}{\tilde{U}}(\frac{y}{x_{h}};\sigma _{2},U_{l})\,{\textrm{d}}y\\&\quad \quad -(a_{2}-a_{1})\int _{y_{n-1}(h)}^{y_n(h)}{\tilde{U}}(\frac{y}{x_{h}};\sigma _{2},\Phi (0,\alpha _{2};U_{l}))\,{\textrm{d}}y\\&\quad \quad -(1-a_{2})\int _{y_{n-1}(h)}^{y_n(h)}{\tilde{U}}(\frac{y}{x_{h}};\sigma _{1},\Phi (\beta _{1},0;{\tilde{U}}(\sigma _{1};\sigma _{2},\Phi (0,\alpha _{2};U_{l}))))\,{\textrm{d}}y. \end{aligned}$$

Since ${\tilde{U}}(\frac{y}{x_{h}};\sigma _{2},\Phi (0,\alpha _{2};U_{l})) ={\tilde{U}}(\frac{y}{x_{h}};{\tilde{U}}(\sigma _{1};\sigma _{2},\Phi (0,\alpha _{2};U_{l})))$, we obtain

$$\begin{aligned}&\int _{0}^1\int _{y_{n-1}(h)}^{y_n(h)}(U_{\Delta x,\vartheta }(x_{h}-,y)-U_{\Delta x,\vartheta }(x_{h}+,y))\,{\textrm{d}}y {\textrm{d}}\vartheta _{h}\\&\quad =\int _{y_{n-1}(h)}^{r_{1}}(1-a_{1})\big ({\tilde{U}}(\frac{y}{x_{h}};\sigma _{2},U_{l})-{\tilde{U}}(\frac{y}{x_{h}};\sigma _{2},\Phi (0,\alpha _{2};U_{l}))\big )\,{\textrm{d}}y\\&\quad \quad -\int _{y_{n-1}(h)}^{r_{1}}(1-a_{2})\big ({\tilde{U}}(\frac{y}{x_{h}};\sigma _{1},\Phi (\beta _{1},0;{\tilde{U}}(\sigma _{1};\sigma _{2},\Phi (0,\alpha _{2};U_{l}))))\\&\qquad \qquad \qquad -{\tilde{U}}(\frac{y}{x_{h}};{\tilde{U}}(\sigma _{1};\sigma _{2},\Phi (0,\alpha _{2};U_{l})))\big )\,{\textrm{d}}y\\&\quad \quad +\int _{r_{1}}^{r_{2}}a_{1}\big ({\tilde{U}}(\frac{y}{x_{h}};\sigma _{2},\Phi (0,\alpha _{2};U_{l}))-{\tilde{U}}(\frac{y}{x_{h}};\sigma _{2},U_{l})\big )\,{\textrm{d}}y\\&\quad \quad -\int _{r_{1}}^{r_{2}}(1-a_{2})\big ({\tilde{U}}(\frac{y}{x_{h}};\sigma _{1},\Phi (\beta _{1},0;{\tilde{U}}(\sigma _{1};\sigma _{2},\Phi (0,\alpha _{2};U_{l}))))\\&\qquad \qquad \qquad -{\tilde{U}}(\frac{y}{x_{h}};{\tilde{U}}(\sigma _{1};\sigma _{2},\Phi (0,\alpha _{2};U_{l})))\big )\,{\textrm{d}}y\\&\quad \quad +\int _{r_{2}}^{y_n(h)}a_{2}\big ({\tilde{U}}(\frac{y}{x_{h}};\sigma _{1},\Phi (\beta _{1},0;{\tilde{U}}(\sigma _{1};\sigma _{2},\Phi (0,\alpha _{2};U_{l}))))\\&\qquad \qquad \qquad -{\tilde{U}}(\frac{y}{x_{h}};{\tilde{U}}(\sigma _{1};\sigma _{2},\Phi (0,\alpha _{2};U_{l})))\big )\, {\textrm{d}}y\\&\quad \quad -\int _{r_{2}}^{y_n(h)}a_{1}\big ({\tilde{U}}(\frac{y}{x_{h}};\sigma _{2},U_{l})-{\tilde{U}}(\frac{y}{x_{h}};\sigma _{2},\Phi (0,\alpha _{2};U_{l}))\big )\,{\textrm{d}}y. \end{aligned}$$

Then, by Taylor’s expansion, we have

$$\begin{aligned}&{\tilde{U}}(\frac{y}{x_{h}};\sigma _{2},U_{l}) -{\tilde{U}}(\frac{y}{x_{h}};\sigma _{2},\Phi (0,\alpha _{2};U_{l}))\\&\quad =U_{l}-\Phi (0,\alpha _{2};U_{l})+A_{1}(y-y_{n-1}(h))+O(1)(y-y_{n-1}(h))^2, \\&{\tilde{U}}(\frac{y}{x_{h}};\sigma _{1},\Phi (\beta _{1},0;{\tilde{U}}(\sigma _{1};\sigma _{2},\Phi (0,\alpha _{2};U_{l})))) -{\tilde{U}}(\frac{y}{x_{h}};{\tilde{U}}(\sigma _{1};\sigma _{2},\Phi (0,\alpha _{2};U_{l})))\\&\quad =\Phi (\beta _{1},0;{\tilde{U}}(\sigma _{1};\sigma _{2},\Phi (0,\alpha _{2};U_{l}))) -{\tilde{U}}(\sigma _{1};\sigma _{2},\Phi (0,\alpha _{2};U_{l}))\\&\quad \quad +A_{2}(y-y_{n}(h))+O(1)(y-y_{n}(h))^2, \end{aligned}$$

with

$$\begin{aligned} A_{1}=&\left. \partial _y\Big ({\tilde{U}}(\frac{y}{x_{h}};\sigma _{2},U_{l})-{\tilde{U}}(\frac{y}{x_{h}};\sigma _{2},\Phi (0,\alpha _{2};U_{l}))\Big ) \right| _{y=y_{n-1}(h)},\\ A_{2}=&\left. \partial _y\Big ({\tilde{U}}(\frac{y}{x_{h}};\sigma _{1},\Phi (\beta _{1},0;{\tilde{U}}(\sigma _{1};\sigma _{2},\Phi (0,\alpha _{2};U_{l})))) \right. \\&\quad \left. -{\tilde{U}}(\frac{y}{x_{h}};{\tilde{U}}(\sigma _{1};\sigma _{2},\Phi (0,\alpha _{2};U_{l})))\Big ) \right| _{y=y_{n}(h)}. \end{aligned}$$

A direct computation leads to

$$\begin{aligned}&\int _{0}^1\int _{y_{n-1}(h)}^{y_n(h)} \big (U_{\Delta x,\vartheta }(x_{h}-,y)-U_{\Delta x,\vartheta }(x_{h}+,y)\big )\,{\textrm{d}}y {\textrm{d}}\vartheta _{h}\\&\quad = O(1)(y_{n}(h)-y_{n-1}(h))^3+\dfrac{1}{2}A_{1}(r_{1}-y_{n-1}(h))(r_{1}-y_{n}(h))\\&\quad \quad -\dfrac{1}{2}A_{2}(r_{2}-y_{n-1}(h))(r_{2}-y_{n}(h)). \end{aligned}$$

Noting that $A_{1}=O(1)|\alpha |$ and $A_{2}=O(1)|\beta |$, together with the Courant-Friedrichs-Lewy condition, we conclude (6.10). $\square$

Substituting $U_{\Delta x,\vartheta }$ in Lemma 6.1 by $\rho u$ and carrying out the same process leads to

$$\begin{aligned} \int _{0}^1{\bar{V}}_{h}{\textrm{d}}\vartheta _{h} =O(1)\Big (\text {diam}(\text {supp}\,\phi )+\sum _{i\leqq 0}|\alpha _{h,i}|\Big )(\Delta x)^2=O(1)(\Delta x)^2. \end{aligned}$$

As in (6.9), we obtain

$$\begin{aligned} {\bar{V}}_k=O(1)\Big (\sum _{i\leqq 0}|\alpha _{k,i}|+\sigma ^*-\sigma _*\Big )\Delta x. \end{aligned}$$

Then

$$\begin{aligned} \sum _{h>k}\int _{H}{\bar{V}}_{h} {\bar{V}}_k\,{\textrm{d}}\vartheta \leqq&\sum _{h>k}\left| \int _{0}^1 {\bar{V}}_{h}d\vartheta _{h}\right| \int _{0}^1| {\bar{V}}_k|\,{\textrm{d}}{\hat{\vartheta }}_{h} =O(1)\big (\text {diam}(\text {supp,}\phi )\big )^2\Delta x, \end{aligned}$$

where $${\textrm{d}}{\hat{\vartheta }}_{h} ={\textrm{d}}\vartheta _{0}\cdots {\textrm{d}}\vartheta _{h-1}{\textrm{d}}\vartheta _{h+1}\cdots $$.

Since

$$\begin{aligned} \Vert {\bar{V}}\Vert _{L^2(H)} =\sum _{h\geqq 1}\int _{H}{\bar{V}}_{h}^2{\textrm{d}}\vartheta +2\sum _{h>k}\int _{H}{\bar{V}}_{h}{\bar{V}}_k\,{\textrm{d}}\vartheta , \end{aligned}$$

we conclude that

$$\begin{aligned} \Vert {\bar{V}}\Vert _{L^2(H)}\rightarrow 0\qquad \text {as }\Delta x\rightarrow 0, \end{aligned}$$

which, combining with (6.7)–(6.8), gives a subsequence (still denoted by) $\{(u_m,v_m)\}$ such that ${\widetilde{V}}\rightarrow 0$ almost everywhere. Meanwhile, we have

$$\begin{aligned} \begin{aligned}&V_{h}-{\widetilde{V}}_{h}\\  &\quad = \sum _{n=n_{\chi ,h}+1}^{n_{b,h}-1}\int _{y_{n-1}(h)}^{y_n(h)}\big (\phi (x_{h},y_n(h))-\phi (x_{h},y)\big )\\  &\qquad \qquad \qquad \qquad \,\,\,\times \big (\rho (x_{h}-,y)u(x_{h}-,y)-\rho (x_{h}+,y)u(x_{h}+,y)\big )\,{\text {d}}y\\  &\quad \quad +\int _{y_{n_{b,h}-1}(h)}^{y_{n_{b,h}}(h)} \big (\phi (x_{h},y_{n_{b,h}}(h))-\phi (x_{h},y)\big )\\  &\qquad \qquad \qquad \,\,\,\,\times \big (\rho (x_{h}-,y)u(x_{h}-,y)-\rho (x_{h}+,y)u(x_{h}+,y)\big )\,{\text {d}}y\\  &\quad \quad +\int _{y_{n_{b,h}}(h)}^{b_{\Delta x,\vartheta }(x_{h})} \big (\phi (x_{h},y_{n_{b,h}+1}(h))-\phi (x_{h},y)\big )\\  &\qquad \qquad \qquad \quad \times \big (\rho (x_{h}-,y)u(x_{h}-,y)-\rho (x_{h}+,y)u(x_{h}+,y)\big )\,{\text {d}}y\\  &\quad \quad +\int _{\chi _{\Delta x,\vartheta }(x_{h})}^{y_{n_{\chi ,h}}(h)} \big (\phi (x_{h},y_{n_{\chi ,h}}(h))-\phi (x_{h},y)\big )\\  &\qquad \qquad \qquad \,\,\,\,\times \big (\rho (x_{h}-,y)u(x_{h}-,y)-\rho (x_{h}+,y)u(x_{h}+,y)\big )\,{\text {d}}y\\  &\quad \quad +\int _{y_{n_{\chi ,h}}(h)}^{y_{n_{\chi ,h}+1}(h)} \big (\phi (x_{h},y_{n_{\chi ,h}+1}(h))-\phi (x_{h},y)\big )\\  &\qquad \qquad \qquad \quad \,\times \big (\rho (x_{h}-,y)u(x_{h}-,y)-\rho (x_{h}+,y)u(x_{h}+,y)\big )\,{\text {d}}y\\  &\quad =O(1)\Delta x\sum _{n=n_{\chi ,h}+1}^{n_{b,h}-1}\int _{y_{n-1}(h)}^{y_n(h)}\big (\rho (x_{h}-,y)u(x_{h}-,y)-\rho (x_{h}+,y)u(x_{h}+,y)\big )\,{\text {d}}y \\  &\qquad +O(1)(\Delta x)^2\\&\quad =O(1)\Big (\sum _{i\leqq 0}|\alpha _{h,i}|+\sigma ^*-\sigma _*+1\Big )(\Delta x)^2, \end{aligned} \end{aligned}$$

which leads to

$$\begin{aligned} \text {IV}-{\widetilde{V}}=&\sum _{h\geqq 1}V_{h}-{\widetilde{V}}_{h} =O(1)\,\text {diam}(\text {supp}\,\phi )\Delta x. \end{aligned}$$

Thus, $\text {IV}\rightarrow 0$ as $m\rightarrow \infty$ for some subsequence $\{(u_m,v_m)\}$. $\square$

### Proposition 6.3

V, VI $$\rightarrow 0$$   as $$\Delta x\rightarrow 0$$.

### Proof

 Since

$$\begin{aligned} b'(x)=\frac{v(x_{h}+,b(x_{h})-)}{u\big (x_{h}+,b(x_{h})-\big )}\qquad \text { for }x\in (x_{h},x_{h+1}), \end{aligned}$$

it follows from the construction of our approximate solution that

$$\begin{aligned} v\big (x,b(x)\big )-u\big (x,b(x)\big )b'(x)=O(1)\Delta x. \end{aligned}$$

Therefore, we have

$$\begin{aligned} \text {V} =O(1)\,\text {diam}(\text {supp}\,\phi )\,\Delta x\rightarrow 0 \qquad \text {as }\Delta x\rightarrow 0. \end{aligned}$$

As for VI, we divide this term into three parts. The first part is the integral along the leading shock, where $W_{h,i}=S_{\Delta x,\vartheta ,h}$. For this part, by similar arguments in treating V, we have

$$\begin{aligned} \sum _{h}\int _{S_{\Delta x,\vartheta ,h}}\big (s_{h}(\rho ^+u^+-\rho ^-u^-)-(\rho ^+v^+-\rho ^-v^-)\big )\phi \,{\textrm{d}}x =O(1)\Delta x. \end{aligned}$$

The second part is the integral along the upper or lower edges of rarefaction waves and therefore vanishes automatically. The third part is the integral along the weak shock waves, that is, $W_{h,i}\ne S_{\Delta x,\vartheta ,h}$. In this case, by (4.1), we have

$$\begin{aligned}&\rho ^+u^+-\rho ^-u^-=(\rho ^+u^+-\rho ^-u^-)|_{x=x_{h}+}+O(1)(\rho ^+u^+-\rho ^-u^-)|_{x=x_{h}+}\Delta x,\\&\rho ^+v^+-\rho ^-v^-=(\rho ^+v^+-\rho ^-v^-)|_{x=x_{h}+}+O(1)(\rho ^+v^+-\rho ^-v^-)|_{x=x_{h}+}\Delta x. \end{aligned}$$

Thus, in view of the Rankine-Hugoniot conditions, we obtain

$$\begin{aligned} \sum _{i}(s_{h,i}(\rho ^+u^+-\rho ^-u^-)-(\rho ^+v^+-\rho ^-v^-))=O(1)\sum _{i}|\alpha _{S,h,i}|\Delta x, \end{aligned}$$

where $\alpha _{S,h,i}$ are the weak shock waves in $\Omega _{\Delta x,\vartheta ,h}$. Combining all the three parts together, we have

$$\begin{aligned} \text {IV} =O(1)\,\text {diam}(\text {supp}\,\phi )\,\sum _{i}|\alpha _{S,h,i}|\Delta x+O(1)\Delta x. \end{aligned}$$

By Proposition 5.1, $\sum _{i}|\alpha _{S,h,i}|$ is uniformly bounded with respect to *h*. Therefore, $\text {IV}\rightarrow 0$ as $\Delta x\rightarrow 0$. $\square$

With all the arguments stated above, a standard procedure as in [18, 38] gives the following theorem, which ensures the first part of the main theorem:

### Theorem 6.1

Suppose that **(A1)**–**(A2)**, $1<\gamma <3$, and $0<p_{0}<p^*$ hold. Then, when $M_{\infty }$ is sufficiently large, there are $\varepsilon _0>0$, a null set ${\mathcal {N}}$, and a constant $C>0$, depending only on $p_0$ and the system, such that if $T.V.\,\{p^{b}\}=\varepsilon _p<\varepsilon _0$, for each $\vartheta \in \prod _{h=0}^{\infty }[0,1)\backslash {\mathcal {N}}$, there exist both a subsequence $\{\Delta _{i}\}_{i=0}^{\infty }\subset \{\Delta x\}$ of the mesh size with $\Delta _{i}\rightarrow 0$ as $i\rightarrow \infty$ and a triple of functions $b_{\vartheta }(x)$ with $b_{\vartheta }(0)=0$, $\chi _{\vartheta }(x)$ with $\chi _{\vartheta }(0)=0$, and $U_{\vartheta }(x,y)\in O_{\varepsilon _{0}}\big (G(s_{0})\cap \mathbb {W}(p_{0},p_{\infty })\big )$ such that

(i) $b_{\Delta _{i},\vartheta }$ converges to $b_{\vartheta }$ uniformly in any bounded *x*-interval;
(ii) $\chi _{\Delta _{i},\vartheta }$ converges to $\chi _{\vartheta }$ uniformly in any bounded *x*-interval;
(iii) $b'_{\Delta _{i},\vartheta }$ converges to $(b'_{\vartheta })_+\in BV([0,\infty ))$ *a.e.*, satisfying
  $$\begin{aligned} \sup _{x>0}|(b'_{\vartheta })_+(x)-b_0|<C\varepsilon _p,\quad \quad b_{\vartheta }(x)=\int _0^x (b'_{\vartheta })_+(t){\textrm{d}}t\textrm{;} \end{aligned}$$
(iv) $s_{\Delta _{i},\vartheta }$ converges to $s_{\vartheta }\in BV([0,\infty ))$ *a.e.*, satisfying
  $$\begin{aligned} \sup _{x>0}|s_{\vartheta }(x)-s_0|<C\varepsilon _p,\quad \quad \chi _{\vartheta }(x)=\int _0^x s_{\vartheta }(t){\textrm{d}}t; \end{aligned}$$
(v) $U_{\Delta _{i},\vartheta }(x,\cdot )$ converges to $U_{\vartheta }\in {{\textbf {L}}}^1_{\textrm{loc}}(-\infty ,b_{\vartheta }(x))$ for every $x>0$, so that
  $$\begin{aligned} \begin{aligned} \sup _{x>0}\text{ T.V. }\{U_\vartheta (x,y)\, :\, \chi (x)<y<b(x)\}<C(\varepsilon _p+b_0-s_0), \end{aligned} \end{aligned}$$
  and $U_{\vartheta }$ is a global entropy solution of the inverse problem (1.1)–(1.2) and satisfies (1.8)–(1.9).

## Asymptotic Behavior of Global Entropy Solutions

To establish the asymptotic behavior of global entropy solutions, we need further estimates of the approximate solutions.

### Lemma 7.1

There exists a constant $M_{1}$, independent of $U_{\Delta x,\vartheta }$, $\Delta x$, and $\vartheta$, such that

7.1 $$\begin{aligned} \sum _{\Lambda }E_{\Delta x,\vartheta }(\Lambda )<M_{1} \end{aligned}$$

for $E_{\Delta x,\vartheta }(\Lambda )$ given as in (5.2).

### Proof

By Proposition 5.1, for any interaction region $\Lambda \subset \{(h-1)\Delta x\leqq (h+1)\Delta x\}$ for $h\geqq 1$, we have

$$\begin{aligned} \sum _{\Lambda }E_{\Delta x,\vartheta }(\Lambda )\leqq 4\sum _{\Lambda }\big (F(I)-F(J)\big )\leqq 4F(I_{1}). \end{aligned}$$

Thus, choosing $M_{1}=4F(I_{1})+1$, the proof is complete. $\square$

For any $t>0$, let ${\mathcal {L}}_{j,\vartheta }(t-)$, $j=1,2$, be the total variation of $j-$weak waves in $U_{\vartheta }$ crossing line $x=t$, and let ${\mathcal {L}}_{j,\Delta x, \vartheta }(t-)$, $j=1,2$, be the total variation of $j-$weak waves in $U_{\Delta x,\vartheta }$ crossing line $x=t$. Then we have

### Lemma 7.2

$$\sum _{j=1}^{2}{\mathcal {L}}_{j,\vartheta }(x-)\rightarrow 0$$ as $$x\rightarrow \infty $$.

### Proof

Let $U_{\Delta _{i},\vartheta }$ be a sequence of the approximate solutions introduced in Theorem 6.1, and let the corresponding term $E_{\Delta x,\vartheta }(\Lambda )$ be defined in (5.2). As in [25], denoted by ${\textrm{d}}E_{\Delta x,\vartheta }$ the measure of assigning quantities $E_{\Delta x,\vartheta }(\Lambda )$ to the center of $\Lambda$. Then, by Lemma 7.1, we can choose a subsequence (still denoted as) ${\textrm{d}}E_{\Delta _{i},\vartheta }$ such that

$$\begin{aligned} {\textrm{d}}E_{\Delta _{i},\vartheta }\rightarrow dE_{\vartheta } \qquad \text {as } \Delta _{i}\rightarrow 0 \end{aligned}$$

with $$E_{\vartheta }(\Lambda )<\infty $$.

Therefore, for $\varepsilon _{1}>0$ sufficiently small, we can choose $x_{\varepsilon _{1}}$ (independent of $U_{\Delta _{i},\vartheta }$), $\Delta _{i}$, and $\vartheta$ such that

$$\begin{aligned} \sum _{h>[x_{\varepsilon _{1}}/\Delta x]} E_{\Delta _{i},\vartheta }(\Lambda _{h,n})<\varepsilon _{1}. \end{aligned}$$

Let $X_{\varepsilon _{1}}^{1}=(x_{\varepsilon _{1}},\chi _{\Delta _{i},\vartheta }(x_{\varepsilon _{1}}))$ and $X_{\varepsilon _{1}}^{2}=(x_{\varepsilon _{1}},b_{\Delta _{i},\vartheta }(x_{\varepsilon _{1}}))$ be the two points lying in the approximate leading shock $y=\chi _{\Delta _{i},\vartheta }(x)$ and the approximate boundary $y=b_{\Delta _{i},\vartheta }(x)$, respectively. Let $\chi _{\Delta _{i},\vartheta }^{j}$ be the approximate *j*–generalized characteristic issuing from $X_{\varepsilon _{1}}^{j}$ for $j=1,2$, respectively. According to the construction of the approximate solutions, there exist constants ${\hat{M}}_{j}>0$, $j=1,2$, independent of $U_{\Delta _{i},\vartheta }$, $\Delta _{i}$, and $\vartheta$, such that

$$\begin{aligned} |\chi _{\Delta _{i},\vartheta }^{j}(x_{1})-\chi _{\Delta _{i},\vartheta }^{j}(x_{2})|\leqq {\hat{M}}_{j}\big (|x_{1}-x_{2}|+\Delta _{i}\big ) \qquad \text {for } x_{1},x_{2}>x_{\varepsilon _{1}}. \end{aligned}$$

Then we choose a subsequence (still denoted by) $\Delta _{i}$ such that

$$\begin{aligned} \chi _{\Delta _{i},\vartheta }^{j}\rightarrow \chi _{\vartheta }^{j}\qquad \text {as } \Delta _{i}\rightarrow 0 \end{aligned}$$

for some $\chi _{\vartheta }^{j}\in$ Lip with $(\chi _{\vartheta }^{j})'$ bounded.

Let two characteristics $\chi _{\vartheta }^{1}$ and $\chi _{\vartheta }^{2}$ intersect with the cone boundary $\Gamma _{\vartheta }$ and the leading shock $S_{\vartheta }$ at points $(t_{\varepsilon _{1}}^1,\chi _{\vartheta }^{1}(t_{\varepsilon _{1}}^1))$ and $(t_{\varepsilon _{1}}^2,\chi _{\vartheta }^{2}(t_{\varepsilon _{1}}^2))$ for some $t_{\varepsilon _{1}}^1$ and $t_{\varepsilon _{1}}^2$, respectively. Then, as in [25], we apply the approximate conservation law to the domain below $\chi _{\Delta _{i},\vartheta }^{1}$ and above $\chi _{\Delta _{i},\vartheta }^{1}$ and use Lemma 7.1 to obtain

$$\begin{aligned} {\mathcal {L}}_{j,\Delta _{i},\vartheta }(x-)\leqq C\sum _{h>[x_{\varepsilon _{1}}/\Delta x]} E_{\Delta _{i},\vartheta }(\Lambda _{h,n})<C\varepsilon _{1} \end{aligned}$$

for $j=1,2$, $x>t_{\varepsilon _{1}}^1+t_{\varepsilon _{1}}^2$. This completes the proof. $\square$

### Theorem 7.1

For $$p^{b}_{\infty }:=\lim _{x\rightarrow \infty }p^{b}(x)$$, $$s_{\infty }:=\lim _{x\rightarrow \infty }s_{\vartheta }(x)$$, and $$b'_{\infty }=\lim _{x\rightarrow \infty }(b_{\vartheta })'_{+}(x)$$,

$$\begin{aligned}&\lim _{x\rightarrow \infty }\sup \left\{ \big |U_{\vartheta }(x,y)-{\tilde{U}}(\sigma ;s_{\infty },G(s_{\infty }))\big |\,:\,\chi _{\vartheta }(x)<y<b_{\vartheta }(x)\right\} =0,\\&\dfrac{1}{2}\big |{\tilde{U}}(b_{\infty }';s_{\infty },G(s_{\infty }))\big |^2+\dfrac{\gamma (p^{b}_{\infty })^{\frac{\gamma -1}{\gamma }}}{\gamma -1}=\dfrac{1}{2}+\dfrac{\gamma p_{\infty }^{\frac{\gamma -1}{\gamma }}}{\gamma -1},\\&{\tilde{U}}(b_{\infty }';s_{\infty },G(s_{\infty }))\cdot (-b_{\infty }',1)=0. \end{aligned}$$

### Proof

For every $x\in [x_{k-1},x_{k})$, we have

$$\begin{aligned}&\big |U_{\vartheta }(x,y)-{\tilde{U}}(\sigma ;s_{\Delta _{i},\vartheta },G(s_{\Delta _{i},\vartheta }))\big | +\big |{\tilde{U}}(b_{\Delta _{i},\vartheta }';s_{\Delta _{i},\vartheta },G(s_{\Delta _{i},\vartheta }))\cdot (-b_{\Delta _{i},\vartheta }',1)\big |\\&\quad \quad +\Big |\dfrac{1}{2}\big |{\tilde{U}}(b_{\Delta _{i},\vartheta }';s_{\Delta _{i},\vartheta },G(s_{\Delta _{i},\vartheta }))\big |^2 +\dfrac{\gamma (p^{b}_{\Delta x,k})^{\frac{\gamma -1}{\gamma }}}{\gamma -1}-\dfrac{1}{2}-\dfrac{\gamma p_{\infty }^{\frac{\gamma -1}{\gamma }}}{\gamma -1}\Big |\\&\quad \leqq C \Big (\sum _{j=1}^{2}{\mathcal {L}}_{j,\Delta _{i},\vartheta }(x-)+|\Delta _{i}|\Big ). \end{aligned}$$

By Theorem 6.1, letting $i\rightarrow \infty$, we obtain

$$\begin{aligned}&\sup _{\chi _{\vartheta }(x)<y<b_{\vartheta }(x)} \big |U_{\vartheta }(x,\cdot )-{\tilde{U}}\big (\sigma ;s_{\vartheta },G(s_{\vartheta })\big )\big | +\big |{\tilde{U}}((b_{\vartheta })_{+}';s_{\vartheta },G(s_{\vartheta }))\cdot (-b_{\vartheta }',1)\big |\\&\quad \quad +\Big |\dfrac{1}{2}\big |{\tilde{U}}((b_{\vartheta })_{+}';s_{\vartheta },G(s_{\vartheta }))\big |^2+\dfrac{\gamma (p^{b})^{\frac{\gamma -1}{\gamma }}}{\gamma -1}-\dfrac{1}{2}-\dfrac{\gamma p_{\infty }^{\frac{\gamma -1}{\gamma }}}{\gamma -1}\Big |\\&\quad \leqq C \sum _{j=1}^{2}{\mathcal {L}}_{j,\vartheta }(x-). \end{aligned}$$

Then, using Lemma 7.2 and noting that ${\tilde{U}}(\sigma ;s,G(s))$ is a continuous function with respect to $\sigma$ and *s*, we conclude our result. $\square$

## Data Availability

Not applicable.
